# Reproducible Research Practices and Transparency across the Biomedical Literature

**DOI:** 10.1371/journal.pbio.1002333

**Published:** 2016-01-04

**Authors:** Shareen A. Iqbal, Joshua D. Wallach, Muin J. Khoury, Sheri D. Schully, John P. A. Ioannidis

**Affiliations:** 1 Department of Epidemiology, Rollins School of Public Health, Emory University, Atlanta, Georgia, United States of America; 2 Department of Health Research and Policy, Stanford School of Medicine, Palo Alto, California, United States of America; 3 Meta-Research Innovation Center at Stanford, Stanford University, Stanford, California, United States of America; 4 Division of Cancer Control and Population Sciences, National Cancer Institute, National Institutes of Health, Bethesda, Maryland, United States of America; 5 Office of Public Health Genomics, Centers for Disease Control and Prevention, Atlanta, Georgia, United States of America; 6 Stanford Prevention Research Center, Department of Medicine, Stanford University, Stanford, California, United States of America; 7 Department of Statistics, Stanford University School of Humanities and Sciences, Stanford, California, United States of America; Walter and Eliza Hall Institute of Medical Research, AUSTRALIA

## Abstract

There is a growing movement to encourage reproducibility and transparency practices in the scientific community, including public access to raw data and protocols, the conduct of replication studies, systematic integration of evidence in systematic reviews, and the documentation of funding and potential conflicts of interest. In this survey, we assessed the current status of reproducibility and transparency addressing these indicators in a random sample of 441 biomedical journal articles published in 2000–2014. Only one study provided a full protocol and none made all raw data directly available. Replication studies were rare (*n* = 4), and only 16 studies had their data included in a subsequent systematic review or meta-analysis. The majority of studies did not mention anything about funding or conflicts of interest. The percentage of articles with no statement of conflict decreased substantially between 2000 and 2014 (94.4% in 2000 to 34.6% in 2014); the percentage of articles reporting statements of conflicts (0% in 2000, 15.4% in 2014) or no conflicts (5.6% in 2000, 50.0% in 2014) increased. Articles published in journals in the clinical medicine category versus other fields were almost twice as likely to not include any information on funding and to have private funding. This study provides baseline data to compare future progress in improving these indicators in the scientific literature.

## Abstract 2

Examination of recent trends in reproducibility and transparency practices in biomedical research reveals an ongoing lack of access to full datasets and detailed protocols for both clinical and non-clinical studies.

## Author Summary

There is increasing interest in the scientific community about whether published research is transparent and reproducible. Lack of replication and non-transparency decreases the value of research. Several biomedical journals have started to encourage or require authors to submit detailed protocols, full datasets, and disclose information on funding and potential conflicts of interest. In this study, we investigate the reproducibility and transparency practices across the full spectrum of published biomedical literature from 2000–2014. We identify an ongoing lack of access to full datasets and detailed protocols for both clinical and non-clinical biomedical investigation. We also map the availability of information on funding and conflicts of interest in this literature. The results from this study provide baseline data to compare future progress in improving these indicators in the scientific literature. We believe that this information may be essential to sensitize stakeholders in science about the need for improving reproducibility and transparency practices.

## Introduction

The inability to replicate published research has been an ongoing concern in the scientific community [1]. There is clear evidence from basic molecular and animal modeling research that a large portion of published articles lack reproducibility [2], which could potentially be related to the increase in lack of efficacy in clinical trials [3,4]. It has been suggested that the inability to replicate findings is due to a lack of research transparency [5]. Recently, there has been a growing movement to encourage making protocols, analytical codes, and data openly available [6–8]. In this study, we aimed to assess the current status of reproducibility and transparency in a random sample of published biomedical journal articles and to derive empirical data on indicators that have been proposed as being important to monitor in this regard [9], i.e., the proportion of studies sharing protocols and raw data, undergoing rigorous independent replication and reproducibility checks, and reporting conflicts of interest and sources of public and/or private funding.

## Results

### Description of Assessed Sample of Articles

A total of 441 (88.2%), from the original randomly selected 500 articles were publications in eligible research fields directly related to biomedicine. Of these, two-thirds had some form of empirical data (*n* = 304 (68.9%)—*n* = 268 excluding case studies and case series, in which protocols and raw data sharing may not be pertinent, and *n* = 259 excluding also systematic reviews, meta-analyses and cost-effectiveness analyses where replication in studies with different data would not be pertinent). Among the 441 eligible studies, four (0.9%) were cost effectiveness or decision analyses, 36 (8.2%) were case studies or case series, 15 (3.4%) were randomized clinical trials, five (1.1%) were systematic reviews or meta-analyses, and 244 (55.3%) were other articles with empirical data (including cross-sectional, case-control, cohort, and various other uncontrolled human or animal studies). Just over 30% of the articles were classified as research without empirical data or models/modeling studies. Less than one in five articles (19.2%) had open full-text access from PubMed Central and about half (47.8%) of the papers belonged to the journal category of clinical medicine (Table 1).

**Table 1:** Characteristics of assessed articles.

	All Studies:	Research Articles with Empirical Data Only:
	*n* = 441	*n* = 304
Characteristics	Median (IQR)	Median (IQR)
	*n* (%)	*n* (%)
**Impact Factor (2013)**		
*Impact Factor—Median*		
	3.2 (1.9, 5.1)	3.2 (1.9, 4.7)
*Impact Factor—Categorized*		
0–2	107 (24.3)	77 (25.3)
>2–4	141 (32.0)	107 (35.2)
>4–6	73 (16.6)	59 (19.4)
>6	77 (17.5)	42 (13.8)
No 2013 JCR Impact Factor Listed	43 (9.8)	19 (6.3)
**Articles with PCMIDs**		
	85 (19.2)	67 (22.0)
**Article Study Field by Journal Category**		
Agricultural Sciences	9 (2.0)	5 (1.6)
Biology and Biochemistry	52 (11.8)	41 (13.5)
Clinical Medicine	211 (47.8)	144 (47.4)
Environment/Ecology	14 (3.2)	12 (4.0)
Immunology	10 (2.3)	7 (2.3)
Microbiology	9 (2.0)	8 (2.6)
Molecular Biology and Genetics	21 (4.8)	17 (5.6)
Neuroscience and Behavior	36 (8.2)	25 (8.2)
Pharmacology and Toxicology	21 (4.8)	14 (4.6)
Plant and Animal Science	16 (3.6)	10 (3.3)
Psychiatry/Psychology	16 (3.6)	9 (3.0)
Social Sciences, General	26 (5.9)	12 (4.0)

### Protocol Availability

Excluding case studies or case series (in which a protocol would not be relevant) 267 (99.6%) of the 268 papers with empirical data did not include a link to a full study protocol. Only one article had a protocol; in fact, the article was itself the protocol of a trial, and it was published in the open-access journal *Trials* (A221). Another five studies either referenced their clinical trials identifier and included a link to ClinicalTrials.gov (A281, A434), provided only Clinical Trials identifiers (A407, A477), or stated that a Clinical Trials repository link was available on the journal website (A261), but none of these articles or their links contained information about a full protocol.

There were seven other articles that had additional methods sections, figures, brief analytical plans and/or considerations, or supplementary materials either as a detailed appendix at the end of the paper (A434) or online (A25, A35, A174, A376, A290, A361 [contained an error message for page not found]). However, none of these supplementary materials fit our pre-specified definition of publicly available full or partial protocols.

### Raw Data Availability

Of the 268 articles with empirical data (excluding case studies and case series) none provided access to all the raw data involved in the study. One article contained information on how to request a complete dataset (A287), two papers listed a non-functioning online link for supplementary data, data elements, or findings (A330, A361), and another four articles had supplementary files or links to some absorption spectra figures and/or data (A35, A117, A130, A305), but not to the entire raw data used in the paper.

### Funding

**Fig 1:**
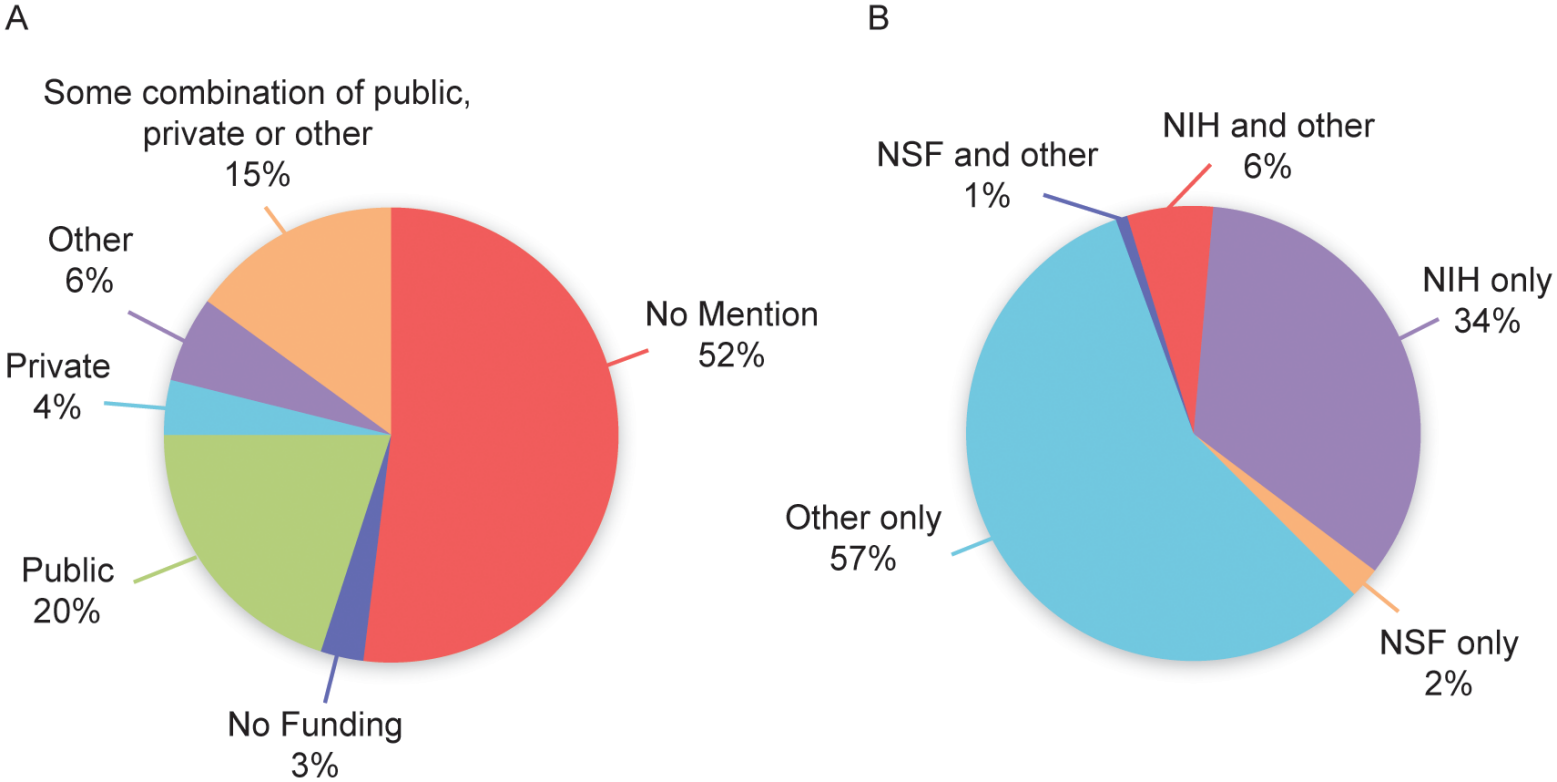
Distribution of funding overall (A) or among publically funded articles (B).

About half (51.7%) of the 441 biomedical articles did not include any information on funding and about a third (34.7%) were publically funded either alone or in combination with other funding sources. Of the 153 publically funded articles, 62 had National Institutes of Health (NIH) funding and four received National Science Foundation (NSF) support, alone or in combination with other sources of funding (Fig 1). There was no major change in the pattern of sources of funding over the 15-y period (S5 Fig).

### Articles Claiming to Contain Novel Findings Versus Replication Efforts

Of the 259 biomedical articles with empirical data, excluding case studies and case series, systematic reviews/meta-analyses, and cost effectiveness/decision analysis studies, only four (1.5%) clearly claimed or were inferred to be replication efforts trying to validate previous knowledge. Over half (51.7%) of the studies claimed to present some novel findings and four (1.5%) had clear statements of both study novelty and some form of replication. There were 117 (45.2%) articles that either had no statement or an unclear statement in the abstract and introduction about whether there were any novel findings or replication efforts.

### Subsequent Citing by Replication Studies

For the 259 biomedical articles with empirical data, excluding case studies and case series, systematic reviews/meta-analyses, and cost effectiveness/decision analysis studies, we also assessed whether any subsequently published papers had cited the article, mentioning that the authors were attempting to replicate part or all of their findings. Eight articles (3.1%) in the final dataset had at least some portion of their findings replicated (A11, A59, A129, A222, A278, A285, A407, A441), while the remaining 251 articles had no citing article that claimed to be a replication. Five of these eight articles were from the clinical medicine journal category. Of the replicating articles, one was unable to reproduce the results from the original article (A59), but mentioned that different definitions were used [10]. Three articles had their results replicated through different methodology [11] (A222), [12] (A278), [13] (A407). One article (A11) had several subsequent studies that either confirmed portions of the original study [14] or failed to validate certain previous findings [15]. Two studies developed new methodology that the replicating studies confirmed (A285), either by comparing to available methods [16] or a newly developed method [17] (A441). One article (A129) was cited by a subsequent study by the same first author [18] that stated that one of their aims was to test a hypothesis from earlier observations with longer observation and modeling techniques.

### Citation and Inclusion of Data in Systematic Reviews and/or Meta-analyses

In order to measure whether empirical studies are eventually integrated in systematic reviews, the 259 articles with empirical data (again excluding case studies and case series, systematic reviews/meta-analyses, and cost effectiveness/decision analysis studies) were assessed on whether they had been cited at least once in subsequent systematic reviews and/or meta-analyses. Empirical data from 16 articles (6.2%) were utilized in a systematic review/meta-analysis (A89, A93, A105, A157, A190, A222, A261, A268, A270, A278, A338, A340, A374, A407, A421, A477). At least one systematic review/meta-analysis cited another three articles but provided reasons for not including any of their data in a quantitative synthesis for any outcome (A83, A129, A221). Yet another 19 articles were cited incidentally by systematic reviews/meta-analyses (e.g., in introduction or discussion, but without having data considered in quantitative syntheses for any outcome) (A5, A28, A31, A112, A203, A207, A224, A256, A274, A319, A322, A327, A377, A400, A413, A433, A435, A453, A463). Lastly, there were 221 articles (85.3%) that were not cited in any systematic reviews/meta-analyses.

### Reporting of Conflicts of Interest

The large majority of the 441 articles had no conflict of interest statement (305 [69.2%]). Of the remaining, 110 (24.9%) did not report any conflicts of interest and 26 (5.9%) reported conflicts of interest. For the 15 randomized controlled trials, eight articles (53.3%) reported no conflicts of interest, four (26.7%) articles had no statement of conflict, and three (20.0%) articles had a clear statement of conflict.

**Fig 2:**
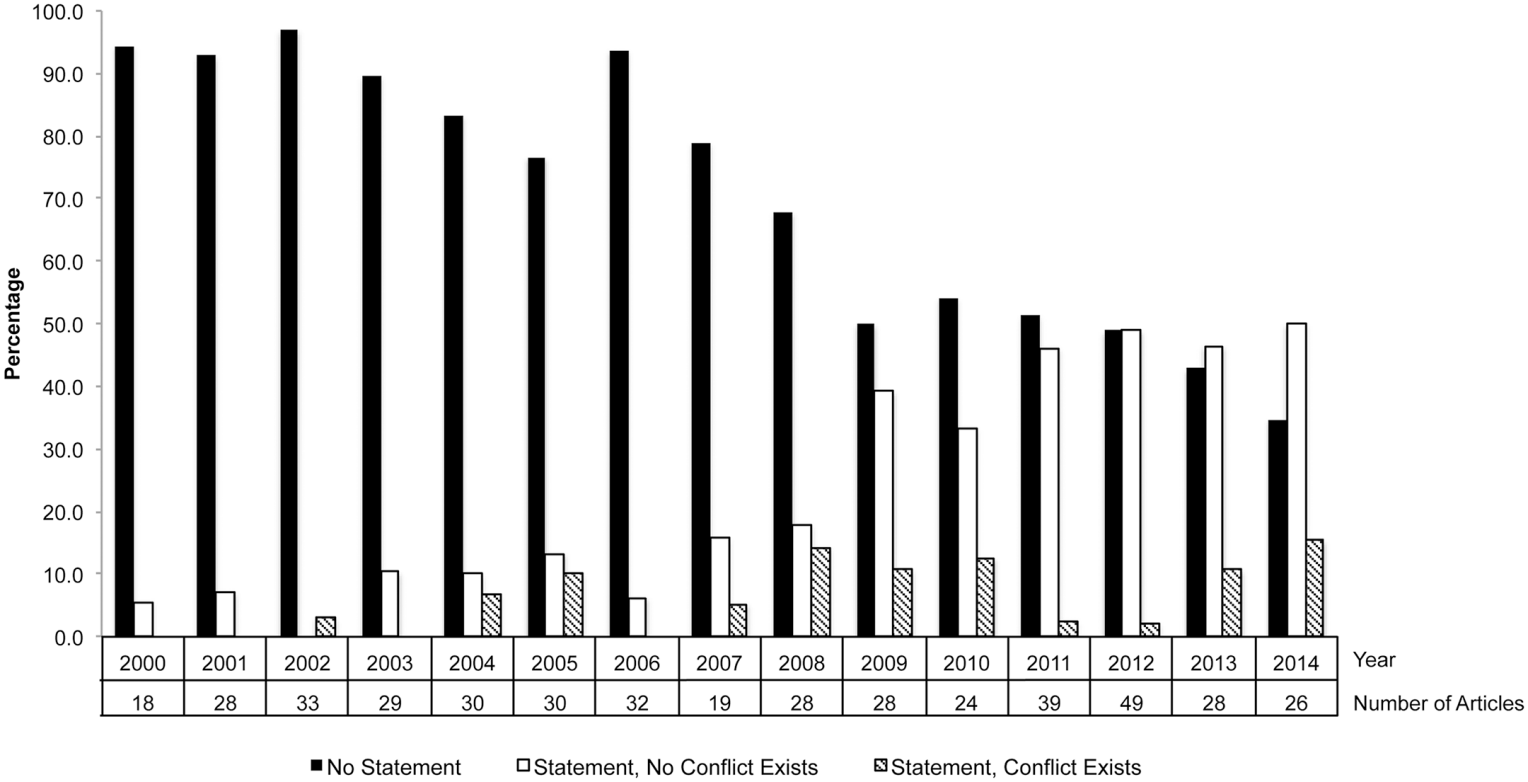
Trends in presence of statements of conflicts of interest.

Between 2000 and 2014, the percentage of articles with no statement of conflict decreased substantially (94.4% in 2000 to 34.6% in 2014), whereas the number of articles reporting statements of conflicts (0% in 2000, 15.4% in 2014) or no conflicts (5.6% in 2000, 50.0% in 2014) increased (Fig 2).

### Comparison of Clinical Medicine Versus Other Biomedical Fields

A comparison of articles published in journals in the clinical medicine category versus other fields showed some distinctive patterns (Table 2). Articles in the clinical medicine journal category were almost twice as likely to not include any information on funding and to have private funding, while they were far less likely to have public funding or funding from different types of sources (public and/or private and/or other). Articles in the clinical medicine journal category were also more likely to contain no statement on novelty or replication and less likely to claim novel study findings than articles in the “other” journal category. Furthermore, articles in the clinical medicine journal category were less likely to have full open access compared to other fields of study. There were no significant differences between replication, article citation for systematic review and/or meta-analysis, and statements of conflict (Table 2).

**Table 2:** Articles in the clinical medicine journal category versus other journal categories.

Variables	Clinical Medicine	Other	*p*-value*
	*n* (%)	*n* (%)	
**Funding**	***n* = 211**	***n* = 230**	**<0.0001**
No Mention	142 (67.3)	86 (37.4)	
No Funding	9 (4.3)	3 (1.3)	
Public	17 (8.1)	69 (30.0)	
Private	13 (6.2)	6 (2.6)	
Other	12 (5.7)	16 (7.0)	
Some combination of Public, Private, or Other	18 (8.5)	50 (21.7)	
**Replication**	***n* = 110**	***n* = 149**	**0.0116**
Novel Findings	46 (41.8)	88 (59.1)	
Replication	3 (2.7)	1 (0.7)	
Novel Findings and Replication	1 (0.9)	3 (2.0)	
No Statement on Novelty or Replication	60 (54.6)	57 (38.3)	
**Article Citation**	***n* = 110**	***n* = 149**	
*Replication of Index Study*			*0*.*2903*
No Citing Article	105 (95.4)	146 (98.0)	
At Least One Citing Article	5 (4.6)	3 (2.0)	
*Systematic Review/Meta-Analysis*			*0*.*4431*
No Citing Article	90 (81.8)	131 (87.9)	
At Least One Citing Article, No Data Included	11 (10.0)	8 (5.4)	
At Least One Citing Article, Data Excluded	1 (0.9)	2 (1.3)	
At Least One Citing Article, Data Included	8 (7.3)	8 (5.4)	
**Statement of Conflict**	***n* = 211**	***n* = 230**	**0.2397**
No Statement	138 (65.4)	167 (72.6)	
Statement, No Conflict Exists	15 (7.1)	11 (4.8)	
Statement, Conflict Exists	58 (27.5)	52 (22.6)	
**PMCID**	***n* = 211**	***n* = 230**	**0.0025**
Articles with PMCID	28 (13.3)	57 (24.8)	

*Based on Fisher-Freeman-Halton exact test with Monte Carlo approximation.

When further limited to the 304 articles with empirical data, articles in the clinical medicine category journals were more likely to not mention funding (59.0% versus 26.3%) and less likely to have a PubMed Central reference number (PMCID) (16.0% versus 27.5%). There was no significant difference in the proportion of articles not including statements of conflicts of interest (65.3% [clinical medicine] versus 71.3% [other]).

## Discussion

Our empirical evaluation shows that the published biomedical literature lacks transparency in important dimensions. We found a full protocol only for one study in our sample. In basic science exploratory research, formal protocols may not be available ahead of time. However, a post-research detailed protocol should be provided. It is unclear how many biomedical papers have no protocols versus do have protocols but do not make them publicly available. During the earlier years of the sampling time frame, there may not have been many online protocol-sharing repositories, such as OpenWetWare [19], which was created in 2005. However, authors could have included a statement about the availability of their protocol either upon request or on a personal or laboratory website.

Previous evaluations have identified common inconsistencies between available protocols and final publications of randomized trials [20,21]. For other types of study designs, such comparisons are hampered by the rare availability of protocols. Public protocol sharing not only provides external researchers ways to find possible discrepancies between final publications and research plans [9], more importantly, it allows study designs and experiments to be reproduced by interested scientists.

Of the 268 biomedical articles with empirical data assessed (excluding case studies and case series), none had open access to all the raw data. A previous evaluation found 9% of articles published in the 50 journals with the highest impact factor in 2009 had deposited full primary data online [21]. Our results may differ due to the fact that we focused on the full spectrum of biomedical journals in PubMed (median impact factor 3.2). Data sharing requirements have changed over the last few years, especially in high-impact journals [21,22], but these represent only a small fraction of the journals studied here. Although one article in our study claimed that the complete dataset was available upon request (A287), a statement of willingness to share may not guarantee that the data will be available to independently requesting scientists [23]. Sponsor priorities, lack of resources, personal investigator opinions, and proprietary perceptions may influence data withholding [24,25]. Six other articles included supplementary files or links with some additional data, but for two of them, the links were nonfunctioning (A330, A361). Evidence exists that even the most prestigious journals have supplementary information that eventually became unavailable [26].

Although the NIH reaffirmed their support for the concept of data sharing in 2003 by stating that applications seeking $500,000 or more in direct costs for a single year are expected to include plans for sharing data or statements why data sharing would not be possible [27], there is no evidence of a major change in data sharing practices between 2003 and 2014 when the entire PubMed-indexed literature is considered. According to the NSF Data Sharing Policy in 2011, investigators have been expected to share the primary data and other supplementary materials created or gathered under NSF grants with other researchers within a reasonable time frame [28]. With only a few studies funded by NSF in this sample of biomedical articles, it is not possible to determine whether this policy has had any impact over time. The sharing of raw data and protocols will be facilitated by the emergence of more available options and repositories, but this plethora of choices may need to be streamlined at some point. Investigators may also continue to use their preferred method of sharing.

The majority of papers claimed to present some novel discoveries. However, we suspect that very few papers truly have totally, disruptively innovative findings. Instead they may be operating in knowledge space where other past studies may also have operated, but they still claim novelty. It is difficult to probe objectively how much innovation is needed to be able to claim novelty. Moreover, none of the subsequently published papers that cited the original articles and mentioned that the authors were attempting to replicate part or all of their findings were full study replication attempts. Replication has been accepted as a sine qua non in a few disciplines, such as human genome epidemiology, but those disciplines are the exception. When some effort at replication is done, investigators may still try to differentiate their replication study as being different from the original and, thus, also make a case for novelty. There are many different proposals on how reproducible research can be guaranteed. These include approaches at reproducible practices, i.e., making other investigators able to repeat the process and calculations [29]; re-analysis (as in the case of randomized trials [30]); and replication by independent investigators, as in genetics, psychology, and cancer biology [31–33]. We also demonstrated that very few primary data are currently included in systematic reviews and meta-analyses. Despite the advent of evidence-based medicine, these data syntheses still cover only small fractions of the available evidence.

Previous studies have found that between 29% and 69% of published clinical research articles had some type of financial conflict [34,35], and a survey of NIH-funded life science researchers found that 43% of 2,167 respondents reported receiving some research-related contribution, such as reagents, equipment, travel funds, etc. [36]. In our study, which covers a very wide range of research designs and types, only 5.9% of the articles had conflict of interest statements. This is likely an underestimate of the prevalence of conflicts in biomedical research and could be a result of the lack of conflict of interest disclosure policies among many journals [37]. However, we also found that the number of statements reporting no conflicts of interest increased and, conversely, the number of articles without any statements decreased over time, perhaps due to strengthening of certain journal disclosure policies [37]. The persisting high prevalence of no statement of conflict is, nevertheless, worrisome. Conflicted stakeholders can operate in a stealth mode and have a significant impact on the design, conduct, and analysis of biomedical studies [1,38,39].

Slightly over half of the analyzed papers reported no funding. It is possible that some of them simply did not mention existing sponsors. Still, a large share of the published literature occurs without any support, and this should cause some concern. Public funding is listed for about a third of the 441 biomedical papers and NIH accounts for a mere 14% of the total biomedical literature. The challenge is even greater for clinical research in particular: only 9.0% of published papers in journals in the clinical medicine category mentioned NIH funding, and more than 70% of papers in this category mentioned no funding or clearly state that they had no funding at all. Underfunding in combination with conflicted sponsored funding creates a difficult situation for clinical research.

### Limitations

Our evaluation is limited to published biomedical research information. In theory, sometimes one may be able to obtain additional raw data and protocols, and clarifications on conflicts or funding by communicating with the authors or sponsors. However, the yield would be uncertain, and personal communications should not replace the lack of transparency in the published scientific record. Furthermore, the fact that we only used the published records means that we could not correct any inaccuracies in the claims of the original authors. This may be particularly prominent in the case of claims for novelty, in which some authors may have tried to sell their paper as being more novel than it really is, so as to make it more attractive for publication. Although the two investigators (SAI and JDW) used their best judgment and discussed all eligible papers before agreeing upon a final classification, certain decisions may have been subjective. In particular, when determining study novelty and replication for articles from diverse biomedical fields, difficulty arose assessing whether study results were truly groundbreaking or being fully replicated. In order to account for these limitations, all ambiguous articles were discussed with a third reviewer (JPAI).

### Conclusions

We hope that our survey will further sensitize scientists, funders, journals, and other stakeholders in science to the need to improve these indicators. There are several efforts to improve reproducibility [40–42]. By continuing to monitor these indicators in the future, it is possible to track any evidence of improvement in the design, conduct, analysis, funding, and independence of biomedical research over time.

## Materials and Methods

### Sample of Assessed Papers

A sample of 500 English-language journal articles published between 2000 and 2014 was chosen randomly based on PubMed identification (PMID) numbers. PMID numbers ranging from 10,000,000 to 25,000,000 were inputted into OpenEpi (version 3.02) random number generator to select a random sample of 750 PMID numbers. Beginning from the first number generated, each number was verified for eligibility in sequence until 500 eligible PMID numbers were chosen. Of the original 750 numbers, 742 were checked, with 242 being ineligible (54 did not have an article assigned, 100 were from before the year 2000, 35 were not in English, and 53 were not in English and before the year 2000). The selected article distribution of PMID numbers (by year) was compared to the overall distribution of PMID numbers by year for English articles. The sample was found to be representative of the overall distribution (χ2 (df = 14), *p* > 0.05). This sample size was chosen because given 500 articles and assuming that about half of them might have empirical data, if no article is found to fulfill the criterion for a transparency indicator, then the 95% confidence interval around that 0% estimate does not exceed 1%.

Two investigators independently characterized and then cross-compared all extractions in groups of 50 articles at a time. Any uncertainties were first discussed in detail, and a third reviewer (JPAI) reassessed articles with arbitration discrepancies.

The sample was characterized into seven study categories: (1) no research (items with no data such as editorials, commentaries, news, comments and non-systematic expert reviews), (2) models/modeling or software or script or methods without empirical data (other than simulations), (3) case report or series (humans only, with or without review of the literature), (4) randomized clinical trials (humans only), (5) systematic reviews and/or meta-analyses (humans only), (6) cost effectiveness or decision analysis (humans only), and (7) other (empirical data that includes uncontrolled study [human], controlled non-randomized study [human], or basic science studies).

InCites Essential Science Indicators (ESI) was used to determine the main scientific field of each article. The journal for each index paper was searched in ESI in order to find the scientific field to which its Highly Cited Papers are ascribed. If a journal had articles ascribed to more than one scientific field, we examined the first five cited journals referenced by the index article. The journal names for these articles were then searched in ESI. If the majority belonged to the same field, this field was used for the index paper. If there was no majority, a field was selected based on the best judgment of the reviewers (JPAI, SAI, and JDW). If a specific journal was not found on ESI, we searched Journal Citation Reports (JCR) and identified the scientific field to which the highest-cited journal in the same JCR category had been ascribed to in ESI.

Publications in scientific fields not directly related to biomedical research (chemistry, physics, computer science, economics and business, engineering, geosciences, material science, mathematics, physics, and space science) were further excluded from analysis. Even though these fields may sometimes have repercussions for biomedicine, their transparency practices may differ systematically, and their evaluation would require a separate, focused effort. Thus, 59/500 articles were excluded.

JCR was used to determine 2013 journal impact factor. No information was recorded for journals without an impact factor for 2013. Availability of free access in PubMed Central was based on assignment of a PCMID (yes/no).

### Assessment of Indicators of Reproducibility and Transparency

#### Indicators

Publications with data and analyses were assessed for publically available full protocols and datasets, patterns of reproducibility (whether the study claimed to be a replication effort, whether subsequent citing papers had tried to replicate the analyses, and whether data were included in systematic reviews and/or meta-analyses), conflicts of interest, and funding. For published items without data and analyses, only statements of conflict and funding were investigated, since protocols, datasets, and reproducibility were not relevant.

#### Protocol availability

We reviewed the eligible papers for any mention of the protocol, possible hyperlink, or reference to the source for available protocol. We did not investigate references made in the text such as “details have been previously published,” since we believe that these published details would not meet our definition of a full protocol. Furthermore, these would invariably be the methods sections of previous papers and not separate protocols. For the studies that have publically available protocols, we reported whether or not the available protocols cover all or part of the presented analyses.

#### Dataset availability

Articles were scrutinized for any mention of access to the datasets that stand behind the analyses presented in the paper. If studies had datasets, we recorded whether the available datasets cover all or part of the presented analyses.

#### Funding

For each eligible paper, we assessed whether any mention of funding was made, and if so, whether funding had been received and whether this was from public and/or private sources.

#### Replication

Abstracts from papers that included data and analyses were examined for statements regarding study novelty or replication. We used the following categories: based on the abstract and/or introduction, the index paper claims that it presents some novel findings; based on its abstract and introduction, the index paper clearly claims that it is a replication effort trying to validate previous knowledge or it is inferred that the index paper is a replication trying to validate previous knowledge; based on the abstract and/or introduction, it claims to be both novel and replicate previous findings; no statement or unclear statement in the abstract and/or introduction about whether the index paper presents a novel finding or replication (or no distinct abstract and introduction exists).

Furthermore, Web of Knowledge (version 5.14) was utilized to identify the number of citations to each of the index papers with data and analyses as of mid-2014. The citing papers of each index paper were examined to identify whether any of them are systematic reviews and/or meta-analyses and/or studies that claim to try to replicate findings from the index paper. The citing papers were screened at the title level, and those that seem potentially relevant were also screened at the abstract, introduction, and full-text level.

## Supporting Information

S1 Dataset SAS dataset. (SAS7BDAT)

S1 Fig Trends in reported funding in scientific publications 2000–2014. (TIF)

S1 Index Article references. (PDF)

S1 Protocol Study protocol. (PDF)

S1 SAS Code SAS data cleaning/formatting and analysis code. (PDF)

S1 Table Random sampling of 750 PMID numbers 10–25 million. (PDF)

S2 Table Sampling of 500 PMID numbers for English articles published between 2000–2014 with PMID numbers 10–25 million. (PDF)

S3 Table Sample articles were categorized into seven different categories based on article characteristics. (PDF)

S4 Table Articles, by PMID, excluded from data analysis based on field of study. (PDF)

## Abbreviations

ESI: Essential Science Indicators
JCR: Journal Citation Reports
NIH: National Institutes of Health
NSF: National Science Foundation
PMCID: PubMed Central reference number
PMID: PubMed Identification
