# Supplementary figures and images for: Reproducible Research Practices and Transparency across the Biomedical Literature
Source: PLoS Biol. 2016 Jan 4;14(1):e1002333. doi: 10.1371/journal.pbio.1002333 (PMC4699702; doi:10.1371/journal.pbio.1002333)

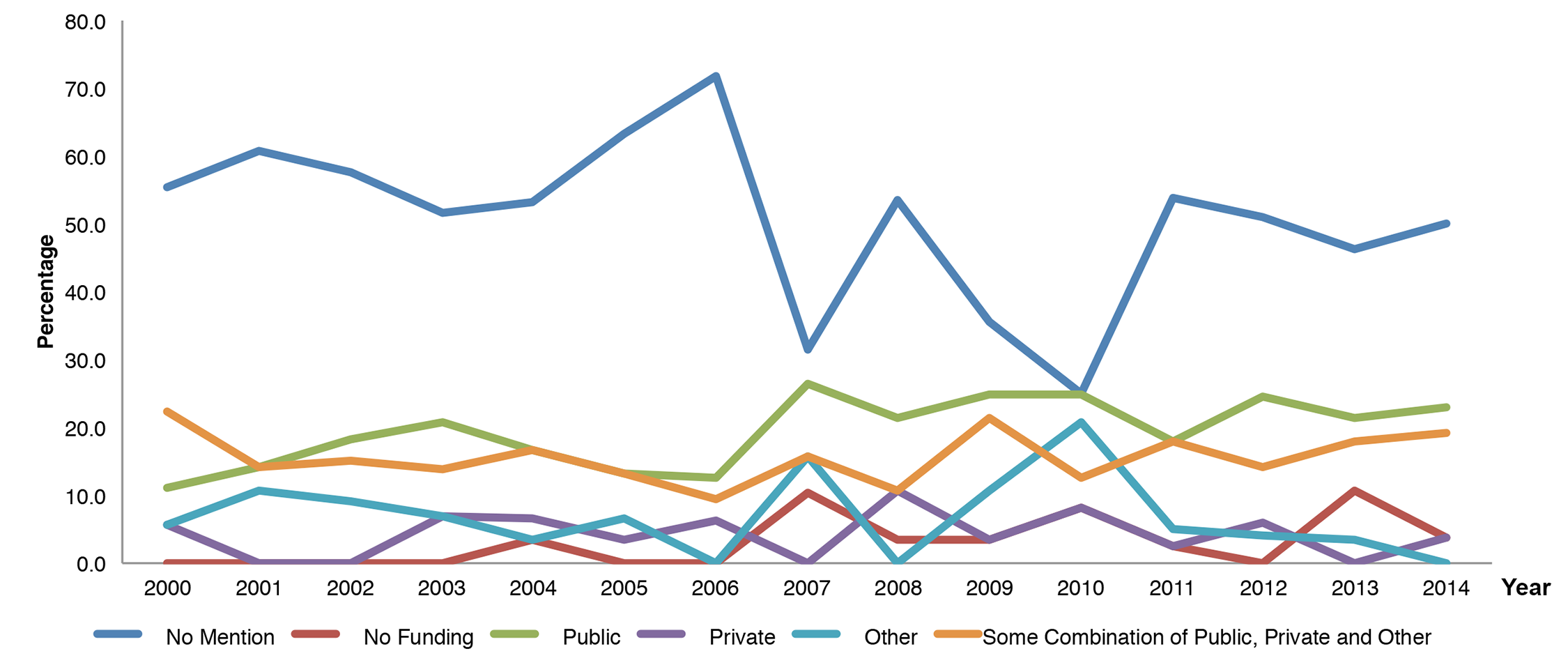

Supplement: S1 Fig — (TIF) [file pbio.1002333.s002.tif]
