# Supplementary material for: Reproducible Research Practices and Transparency across the Biomedical Literature
Source: PLoS Biol. 2016 Jan 4;14(1):e1002333. doi: 10.1371/journal.pbio.1002333 (PMC4699702; doi:10.1371/journal.pbio.1002333)
Supplement: S1 Index — (PDF) [file pbio.1002333.s003.pdf]

A1. Erratum. Trends in cognitive sciences. 2001;5(3):134. PubMed PMID: 11239814.

A2. Health disparities of coal miners and coal mining communities: the role of occupational health nurses. AAOHN journal : official journal of the American Association of Occupational Health Nurses. 2011;59(7):322. doi: 10.3928/08910162-20110624-21. PubMed PMID: 24601975.

A3. Semaphorin 3A and its role in osteoprotection. BoneKEy reports. 2012;1:156. doi: 10.1038/bonekey.2012.156. PubMed PMID: 23951529; PubMed Central PMCID: PMC3727783.

A4. 2014 Scientific Session of the Society of American Gastrointestinal and Endoscopic Surgeons (SAGES), Salt Lake City, Utah, USA, 2-5 April 2014 : Posters of Distinction. Surgical endoscopy. 2014;28 Suppl 1:304-10. doi: 10.1007/s00464-014-3477-y. PubMed PMID: 24609705.

A5. Abdel-Haq NM, Harahsheh A, Asmar BL. Retropharyngeal abscess in children: the emerging role of group A beta hemolytic streptococcus. Southern medical journal. 2006;99(9):927-31. PubMed PMID: 17004526.

A6. Abdo AA. Clinical presentation, response to therapy, and predictors of fibrosis in patients with autoimmune hepatitis in Saudi Arabia. Saudi journal of gastroenterology : official journal of the Saudi Gastroenterology Association. 2006;12(2):73-6. PubMed PMID: 19858589.

A7. Abe H, Kumamoto K, Hoshino M, Utsumi Y, Takenoshita S, Kaneko F. Pericostal tuberculosis diagnosed by fine needle aspiration biopsy: report

of a case with a review of the literature. The Journal of dermatology. 2005;32(1):52-7. PubMed PMID: 15841663.

A8. Agrawal AM, Neau SH, Bonate PL. Wet granulation fine particle ethylcellulose tablets: effect of production variables and mathematical modeling of drug release. AAPS pharmSci. 2003;5(2):E13. doi: 10.1208/ps050213. PubMed PMID: 12866940; PubMed Central PMCID: PMC2751521.

A9. Ahrendt HJ, Adolf D, Buhling KJ. Advantages and challenges of oestrogen-free hormonal contraception. Current medical research and opinion. 2010;26(8):1947-55. doi: 10.1185/03007995.2010.493088. PubMed PMID: 20569066.

A10. Aiken AH, Chang SM, Larson D, Butowski N, Cha S. Longitudinal magnetic resonance imaging features of glioblastoma multiforme treated with radiotherapy with or without brachytherapy. International journal of radiation oncology, biology, physics. 2008;72(5):1340-6. doi: 10.1016/j.ijrobp.2008.02.078. PubMed PMID: 18538496.

A11. Akagi I, Miyashita M, Ishibashi O, Mishima T, Kikuchi K, Makino H, et al. Relationship between altered expression levels of MIR21, MIR143, MIR145, and MIR205 and clinicopathologic features of esophageal squamous cell carcinoma. Diseases of the esophagus : official journal of the International Society for Diseases of the Esophagus / ISDE. 2011;24(7):523-30. doi: 10.1111/j.1442-2050.2011.01177.x. PubMed PMID: 21453382.

A12. Akazawa M, Sindelar JL, Paltiel AD. Economic costs of influenza-related work absenteeism. Value in health : the journal of the International Society for Pharmacoeconomics and Outcomes Research. 2003;6(2):107-15. doi: 10.1046/j.1524-4733.2003.00209.x. PubMed PMID: 12641861.

A13. Akbar B, Niloufar N, Abolfazl M, Lofollah S, Ali KQ, Soheyla V. Evaluation and comparison of zinc absorption level from 2-Alkyle 3-Hydroxy pyranon-zinc complexes and zinc sulfate in rat in vivo. Advanced biomedical research. 2013;2:77. doi: 10.4103/2277-9175.116432. PubMed PMID: 24223392; PubMed Central PMCID: PMC3814584.

A14. Al Quran FA, Al-Dwairi ZN. Torus palatinus and torus mandibularis in edentulous patients. The journal of contemporary dental practice. 2006;7(2):112-9. PubMed PMID: 16685302.

A15. Alava JJ, Calle N. Drilling plans endanger Yasuni's biodiversity. Science. 2013;342(6161):931-2. doi: 10.1126/science.342.6161.931-a. PubMed PMID: 24264977.

A16. Albuquerque AV, Drumond AL, Shao S, Melo RC, Almeida FR, Meistrich ML, et al. A New Approach for Optimal Morphological Identification and Immunolabeling of Spermatogonial Cells. Microscopy and microanalysis : the official journal of Microscopy Society of America, Microbeam Analysis Society, Microscopical Society of Canada. 2014:1-8. doi: 10.1017/S1431927614000889. PubMed PMID: 24834474.

A17. Alvarado G, D'Andrea LA. Why do people snore? Scientific American. 2004;291(1):120. PubMed PMID: 15255597.

A18. Ambroz KL, Zhang Y, Schutz-Geschwender A, Olive DM. Blocking and detection chemistries affect antibody performance on reverse phase protein arrays. Proteomics. 2008;8(12):2379-83. doi: 10.1002/pmic.200700676. PubMed PMID: 18563731.

A19. American Diabetes A. Screening for type 2 diabetes. Diabetes care. 2003;26 Suppl 1:S21-4. PubMed PMID: 12502615.

A20. Androutsos G. Leon Berard (1870-1956): creator of the homonymous anticancer center of Lyon. Journal of BUON : official journal of the Balkan Union of Oncology. 2006;11(2):245-50. PubMed PMID: 17318980.

A21. Angelico M, Gridelli B, Strazzabosco M, Transplantation AISFCoL. Practice of adult liver transplantation in Italy. Recommendations of the Italian Association for the Study of the Liver (A.I.S.F.). Digestive and liver disease : official journal of the Italian Society of Gastroenterology and the Italian Association for the Study of the Liver. 2005;37(7):461-7. doi: 10.1016/j.dld.2005.03.006. PubMed PMID: 15893508.

A22. Antunes FM, Malmierca MS. An Overview of Stimulus-Specific Adaptation in the Auditory Thalamus. Brain topography. 2013. doi: 10.1007/s10548-013-0342-6. PubMed PMID: 24343247.

A23. Aras R. Social marketing in healthcare. The Australasian medical journal. 2011;4(8):418-A24. doi: 10.4066/AMJ.2011.626. PubMed PMID: 23393528; PubMed Central PMCID: PMC3562881.

A24. Arbib MA. From grasp to language: embodied concepts and the challenge of abstraction. Journal of physiology, Paris. 2008;102(1-3):4-20. doi: 10.1016/j.jphysparis.2008.03.001. PubMed PMID: 18440207.

A25. Argaw AT, Gurfein BT, Zhang Y, Zameer A, John GR. VEGF-mediated disruption of endothelial CLN-5 promotes blood-brain barrier breakdown. Proceedings of the National Academy of Sciences of the United States of America. 2009;106(6):1977-82. doi: 10.1073/pnas.0808698106. PubMed PMID: 19174516; PubMed Central PMCID: PMC2644149.

A26. Asano R, Nagami A, Fukumoto Y, Miura K, Yazama F, Ito H, et al. Synthesis and biological evaluation of new boron-containing chlorin derivatives as agents for both photodynamic therapy and boron neutron capture therapy of cancer. Bioorganic & medicinal chemistry letters. 2014;24(5):1339-43. doi: 10.1016/j.bmcl.2014.01.054. PubMed PMID: 24508130.

A27. Astachov L, Nevo Z, Brosh T, Vago R. The structural, compositional and mechanical features of the calcite shell of the barnacle Tetraclita rufotincta. Journal of structural biology. 2011;175(3):311-8. doi: 10.1016/j.jsb.2011.04.014. PubMed PMID: 21549194.

A28. Audelin MC, Savage PD, Ades PA. Changing clinical profile of patients entering cardiac rehabilitation/secondary prevention programs: 1996 to 2006. Journal of cardiopulmonary rehabilitation and prevention. 2008;28(5):299-306. doi: 10.1097/01.HCR.0000336139.48698.26. PubMed PMID: 18784538.

A29. Avendano-Garcia M, Mercado U, Marin ME. A case of Peutz-Jeghers syndrome associated with duodenal carcinoma and sickle cell anemia. The American journal of gastroenterology. 2002;97(3):762-3. doi: 10.1111/j.1572-0241.2002.05569.x. PubMed PMID: 11922580.

A30. Awofala AA, Davies JA, Jones S. Functional roles for redox genes in ethanol sensitivity in Drosophila. Functional & integrative genomics. 2012;12(2):305-15. doi: 10.1007/s10142-012-0272-5. PubMed PMID: 22430022.

A31. Azaroff LS, Levenstein C, Wegman DH. Occupational health of Southeast Asian immigrants in a US city: a comparison of data sources. American journal of public health. 2003;93(4):593-8. PubMed PMID: 12660203; PubMed Central PMCID: PMC1447796.

A32. Azuma T, Takahashi S, Kawamura A. Preoperative autologous blood donation in hip surgeries. Transfusion science. 2000;23(3):177-81. PubMed PMID: 11099892.

A33. Bachelet D, Truong T, Verner MA, Arveux P, Kerbrat P, Charlier C, et al. Determinants of serum concentrations of 1,1-dichloro-2,2-bis(p-chlorophenyl)ethylene and polychlorinated biphenyls among French women in the CECILE study. Environmental research. 2011;111(6):861-70. doi: 10.1016/j.envres.2011.06.001. PubMed PMID: 21684540.

A34. Bacon JL, Patterson CM, Madden BP. Indications and interventional options for non-resectable tracheal stenosis. Journal of thoracic disease.

2014;6(3):258-70. doi: 10.3978/j.issn.2072-1439.2013.11.08. PubMed PMID: 24624290; PubMed Central PMCID: PMC3949180.

A35. Badireddy AR, Budarz JF, Chellam S, Wiesner MR. Bacteriophage inactivation by UV-A illuminated fullerenes: role of nanoparticle-virus association and biological targets. Environmental science & technology. 2012;46(11):5963-70. doi: 10.1021/es300340u. PubMed PMID: 22545948.

A36. Baker JM, Good DE. Physiological emotional under-arousal in individuals with mild head injury. Brain injury : [BI]. 2014;28(1):51-65. doi: 10.3109/02699052.2013.857787. PubMed PMID: 24328800.

A37. Bakker NA, Verschuuren EA, Veeger NJ, van der Bij W, van Imhoff GW, Kallenberg CG, et al. Quantification of Epstein-Barr virus-DNA load in lung transplant recipients: a comparison of plasma versus whole blood. The Journal of heart and lung transplantation : the official publication of the International Society for Heart Transplantation. 2008;27(1):7-10. doi: 10.1016/j.healun.2007.10.008. PubMed PMID: 18187080.

A38. Balasubramanian K, Burghard M, Kern K, Scolari M, Mews A. Photocurrent imaging of charge transport barriers in carbon nanotube devices. Nano letters. 2005;5(3):507-10. doi: 10.1021/nl050053k. PubMed PMID: 15755103.

A39. Baldessarini RJ, Tondo L. Does lithium treatment still work? Evidence of stable responses over three decades. Archives of general psychiatry. 2000;57(2):187-90. PubMed PMID: 10665622.

A40. Ballone E, Fazii P, Lappa G, Di Mascio R, Di Mascio C, Schioppa F. Prevalence of Becker's nevi in a population of young men in central Italy. Journal of the American Academy of Dermatology. 2003;48(5):795. doi: 10.1067/mjd.2003.182. PubMed PMID: 12734514.

A41. Banaschak S, Michael M. Detection of fetal DNA in a cell pellet after centrifugation of mountant. Journal of forensic sciences. 2003;48(1):135-6. PubMed PMID: 12570214.

A42. Baran PS, Maimone TJ. Organic chemistry: a tuxedo for iodine atoms. Nature. 2007;445(7130):826-7. doi: 10.1038/445826a. PubMed PMID: 17314962.

A43. Barnett WS. Effectiveness of early educational intervention. Science. 2011;333(6045):975-8. doi: 10.1126/science.1204534. PubMed PMID: 21852490.

A44. Bartlett A, King M, Phillips P. Straight talking: an investigation of the attitudes and practice of psychoanalysts and psychotherapists in relation to gays and lesbians. The British journal of psychiatry : the journal of mental science. 2001;179:545-9. PubMed PMID: 11731360.

A45. Bax M. Does 'therapy' have a future? Developmental medicine and child neurology. 2001;43(1):3. PubMed PMID: 11201420.

A46. Begley DJ. Delivery of therapeutic agents to the central nervous system: the problems and the possibilities. Pharmacology & therapeutics. 2004;104(1):29-45. doi: 10.1016/j.pharmthera.2004.08.001. PubMed PMID: 15500907.

A47. Beis LY, Polyviou T, Malkova D, Pitsiladis YP. The effects of creatine and glycerol hyperhydration on running economy in well trained endurance runners. Journal of the International Society of Sports Nutrition. 2011;8(1):24. doi: 10.1186/1550-2783-8-24. PubMed PMID: 22176668; PubMed Central PMCID: PMC3283512.

A48. Beisse R. Endoscopic treatment of spinal trauma at the thoracolumbar junction. Indian journal of orthopaedics. 2007;41(4):277-85. doi: 10.4103/0019-5413.36987. PubMed PMID: 21139778; PubMed Central PMCID: PMC2989503.

A49. Belo I, Pinheiro R, Mota M. Morphological and physiological changes in Saccharomyces cerevisiae by oxidative stress from hyperbaric air. Journal of biotechnology. 2005;115(4):397-404. doi: 10.1016/j.jbiotec.2004.09.010. PubMed PMID: 15639101.

A50. Berger KI, Ayappa I, Chatr-Amontri B, Marfatia A, Sorkin IB, Rapoport DM, et al. Obesity hypoventilation syndrome as a spectrum of respiratory disturbances during sleep. Chest. 2001;120(4):1231-8. PubMed PMID: 11591566.

A51. Berlin J, Tutsch KD, Arzoomanian RZ, Alberti D, Binger K, Feierabend C, et al. Phase I and pharmacokinetic study of a micronized formulation of carboxyamidotriazole, a calcium signal transduction inhibitor: toxicity, bioavailability and the effect of food. Clinical cancer research : an official journal of the American Association for Cancer Research. 2002;8(1):86-94. PubMed PMID: 11801543.

A52. Bhatia S, Frank RM, Ghodadra NS, Hsu AR, Romeo AA, Bach BR, Jr., et al. The outcomes and surgical techniques of the latarjet procedure. Arthroscopy : the journal of arthroscopic & related surgery : official publication of the Arthroscopy Association of North America and the International Arthroscopy Association. 2014;30(2):227-35. doi: 10.1016/j.arthro.2013.10.013. PubMed PMID: 24485116.

A53. Bhattacharya A, Akhter S, Shahnawaz S, Siddiqui AW, Ahmad MZ. Evaluation of Assam Bora rice starch as plasma volume expander by polymer analysis. Current drug delivery. 2010;7(5):436-41. PubMed PMID: 20950261.

A54. Birnbaum EH. What's new in colon and rectal surgery. Journal of the American College of Surgeons. 2006;202(3):485-94. doi: 10.1016/j.jamcollsurg.2005.10.007. PubMed PMID: 16500254.

A55. Biron-Shental T, Fishman A, Fejgin MD. Medical and mechanical methods for cervical ripening. International journal of gynaecology and obstetrics: the official organ of the International Federation of Gynaecology and Obstetrics. 2004;85(2):159-60. doi: 10.1016/j.ijgo.2003.08.006. PubMed PMID: 15099778.

A56. Bisgaard H. Leukotriene modifiers in pediatric asthma management. Pediatrics. 2001;107(2):381-90. PubMed PMID: 11158473.

A57. Bishop SC, Lerch M, McCord BR. Detection of nitrated benzodiazepines by indirect laser-induced fluorescence detection on a microfluidic device.

Journal of chromatography A. 2007;1154(1-2):481-4. doi: 10.1016/j.chroma.2007.05.004. PubMed PMID: 17499754.

A58. Bjornland T, Larheim TA. Discectomy of the temporomandibular joint: 3-year follow-up as a predictor of the 10-year outcome. Journal of oral and maxillofacial surgery : official journal of the American Association of Oral and Maxillofacial Surgeons. 2003;61(1):55-60. doi: 10.1053/joms.2003.50010. PubMed PMID: 12524609.

A59. Bliwise DL, Kutner NG, Zhang R, Parker KP. Survival by time of day of hemodialysis in an elderly cohort. JAMA : the journal of the American Medical Association. 2001;286(21):2690-4. PubMed PMID: 11730444.

A60. Blumenthal T. Split genes: another surprise from Giardia. Current biology : CB. 2011;21(4):R162-3. doi: 10.1016/j.cub.2011.01.032. PubMed PMID: 21334298.

A61. Bogomolny E, Giraud O. Perturbation approach to multifractal dimensions for certain critical random-matrix ensembles. Physical review E, Statistical, nonlinear, and soft matter physics. 2011;84(3 Pt 2):036212. PubMed PMID: 22060480.

A62. Bolognesi MP, Pietrobon R, Clifford PE, Vail TP. Comparison of a hydroxyapatite-coated sleeve and a porous-coated sleeve with a modular revision hip stem. A prospective, randomized study. The Journal of bone and joint surgery American volume. 2004;86-A(12):2720-5. PubMed PMID: 15590859.

A63. Bolon B, Campagnuolo G, Zhu L, Duryea D, Zack D, Feige U. Interleukin-1beta and tumor necrosis factor-alpha produce distinct, time-dependent patterns of acute arthritis in the rat knee. Veterinary pathology. 2004;41(3):235-43. doi: 10.1354/vp.41-3-235. PubMed PMID: 15133172.

A64. Borgstein J. The chief's broken leg. Lancet. 2002;359(9310):988. doi: 10.1016/S0140-6736(02)08041-8. PubMed PMID: 11918964.

A65. Bosy-Westphal A, Kossel E, Goele K, Later W, Hitze B, Settler U, et al. Contribution of individual organ mass loss to weight loss-associated decline in resting energy expenditure. The American journal of clinical nutrition. 2009;90(4):993-1001. doi: 10.3945/ajcn.2008.27402. PubMed PMID: 19710198.

A66. Boullerne AI, Rodriguez JJ, Touil T, Brochet B, Schmidt S, Abrous ND, et al. Anti-S-nitrosocysteine antibodies are a predictive marker for demyelination in experimental autoimmune encephalomyelitis: implications for multiple sclerosis. The Journal of neuroscience : the official journal of the Society for Neuroscience. 2002;22(1):123-32. PubMed PMID: 11756495.

A67. Boulouis G, Dumont F, Cordonnier C, Bodenant M, Leys D, Henon H. Intravenous thrombolysis for acute cerebral ischaemia in old stroke patients >/= 80 years of age. Journal of neurology. 2012;259(7):1461-7. doi: 10.1007/s00415-011-6359-4. PubMed PMID: 22183776.

A68. Brasile L, Stubenitsky BM, Booster MH, Lindell S, Araneda D, Buck C, et al. Overcoming severe renal ischemia: the role of ex vivo warm perfusion. Transplantation. 2002;73(6):897-901. PubMed PMID: 11923688.

A69. Breier JI, Papanicolaou AC. Spatiotemporal patterns of brain activation during an action naming task using magnetoencephalography. Journal of clinical neurophysiology : official publication of the American Electroencephalographic Society. 2008;25(1):7-12. doi: 10.1097/WNP.0b013e318163ccd5. PubMed PMID: 18303555; PubMed Central PMCID: PMC3086561.

A70. Brick AV. The medical education and the Unified Health System. Revista brasileira de cirurgia cardiovascular : orgao oficial da Sociedade Brasileira de Cirurgia Cardiovascular. 2012;27(2):331-3. PubMed PMID: 22996987.

A71. Brindle S, Douglas F, van Teijlingen E, Vanora H. Midwifery research: questionnaire surveys. RCM midwives : the official journal of the Royal College of Midwives. 2005;8(4):156-8. PubMed PMID: 15839136.

A72. Broer DJ. Liquid crystals: Defects dictated. Nature materials. 2010;9(2):99-100. doi: 10.1038/nmat2617. PubMed PMID: 20094080.

A73. Brown JK, Knight PA, Wright SH, Thornton EM, Miller HR. Constitutive secretion of the granule chymase mouse mast cell protease-1 and the chemokine, CCL2, by mucosal mast cell homologues. Clinical and experimental allergy : journal of the British Society for Allergy and Clinical Immunology. 2003;33(1):132-46. PubMed PMID: 12534561.

A74. Brusca I, Li Vigni P, Sucato R, Cilluffo N, La Chiusa SM. Evaluation of antibodies to thymine ROS-modified DNA poly(dT), in patients with immunologic disorders: relationships to anti n-DNA and anti ENA autoantibodies. Panminerva medica. 2002;44(1):33-5. PubMed PMID: 11887089.

A75. Butler FK, Gurney N. Orbital hemorrhage following face-mask barotrauma. Undersea & hyperbaric medicine : journal of the Undersea and Hyperbaric Medical Society, Inc. 2001;28(1):31-4. PubMed PMID: 11732882.

A76. Campos D, Mendez V. Reaction-diffusion wave fronts on comblike structures. Physical review E, Statistical, nonlinear, and soft matter physics. 2005;71(5 Pt 1):051104. PubMed PMID: 16089518.

A77. Carlson GA, Klein DN. How to understand divergent views on bipolar disorder in youth. Annual review of clinical psychology. 2014;10:529-51. doi: 10.1146/annurev-clinpsy-032813-153702. PubMed PMID: 24387237.

A78. Carroll J. Keeping ED costs down an exercise in frustration. Managed care. 2012;21(1):44-6. PubMed PMID: 22334939.

A79. Carter S, Le JD, Hu JC. Anatomic and technical considerations for optimizing recovery of sexual function during robotic-assisted radical prostatectomy. Current opinion in urology. 2013;23(1):88-94. doi: 10.1097/MOU.0b013e32835b6602. PubMed PMID: 23169152.

A80. Casanova M, Ferrari A, Bisogno G, Merks JH, De Salvo GL, Meazza C, et al. Vinorelbine and low-dose cyclophosphamide in the treatment of pediatric sarcomas: pilot study for the upcoming European

Rhabdomyosarcoma Protocol. Cancer. 2004;101(7):1664-71. doi: 10.1002/cncr.20544. PubMed PMID: 15378498.

A81. Cavone L, Chiarugi A. Targeting poly(ADP-ribose) polymerase-1 as a promising approach for immunomodulation in multiple sclerosis? Trends in molecular medicine. 2012;18(2):92-100. doi: 10.1016/j.molmed.2011.10.002. PubMed PMID: 22078487.

A82. Chaigne-Delalande B, Anies G, Kramer I, Genot E. Nonadherent cells switch to a Rac-mediated, SHIP regulated, Akt activation mode for survival. Oncogene. 2008;27(13):1876-85. doi: 10.1038/sj.onc.1210830. PubMed PMID: 17906692.

A83. Chan DY, Fong KN. The effects of problem-solving skills training based on metacognitive principles for children with acquired brain injury attending mainstream schools: a controlled clinical trial. Disability and rehabilitation. 2011;33(21-22):2023-32. doi: 10.3109/09638288.2011.556207. PubMed PMID: 21345132.

A84. Chan I, Bicknell SG, Graham M. Utility and diagnostic accuracy of sonography in detecting appendicitis in a community hospital. AJR American journal of roentgenology. 2005;184(6):1809-12. doi: 10.2214/ajr.184.6.01841809. PubMed PMID: 15908535.

A85. Chang CA, Wu BH, Kuan BY. Macrocyclic lanthanide complexes as artificial nucleases and ribonucleases: effects of pH, metal ionic radii, number of coordinated water molecules, charge, and concentrations of the

metal complexes. Inorganic chemistry. 2005;44(19):6646-54. doi: 10.1021/ic0485458. PubMed PMID: 16156622.

A86. Chase LE, Ely LO, Hutjens MF. Major advances in extension education programs in dairy production. Journal of dairy science. 2006;89(4):1147-54. doi: 10.3168/jds.S0022-0302(06)72183-X. PubMed PMID: 16537947.

A87. Chen CL, Chung CY, Cheng PT, Chen CH, Chen MH. Linguistic and gait disturbance in a child with Laurence-Moon-Biedl syndrome: left temporal and parietal lobe hypoplasia. American journal of physical medicine & rehabilitation / Association of Academic Physiatrists. 2004;83(1):69-74. doi: 10.1097/01.PHM.0000091989.01773.1C. PubMed PMID: 14709978.

A88. Chen CT, Chen YR. Update on orbital reconstruction. Current opinion in otolaryngology & head and neck surgery. 2010;18(4):311-6. doi: 10.1097/MOO.0b013e32833aafd2. PubMed PMID: 20631536.

A89. Chen ML, Chang HK. Physical symptom profiles of depressed and nondepressed patients with cancer. Palliative medicine. 2004;18(8):712-8. PubMed PMID: 15623168.

A90. Chien CY, Chang YJ, Chang JE, Lee MS, Chen WY, Hsu TM, et al. Formation of Ge quantum dots array in layer-cake technique for advanced photovoltaics. Nanotechnology. 2010;21(50):505201. doi: 10.1088/0957-4484/21/50/505201. PubMed PMID: 21098937.

A91. Chiu CP, Lairson LL, Gilbert M, Wakarchuk WW, Withers SG, Strynadka NC. Structural analysis of the alpha-2,3-sialyltransferase Cst-I from Campylobacter jejuni in apo and substrate-analogue bound forms.

Biochemistry. 2007;46(24):7196-204. doi: 10.1021/bi602543d. PubMed PMID: 17518445.

A92. Cho SH, Godin J, Lo YH. Optofluidic Waveguides in Teflon AF-Coated PDMS Microfluidic Channels. IEEE photonics technology letters : a publication of the IEEE Laser and Electro-optics Society. 2009;21(15):1057-9. doi: 10.1109/LPT.2009.2022276. PubMed PMID: 20729984; PubMed Central PMCID: PMC2923848.

A93. Choi SJ, Kim JS, Kim JH, Oh SJ, Lee JG, Kim CJ, et al. [18F]3'-deoxy-3'-fluorothymidine PET for the diagnosis and grading of brain tumors. European journal of nuclear medicine and molecular imaging. 2005;32(6):653-9. doi: 10.1007/s00259-004-1742-3. PubMed PMID: 15711980.

A94. Choukroun J, Diss A, Simonpieri A, Girard MO, Schoeffler C, Dohan SL, et al. Platelet-rich fibrin (PRF): a second-generation platelet concentrate. Part IV: clinical effects on tissue healing. Oral surgery, oral medicine, oral pathology, oral radiology, and endodontics. 2006;101(3):e56-60. doi: 10.1016/j.tripleo.2005.07.011. PubMed PMID: 16504852.

A95. Chow SL, Chandran V, Fazelzad R, Johnson SR. Prognostic factors for survival in systemic lupus erythematosus associated pulmonary hypertension. Lupus. 2012;21(4):353-64. doi: 10.1177/0961203311429815. PubMed PMID: 22127457.

A96. Chuang MH, Wang YF, Chen M, Cham TM. Effectiveness of implementation of a new drug storage label and error-reducing process on

the accuracy of drug dispensing. Journal of medical systems. 2012;36(3):1469-74. doi: 10.1007/s10916-010-9607-8. PubMed PMID: 20953679.

A97. Clarke G. Recording wounds: Polaroid's new medically-designed cameras. British journal of community nursing. 2000;5(11):578-80. PubMed PMID: 12066058.

A98. Cleland JC, Logigian EL, Thaisetthawatkul P, Herrmann DN. Dispersion of the distal compound muscle action potential in chronic inflammatory demyelinating polyneuropathy and carpal tunnel syndrome. Muscle & nerve. 2003;28(2):189-93. doi: 10.1002/mus.10420. PubMed PMID: 12872323.

A99. Clezardin P, Benzaid I, Croucher PI. Bisphosphonates in preclinical bone oncology. Bone. 2011;49(1):66-70. doi: 10.1016/j.bone.2010.11.017. PubMed PMID: 21145441.

A100. Coelho da Mota DS, Furtado E, Bottino DA, Bouskela E. Effects of buflomedil and pentoxifylline on hamster skin-flap microcirculation: prediction of flap viability using orthogonal polarization spectral imaging. Clinics. 2009;64(8):797-802. doi: 10.1590/S1807-59322009000800015. PubMed PMID: 19690666; PubMed Central PMCID: PMC2728195.

A101. Collinge C. An injectable BMP cocktail for fracture-healing--a study that others have only imagined: commentary on an article by Thomas Lyon, MD, et al.: "Efficacy and safety of recombinant human bone morphogenetic protein-2/calcium phosphate matrix for closed tibial

diaphyseal fracture. a double-blind, randomized, controlled phase-II/III trial". The Journal of bone and joint surgery American volume. 2013;95(23):e1891-2. doi: 10.2106/JBJS.M.01179. PubMed PMID: 24306712.

A102. Columbo LL, Rizza C, Brambilla M, Prati F, Tissoni G. A concomitant and complete set of nonvolatile all-optical logic gates based on hybrid spatial solitons. Optics express. 2014;22(6):6934-47. doi: 10.1364/OE.22.006934. PubMed PMID: 24664042.

A103. Cook K, Atcherson SR. Impulse noise: can hitting a softball harm your hearing? TheScientificWorldJournal. 2014;2014:702723. doi: 10.1155/2014/702723. PubMed PMID: 24778596; PubMed Central PMCID: PMC3981188.

A104. Cooper HJ, Ranawat AS, Potter HG, Foo LF, Koob TW, Ranawat CS. Early reactive synovitis and osteolysis after total hip arthroplasty. Clinical orthopaedics and related research. 2010;468(12):3278-85. doi: 10.1007/s11999-010-1361-1. PubMed PMID: 20419484; PubMed Central PMCID: PMC2974890.

A105. Cossu G, Boria S, Copioli C, Bracceschi R, Giuberti V, Santelli E, et al. Motor representation of actions in children with autism. PloS one. 2012;7(9):e44779. doi: 10.1371/journal.pone.0044779. PubMed PMID: 22970304; PubMed Central PMCID: PMC3438166.

A106. Costenbader KH, Prescott J, Zee RY, De Vivo I. Immunosenescence and rheumatoid arthritis: does telomere shortening predict impending disease?

Autoimmunity reviews. 2011;10(9):569-73. doi: 10.1016/j.autrev.2011.04.034. PubMed PMID: 21575746; PubMed Central PMCID: PMC3119949.

A107. Crawford NR, Hee AG, Long JR. Cluster synthesis via ligand-arrested solid growth: triethylphosphine-capped fragments of binary metal chalcogenides. Journal of the American Chemical Society. 2002;124(50):14842-3. PubMed PMID: 12475314.

A108. Crehuet R, Field MJ. Comment on "Action-derived molecular dynamics in the study of rare events". Physical review letters. 2003;90(8):089801; author reply 2. PubMed PMID: 12633474.

A109. Crescentini M, Bennati M, Carminati M, Tartagni M. Noise Limits of CMOS Current Interfaces for Biosensors: A Review. IEEE transactions on biomedical circuits and systems. 2014;8(2):278-92. doi: 10.1109/TBCAS.2013.2262998. PubMed PMID: 24875287.

A110. Croft M. Co-stimulatory members of the TNFR family: keys to effective T-cell immunity? Nature reviews Immunology. 2003;3(8):609-20. doi: 10.1038/nri1148. PubMed PMID: 12974476.

A111. Cubaud T, Sauzade M, Sun R. CO(2) dissolution in water using long serpentine microchannels. Biomicrofluidics. 2012;6(2):22002-220029. doi: 10.1063/1.3693591. PubMed PMID: 22655006; PubMed Central PMCID: PMC3360710.

A112. Cui X, McGrath JJ, Burne TH, Mackay-Sim A, Eyles DW. Maternal vitamin D depletion alters neurogenesis in the developing rat brain. International

journal of developmental neuroscience : the official journal of the International Society for Developmental Neuroscience. 2007;25(4):227-32. doi: 10.1016/j.ijdevneu.2007.03.006. PubMed PMID: 17467223.

A113. Cunningham AP, Love WK, Zhang RW, Andrews LG, Tollefsbol TO. Telomerase inhibition in cancer therapeutics: molecular-based approaches. Current medicinal chemistry. 2006;13(24):2875-88. PubMed PMID: 17073634; PubMed Central PMCID: PMC2423208.

A114. Cuzick J. Forest plots and the interpretation of subgroups. Lancet. 2005;365(9467):1308. doi: 10.1016/S0140-6736(05)61026-4. PubMed PMID: 15823379.

A115. Danyi S, Widart J, Douny C, Dang PK, Baiwir D, Wang N, et al. Determination and kinetics of enrofloxacin and ciprofloxacin in Tra catfish (Pangasianodon hypophthalmus) and giant freshwater prawn (Macrobrachium rosenbergii) using a liquid chromatography/mass spectrometry method. Journal of veterinary pharmacology and therapeutics. 2011;34(2):142-52. doi: 10.1111/j.1365-2885.2010.01204.x. PubMed PMID: 21395605.

A116. Dao Nguyen X, Robinson DS. Fluticasone propionate increases CD4CD25 T regulatory cell suppression of allergen-stimulated CD4CD25 T cells by an IL-10-dependent mechanism. The Journal of allergy and clinical immunology. 2004;114(2):296-301. doi: 10.1016/j.jaci.2004.04.048. PubMed PMID: 15316506.

A117. Datta S, Li J, Mahdi F, Jekabsons MB, Nagle DG, Zhou YD. Glycolysis inhibitor screening identifies the bis-geranylacylphloroglucinol protonophore moronone from Moronobea coccinea. Journal of natural products. 2012;75(12):2216-22. doi: 10.1021/np300711e. PubMed PMID: 23245650; PubMed Central PMCID: PMC3532528.

A118. Davidson MJ, White JK, Baim DS. Percutaneous therapies for valvular heart disease. Cardiovascular pathology : the official journal of the Society for Cardiovascular Pathology. 2006;15(3):123-9. doi: 10.1016/j.carpath.2006.02.004. PubMed PMID: 16697924.

A119. de Brito Galvao JF, Chew DJ. Metabolic complications of endocrine surgery in companion animals. The Veterinary clinics of North America Small animal practice. 2011;41(5):847-68, v. doi: 10.1016/j.cvsm.2011.05.012. PubMed PMID: 21889689.

A120. De Santi L, Annunziata P. Symptomatic cranial neuralgias in multiple sclerosis: clinical features and treatment. Clinical neurology and neurosurgery. 2012;114(2):101-7. doi: 10.1016/j.clineuro.2011.10.044. PubMed PMID: 22130044.

A121. de Souza PL, Liauw W, Links M, Pirabhahar S, Kelly G, Howes LG. Phase I and pharmacokinetic study of weekly NV06 (Phenoxodiol), a novel isoflav-3-ene, in patients with advanced cancer. Cancer chemotherapy and pharmacology. 2006;58(4):427-33. doi: 10.1007/s00280-006-0189-6. PubMed PMID: 16463060.

A122. De Wals P, Nguyen VH, Erickson LJ, Guay M, Drapeau J, St-Laurent J. Cost-effectiveness of immunization strategies for the control of serogroup C meningococcal disease. Vaccine. 2004;22(9-10):1233-40. doi: 10.1016/j.vaccine.2003.09.022. PubMed PMID: 15003652.

A123. DeJohn P. MedAssets lures IDNs, GPOs with buying clout. Hospital material[dollar sign] management. 2002;27(5):1, 9. PubMed PMID: 12038089.

A124. Dellve L, Cernerud L, Hallberg LR. Harmonizing dilemmas. Siblings of children with DAMP and Asperger syndrome's experiences of coping with their life situations. Scandinavian journal of caring sciences. 2000;14(3):172-8. PubMed PMID: 12035268.

A125. Dieckmann KP, Lauke H, Michl U, Winter E, Loy V. Testicular germ cell cancer despite previous local radiotherapy to the testis. European urology. 2002;41(6):643-9; discussion 9-50. PubMed PMID: 12074782.

A126. Dobrzynska I, Szachowicz-Petelska B, Skrzydlewska E, Figaszewski ZA. Changes in electric charge and phospholipids composition in erythrocyte membrane of ethanol--poisoned rats after administration of teas. Acta poloniae pharmaceutica. 2004;61(6):483-7. PubMed PMID: 15794343.

A127. Dohan DM, Choukroun J, Diss A, Dohan SL, Dohan AJ, Mouhyi J, et al. Platelet-rich fibrin (PRF): a second-generation platelet concentrate. Part III: leucocyte activation: a new feature for platelet concentrates? Oral surgery, oral medicine, oral pathology, oral radiology, and endodontics.

2006;101(3):e51-5. doi: 10.1016/j.tripleo.2005.07.010. PubMed PMID: 16504851.

A128. Dong Z, Yang B, Jin J, Li J, Kang H, Zhong X, et al. Quinoline Group Modified Carbon Nanotubes for the Detection of Zinc Ions. Nanoscale research letters. 2009;4(4):335-40. doi: 10.1007/s11671-008-9248-8. PubMed PMID: 20596287; PubMed Central PMCID: PMC2894356.

A129. Duffy A, Alda M, Hajek T, Grof P. Early course of bipolar disorder in high-risk offspring: prospective study. The British journal of psychiatry : the journal of mental science. 2009;195(5):457-8. doi: 10.1192/bjp.bp.108.062810. PubMed PMID: 19880938.

A130. Dvorakova M, Nencka R, Dejmek M, Zbornikova E, Brezinova A, Pribylova M, et al. Synthesis of alkylcarbonate analogs of O-acetyl-ADP-ribose. Organic & biomolecular chemistry. 2013;11(34):5702-13. doi: 10.1039/c3ob41016a. PubMed PMID: 23884430.

A131. Dyce J, Olmstead ML. Removal of infected canine cemented total hip prostheses using a femoral window technique. Veterinary surgery : VS. 2002;31(6):552-60. PubMed PMID: 12415524.

A132. Eagar P, Hull ML, Howell SM. A method for quantifying the anterior load-displacement behavior of the human knee in both the low and high stiffness regions. Journal of biomechanics. 2001;34(12):1655-60. PubMed PMID: 11716869.

A133. El Demellawy D, Herath C, Khalil M, Nasr A, Moussa B. Fibromatosis of the male breast with concurrent florid-type gynecomastia: Report of a case

and review of literature. Pathology, research and practice. 2011;207(5):306-9. doi: 10.1016/j.prp.2010.11.005. PubMed PMID: 21511401.

A134. Elagili F, Abdullah N, Fong L, Pei T. Aspiration of breast abscess under ultrasound guidance: outcome obtained and factors affecting success. Asian journal of surgery / Asian Surgical Association. 2007;30(1):40-4. doi: 10.1016/S1015-9584(09)60126-3. PubMed PMID: 17337370.

A135. Elias ER, Hansen RM, Irons M, Quinn NB, Fulton AB. Rod photoreceptor responses in children with Smith-Lemli-Opitz syndrome. Archives of ophthalmology. 2003;121(12):1738-43. doi: 10.1001/archopht.121.12.1738. PubMed PMID: 14662594.

A136. Estall JL, Drucker DJ. Dual regulation of cell proliferation and survival via activation of glucagon-like peptide-2 receptor signaling. The Journal of nutrition. 2003;133(11):3708-11. PubMed PMID: 14608100.

A137. Esteban J, Martin-de-Hijas NZ, Ortiz A, Kinnari TJ, Bodas Sanchez A, Gadea I, et al. Detection of lfrA and tap efflux pump genes among clinical isolates of non-pigmented rapidly growing mycobacteria. International journal of antimicrobial agents. 2009;34(5):454-6. doi: 10.1016/j.ijantimicag.2009.06.026. PubMed PMID: 19665358.

A138. Fairman J. A historian's look at the 2012 IOM report on the future of nursing. MD advisor : a journal for New Jersey medical community. 2012;5(4):20-5. PubMed PMID: 23132104.

A139. Farias-Silva E, dos Santos IN, Corezola do Amaral ME, Grassi-Kassisse DM, Spadari-Bratfisch RC. Glucocorticoid receptor and Beta-adrenoceptor expression in epididymal adipose tissue from stressed rats. Annals of the New York Academy of Sciences. 2004;1018:328-32. doi: 10.1196/annals.1296.040. PubMed PMID: 15240386.

A140. Farmer AD, Collings AF, Jameson GJ. The application of power ultrasound to the surface cleaning of silica and heavy mineral sands. Ultrasonics sonochemistry. 2000;7(4):243-7. PubMed PMID: 11062883.

A141. Feldsine PT, Montgomery-Fullerton M, Roa N, Kaur M, Kerr DE, Lienau AH, et al. Comparative validation study to demonstrate the equivalence of a minor modification to AOAC Official Method 2005.05 Assurance GDS shiga Toxin Genes (O157) method to the reference culture method: 375 gram sample size. Journal of AOAC International. 2013;96(4):781-5. PubMed PMID: 24000752.

A142. Feng HT, Wong N, Wee S, Lee MM. Simultaneous determination of 19 intracellular nucleotides and nucleotide sugars in Chinese Hamster ovary cells by capillary electrophoresis. Journal of chromatography B, Analytical technologies in the biomedical and life sciences. 2008;870(1):131-4. doi: 10.1016/j.jchromb.2008.05.038. PubMed PMID: 18541463.

A143. Feng J, Guo H, Li S, Lu T. A study of the mechanism of the chaperone-like function of an scFv of human creatine kinase by computer simulation. PloS one. 2013;8(4):e62147. doi: 10.1371/journal.pone.0062147. PubMed PMID: 23637984; PubMed Central PMCID: PMC3634753.

A144. Fernandez NV, Mestre MC, Marchelli P, Fontenla SB. Yeast and yeast-like fungi associated with dry indehiscent fruits of Nothofagus nervosa in Patagonia, Argentina. FEMS microbiology ecology. 2012;80(1):179-92. doi: 10.1111/j.1574-6941.2011.01287.x. PubMed PMID: 22224476.

A145. Fiederling J, Ulrich CM, Hemminki K, Haug U. Consideration of family history of cancer in medical routine: a survey in the primary care setting in Germany. European journal of cancer prevention : the official journal of the European Cancer Prevention Organisation. 2014;23(3):199-205. doi: 10.1097/CEJ.0b013e328364743d. PubMed PMID: 23880939.

A146. Fiuzat M, Wojdyla D, Kitzman D, Fleg J, Keteyian SJ, Kraus WE, et al. Relationship of beta-blocker dose with outcomes in ambulatory heart failure patients with systolic dysfunction: results from the HF-ACTION (Heart Failure: A Controlled Trial Investigating Outcomes of Exercise Training) trial. Journal of the American College of Cardiology. 2012;60(3):208-15. doi: 10.1016/j.jacc.2012.03.023. PubMed PMID: 22560018; PubMed Central PMCID: PMC3396733.

A147. Fogliano V, Vitaglione P. Functional foods: planning and development. Molecular nutrition & food research. 2005;49(3):256-62. doi: 10.1002/mnfr.200400067. PubMed PMID: 15744710.

A148. Fontanesi JM, Flesher DS, Jr., De Guire M, Lieberthal A, Holcomb K. The cost of doing business: cost structure of electronic immunization registries. Health services research. 2002;37(5):1291-307. PubMed PMID: 12479497; PubMed Central PMCID: PMC1464026.

A149. Frandoloso R, Martinez-Martinez S, Gutierrez-Martin CB, Rodriguez-Ferri EF. Haemophilus parasuis serovar 5 Nagasaki strain adheres and invades PK-15 cells. Veterinary microbiology. 2012;154(3-4):347-52. doi: 10.1016/j.vetmic.2011.07.022. PubMed PMID: 21839589.

A150. Freuler F, Stettler T, Meyerhofer M, Leder L, Mayr LM. Development of a novel Gateway-based vector system for efficient, multiparallel protein expression in Escherichia coli. Protein expression and purification. 2008;59(2):232-41. doi: 10.1016/j.pep.2008.02.003. PubMed PMID: 18375142.

A151. Fuhrs H, Behrens C, Gallien S, Heintz D, Van Dorsselaer A, Braun HP, et al. Physiological and proteomic characterization of manganese sensitivity and tolerance in rice (Oryza sativa) in comparison with barley (Hordeum vulgare). Annals of botany. 2010;105(7):1129-40. doi: 10.1093/aob/mcq046. PubMed PMID: 20237113; PubMed Central PMCID: PMC2887067.

A152. Gao Y, Miao C, Wang Y, Xia J, Zhou P. Metal-resistant microorganisms and metal chelators synergistically enhance the phytoremediation efficiency of Solanum nigrum L. in Cd- and Pb-contaminated soil. Environmental technology. 2012;33(10-12):1383-9. PubMed PMID: 22856313.

A153. Gardiner J, Barton D, Overall R, Marc J. Neurotrophic support and oxidative stress: converging effects in the normal and diseased nervous system. The Neuroscientist : a review journal bringing neurobiology,

neurology and psychiatry. 2009;15(1):47-61. doi: 10.1177/1073858408325269. PubMed PMID: 19218230.

A154. Gaudiano MC, Antoniella E, Bertocchi P, Valvo L. Development and validation of a reversed-phase LC method for analysing potentially counterfeit antimalarial medicines. Journal of pharmaceutical and biomedical analysis. 2006;42(1):132-5. doi: 10.1016/j.jpba.2006.01.059. PubMed PMID: 16517111.

A155. Geocadin RG, Koenig MA. Neurological consultation in the ICU. Seminars in neurology. 2008;28(5):601-2. doi: 10.1055/s-0028-1105980. PubMed PMID: 19115167.

A156. Germa F. The bridge-builders. Canadian family physician Medecin de famille canadien. 2009;55(5):512-3. PubMed PMID: 19439710; PubMed Central PMCID: PMC2682314.

A157. Gerritsen L, Tendolkar I, Franke B, Vasquez AA, Kooijman S, Buitelaar J, et al. BDNF Val66Met genotype modulates the effect of childhood adversity on subgenual anterior cingulate cortex volume in healthy subjects. Molecular psychiatry. 2012;17(6):597-603. doi: 10.1038/mp.2011.51. PubMed PMID: 21577214.

A158. Gershon MD. Developmental determinants of the independence and complexity of the enteric nervous system. Trends in neurosciences. 2010;33(10):446-56. doi: 10.1016/j.tins.2010.06.002. PubMed PMID: 20633936.

A159. Gervasi MG, Marczylo TH, Lam PM, Rana S, Franchi AM, Konje JC, et al. Anandamide levels fluctuate in the bovine oviduct during the oestrous cycle. PloS one. 2013;8(8):e72521. doi: 10.1371/journal.pone.0072521. PubMed PMID: 23977311; PubMed Central PMCID: PMC3745412.

A160. Ghirardi JJ, Caja G, Garin D, Casellas J, Hernandez-Jover M. Evaluation of the retention of electronic identification boluses in the forestomachs of cattle. Journal of animal science. 2006;84(8):2260-8. doi: 10.2527/jas.2005-758. PubMed PMID: 16864888.

A161. Gidron A, Tallman MS. 2-CdA in the treatment of hairy cell leukemia: a review of long-term follow-up. Leukemia & lymphoma. 2006;47(11):2301-7. doi: 10.1080/10428190600822052. PubMed PMID: 17107901.

A162. Gilchrest BA. If it's not the hamburgers, it's the sunscreens. The Journal of investigative dermatology. 2004;123(1):xi-xii. doi: 10.1111/j.0022-202X.2004.22740.x. PubMed PMID: 15191577.

A163. Glaser D. Re: Khalili et al.--chronic Hepatitis C treatment in interferon nonresponders. The American journal of gastroenterology. 2000;95(11):3315-6. doi: 10.1111/j.1572-0241.2000.03315.x. PubMed PMID: 11095370.

A164. Glud SZ, Sorensen AB, Andrulis M, Wang B, Kondo E, Jessen R, et al. A tumor-suppressor function for NFATc3 in T-cell lymphomagenesis by murine leukemia virus. Blood. 2005;106(10):3546-52. doi: 10.1182/blood-2005-02-0493. PubMed PMID: 16051745; PubMed Central PMCID: PMC1895049.

A165. Golde TE, Eckman CB, Younkin SG. Biochemical detection of Abeta isoforms: implications for pathogenesis, diagnosis, and treatment of Alzheimer's disease. Biochimica et biophysica acta. 2000;1502(1):172-87. PubMed PMID: 10899442.

A166. Grabowski GA. Gaucher's disease. Enzyme therapy is not enough. Lancet. 2001;358 Suppl:S29. PubMed PMID: 11784578.

A167. Green E, Ingoglia JN, Phillips C. Share what works: model practices in local public health agencies. Journal of public health management and practice : JPHMP. 2004;10(2):180-2. PubMed PMID: 14967987.

A168. Gregori Junior F. Conservative surgical management of mitral insufficiency: an alternative approach. Revista brasileira de cirurgia cardiovascular : orgao oficial da Sociedade Brasileira de Cirurgia Cardiovascular. 2012;27(2):312-7. PubMed PMID: 22996984.

A169. Gregory RJ, DeLucia-Deranja E, Mogle JA. Dynamic deconstructive psychotherapy versus optimized community care for borderline personality disorder co-occurring with alcohol use disorders: a 30-month follow-up. The Journal of nervous and mental disease. 2010;198(4):292-8. doi: 10.1097/NMD.0b013e3181d6172d. PubMed PMID: 20386259.

A170. Gregory RL. Editorial essay: magic. Perception. 2006;35(10):1295-6. PubMed PMID: 17214376.

A171. Greven H, Richter S. Morphology of skin incubation in Pipa carvalhoi (Anura: Pipidae). Journal of morphology. 2009;270(11):1311-9. doi: 10.1002/jmor.10759. PubMed PMID: 19434720.

A172. Grohn A, Suonmaa V, Auvinen A, Lehtinen KE, Jokiniemi J. Reduction of fine particle emissions from wood combustion with optimized condensing heat exchangers. Environmental science & technology. 2009;43(16):6269-74. PubMed PMID: 19746724.

A173. Gros F. From the messenger RNA saga to the transcriptome era. Comptes rendus biologies. 2003;326(10-11):893-900. PubMed PMID: 14744095.

A174. Grucza RA, Plunk AD, Hipp PR, Cavazos-Rehg P, Krauss MJ, Brownson RC, et al. Long-term effects of laws governing youth access to tobacco. American journal of public health. 2013;103(8):1493-9. doi: 10.2105/AJPH.2012.301123. PubMed PMID: 23763414; PubMed Central PMCID: PMC3710295.

A175. Guan B, Chen X, Zhang H. Two-electrode voltage clamp. Methods in molecular biology. 2013;998:79-89. doi: 10.1007/978-1-62703-351-0_6. PubMed PMID: 23529422.

A176. Guo P, Wang J, Pan DC, Xu GH. Poly[diaquabis(mu(3)-hexamethylenetetramine)[mu(2)-2,2'-(piperazine-1,4-diyl)bis(ethanesulfonato)]disilver(I)]: a three-dimensional pillared-layer framework encapsulating a water chain of (H(2)O)(12) clusters. Acta crystallographica Section C, Crystal structure communications. 2010;66(Pt 9):m266-8. doi: 10.1107/S0108270110032609. PubMed PMID: 20814100.

A177. Haas LF. Neurological stamp: Paul Ehrlich (1854-1915) and Emil Adolf von Behring (1854-1917). Journal of neurology, neurosurgery, and

psychiatry. 2001;70(5):678. PubMed PMID: 11309466; PubMed Central PMCID: PMC1737363.

A178. Hale FA. Crown lengthening for mandibular and maxillary canine teeth in the dog. Journal of veterinary dentistry. 2001;18(4):219-21. PubMed PMID: 11968902.

A179. Hallock GG. Sequential bilateral breast reconstruction using a split solitary deep inferior epigastric artery perforator flap. Journal of reconstructive microsurgery. 2012;28(9):573-5. doi: 10.1055/s-0032-1315781. PubMed PMID: 22744898.

A180. Hao Y, Hao G, Qiu S, Wang B. Effects of age and gender on the likelihood of hip fracture in the elderly population in Shanghai, China. Saudi medical journal. 2009;30(11):1483-5. PubMed PMID: 19882067.

A181. Hassan SF, Rashid U, Ansari FL, Ul-Haq Z. Bioisosteric approach in designing new monastrol derivatives: an investigation on their ADMET prediction using in silico derived parameters. Journal of molecular graphics & modelling. 2013;45:202-10. doi: 10.1016/j.jmgm.2013.09.002. PubMed PMID: 24080467.

A182. Hausberg M, Hillebrand U, Kisters K. Addressing the relationship between sympathetic activity and inflammation. Journal of hypertension. 2008;26(12):2276-8. doi: 10.1097/HJH.0b013e328318efd4. PubMed PMID: 19008705.

A183. Hayashi S, Hatashita M, Hayashi A, Matsumoto H, Shioura H, Kitai R. Thermosensitization by parthenolide in human lung adenocarcinoma A549

cells and p53- and hsp72-independent apoptosis induction via the nuclear factor-kappaB signal pathway. International journal of molecular medicine. 2008;21(5):585-92. PubMed PMID: 18425350.

A184. Hayreh SS. Posterior ischaemic optic neuropathy: clinical features, pathogenesis, and management. Eye. 2004;18(11):1188-206. doi: 10.1038/sj.eye.6701562. PubMed PMID: 15534605.

A185. Hede K. Stem cell treatments raise thorny questions for researchers, clinicians. Journal of the National Cancer Institute. 2012;104(5):347-9. doi: 10.1093/jnci/djs154. PubMed PMID: 22349201.

A186. Helinck S, Le Bars D, Moreau D, Yvon M. Ability of thermophilic lactic acid bacteria to produce aroma compounds from amino acids. Applied and environmental microbiology. 2004;70(7):3855-61. doi: 10.1128/AEM.70.7.3855-3861.2004. PubMed PMID: 15240255; PubMed Central PMCID: PMC444772.

A187. Herde M, Koo AJ, Howe GA. Elicitation of jasmonate-mediated defense responses by mechanical wounding and insect herbivory. Methods in molecular biology. 2013;1011:51-61. doi: 10.1007/978-1-62703-414-2_5. PubMed PMID: 23615987.

A188. Hickey B, Heneghan C, de Chazal P. Non-episode-dependent assessment of paroxysmal atrial fibrillation through measurement of RR interval dynamics and atrial premature contractions. Annals of biomedical engineering. 2004;32(5):677-87. PubMed PMID: 15171622.

A189. Higuchi T, Shiraishi T, Shirakusa T, Hirayama S, Shibaguchi H, Kuroki M, et al. Prevention of acute lung allograft rejection in rat by the janus kinase 3 inhibitor, tyrphostin AG490. The Journal of heart and lung transplantation : the official publication of the International Society for Heart Transplantation. 2005;24(10):1557-64. doi: 10.1016/j.healun.2004.11.017. PubMed PMID: 16210130.

A190. Hill PC, Jackson-Sillah D, Fox A, Franken KL, Lugos MD, Jeffries DJ, et al. ESAT-6/CFP-10 fusion protein and peptides for optimal diagnosis of mycobacterium tuberculosis infection by ex vivo enzyme-linked immunospot assay in the Gambia. Journal of clinical microbiology. 2005;43(5):2070-4. doi: 10.1128/JCM.43.5.2070-2074.2005. PubMed PMID: 15872224; PubMed Central PMCID: PMC1153808.

A191. Hill-Briggs F, Renosky R, Lazo M, Bone L, Hill M, Levine D, et al. Development and pilot evaluation of literacy-adapted diabetes and CVD education in urban, diabetic African Americans. Journal of general internal medicine. 2008;23(9):1491-4. doi: 10.1007/s11606-008-0679-9. PubMed PMID: 18521688; PubMed Central PMCID: PMC2518002.

A192. Hoffman JN, Faist E. Coagulation inhibitor replacement during sepsis: useless? Critical care medicine. 2000;28(9 Suppl):S74-6. PubMed PMID: 11007203.

A193. Hoi Y, Wasserman BA, Xie YJ, Najjar SS, Ferruci L, Lakatta EG, et al. Characterization of volumetric flow rate waveforms at the carotid bifurcations of older adults. Physiological measurement. 2010;31(3):291-

302. doi: 10.1088/0967-3334/31/3/002. PubMed PMID: 20086276; PubMed Central PMCID: PMC2943236.

A194. Hollon SD, Jarrett RB, Nierenberg AA, Thase ME, Trivedi M, Rush AJ. Psychotherapy and medication in the treatment of adult and geriatric depression: which monotherapy or combined treatment? The Journal of clinical psychiatry. 2005;66(4):455-68. PubMed PMID: 15816788.

A195. Honeycutt RL, Rowe DL, Gallardo MH. Molecular systematics of the South American caviomorph rodents: relationships among species and genera in the family Octodontidae. Molecular phylogenetics and evolution. 2003;26(3):476-89. PubMed PMID: 12644405.

A196. Hoopes MI, Faller R, Longo ML. Lipid domain depletion at small localized bends imposed by a step geometry. Langmuir : the ACS journal of surfaces and colloids. 2011;27(6):2783-8. doi: 10.1021/la104504p. PubMed PMID: 21338070.

A197. Hori K, Yamakawa M, Tanaka N, Murakami H, Kaya M, Hori S. Influence of sound and light on heart rate variability. Journal of human ergology. 2005;34(1-2):25-34. PubMed PMID: 17393762.

A198. Horiguchi K, Fujiwara K, Ilmiawati C, Kikuchi M, Tsukada T, Kouki T, et al. Caveolin 3-mediated integrin beta1 signaling is required for the proliferation of folliculostellate cells in rat anterior pituitary gland under the influence of extracellular matrix. The Journal of endocrinology. 2011;210(1):29-36. doi: 10.1530/JOE-11-0103. PubMed PMID: 21508095.

A199. Horowitz JC, Ajayi IO, Kulasekaran P, Rogers DS, White JB, Townsend SK, et al. Survivin expression induced by endothelin-1 promotes myofibroblast resistance to apoptosis. The international journal of biochemistry & cell biology. 2012;44(1):158-69. doi: 10.1016/j.biocel.2011.10.011. PubMed PMID: 22041029; PubMed Central PMCID: PMC3241828.

A200. Huang KL, Tsai SJ. St. John's wort (Hypericum perforatum) as a treatment for premenstrual dysphoric disorder: case report. International journal of psychiatry in medicine. 2003;33(3):295-7. PubMed PMID: 15089009.

A201. Huey ED. Frontotemporal dementia. The American journal of psychiatry. 2014;171(6):695-6. doi: 10.1176/appi.ajp.2014.14020215. PubMed PMID: 24880519.

A202. Humpert PM, Neuwirth R, Battista MJ, Voronko O, von Eynatten M, Konrade I, et al. SDF-1 genotype influences insulin-dependent mobilization of adult progenitor cells in type 2 diabetes. Diabetes care. 2005;28(4):934-6. PubMed PMID: 15793201.

A203. Hur YM, Kwon JS. Changes in twinning rates in South Korea: 1981-2002. Twin research and human genetics : the official journal of the International Society for Twin Studies. 2005;8(1):76-9. doi: 10.1375/1832427053435445. PubMed PMID: 15836815.

A204. Hutcheson JD, Setola V, Roth BL, Merryman WD. Serotonin receptors and heart valve disease--it was meant 2B. Pharmacology & therapeutics.

2011;132(2):146-57. doi: 10.1016/j.pharmthera.2011.03.008. PubMed PMID: 21440001; PubMed Central PMCID: PMC3179857.

A205. Huttenhower C, Hibbs M, Myers C, Troyanskaya OG. A scalable method for integration and functional analysis of multiple microarray datasets. Bioinformatics. 2006;22(23):2890-7. doi: 10.1093/bioinformatics/btl492. PubMed PMID: 17005538.

A206. Hutter HP, Moshammer H, Wallner P, Cartellieri M, Denk-Linnert DM, Katzinger M, et al. Tinnitus and mobile phone use. Occupational and environmental medicine. 2010;67(12):804-8. doi: 10.1136/oem.2009.048116. PubMed PMID: 20573849.

A207. Iijima M, Brantley WA, Guo WH, Clark WA, Yuasa T, Mizoguchi I. X-ray diffraction study of low-temperature phase transformations in nickel-titanium orthodontic wires. Dental materials : official publication of the Academy of Dental Materials. 2008;24(11):1454-60. doi: 10.1016/j.dental.2008.03.005. PubMed PMID: 18452984.

A208. Iino Y, Tanaka M, Hatano N, Kiyokawa M, Takebayashi H, Shinohara I, et al. A case of a five-year-old boy with acute retinal necrosis. Retina. 2002;22(2):225-8. PubMed PMID: 11927861.

A209. Ikeda Y, Imai Y, Kumagai H, Nosaka T, Morikawa Y, Hisaoka T, et al. Vasorin, a transforming growth factor beta-binding protein expressed in vascular smooth muscle cells, modulates the arterial response to injury in vivo. Proceedings of the National Academy of Sciences of the United

States of America. 2004;101(29):10732-7. doi: 10.1073/pnas.0404117101. PubMed PMID: 15247411; PubMed Central PMCID: PMC490003.

A210. Inoue Y, Ogasawara M, Moroi T, Satake M, Azumi K, Moritomo T, et al. Characteristics of NADPH oxidase genes (Nox2, p22, p47, and p67) and Nox4 gene expressed in blood cells of juvenile Ciona intestinalis. Immunogenetics. 2005;57(7):520-34. doi: 10.1007/s00251-005-0010-4. PubMed PMID: 16025326.

A211. Ito R, Umezawa N, Higuchi T. Unique oxidation reaction of amides with pyridine-N-oxide catalyzed by ruthenium porphyrin: direct oxidative conversion of N-acyl-L-proline to N-acyl-L-glutamate. Journal of the American Chemical Society. 2005;127(3):834-5. doi: 10.1021/ja045603f. PubMed PMID: 15656611.

A212. Ivekovic D, Milardovic S, Roboz M, Grabaric BS. Evaluation of the antioxidant activity by flow injection analysis method with electrochemically generated ABTS radical cation. The Analyst. 2005;130(5):708-14. doi: 10.1039/b415939j. PubMed PMID: 15852141.

A213. Iyer U, Mathur G, Panchanmiya N, Dhruv S. Risk Factor Scenario in an Industrial Set-up: Need for an Effective Screening Tool to Assess the High-Risk Group. Indian journal of community medicine : official publication of Indian Association of Preventive & Social Medicine. 2010;35(2):262-6. doi: 10.4103/0970-0218.66884. PubMed PMID: 20922103; PubMed Central PMCID: PMC2940182.

A214. Jager M, Knoll C, Hamprecht FA. Weakly supervised learning of a classifier for unusual event detection. IEEE transactions on image processing : a publication of the IEEE Signal Processing Society. 2008;17(9):1700-8. doi: 10.1109/TIP.2008.2001043. PubMed PMID: 18701402.

A215. Jain NB, Higgins LD. Re: "Treatment options in knee osteoarthritis: total knee arthroplasty versus platelet-rich plasma". PM & R : the journal of injury, function, and rehabilitation. 2011;3(7):681-2; author reply 2. doi: 10.1016/j.pmrj.2011.04.017. PubMed PMID: 21777870.

A216. Jerng HH, Lauver AD, Pfaffinger PJ. DPP10 splice variants are localized in distinct neuronal populations and act to differentially regulate the inactivation properties of Kv4-based ion channels. Molecular and cellular neurosciences. 2007;35(4):604-24. doi: 10.1016/j.mcn.2007.03.008. PubMed PMID: 17475505; PubMed Central PMCID: PMC3674967.

A217. Johnson TW, Wassersug RJ. Gender identity disorder outside the binary: when gender identity disorder-not otherwise specified is not good enough. Archives of sexual behavior. 2010;39(3):597-8. doi: 10.1007/s10508-010-9608-1. PubMed PMID: 20182785.

A218. Joyce MG, Sun PD. The structural basis of ligand recognition by natural killer cell receptors. Journal of biomedicine & biotechnology. 2011;2011:203628. doi: 10.1155/2011/203628. PubMed PMID: 21629745; PubMed Central PMCID: PMC3100565.

A219. Jun HW, Taite LJ, West JL. Nitric oxide-producing polyurethanes. Biomacromolecules. 2005;6(2):838-44. doi: 10.1021/bm049419y. PubMed PMID: 15762649.

A220. Junkin M, Lu Y, Long J, Deymier PA, Hoying JB, Wong PK. Mechanically induced intercellular calcium communication in confined endothelial structures. Biomaterials. 2013;34(8):2049-56. doi: 10.1016/j.biomaterials.2012.11.060. PubMed PMID: 23267827; PubMed Central PMCID: PMC3542404.

A221. Jyvakorpi SK, Puranen T, Pitkala KH, Suominen MH. Nutritional treatment of aged individuals with Alzheimer disease living at home with their spouses: study protocol for a randomized controlled trial. Trials. 2012;13:66. doi: 10.1186/1745-6215-13-66. PubMed PMID: 22624652; PubMed Central PMCID: PMC3517368.

A222. Kaassis M, Boyer J, Dumas R, Ponchon T, Coumaros D, Delcenserie R, et al. Plastic or metal stents for malignant stricture of the common bile duct? Results of a randomized prospective study. Gastrointestinal endoscopy. 2003;57(2):178-82. doi: 10.1067/mge.2003.66. PubMed PMID: 12556780.

A223. Kahaleh M, Hermans P, Buset M, Dargent JL. Primary neutrophil-rich, CD30-positive anaplastic large cell lymphoma of the stomach: case report and review of the literature. Acta gastro-enterologica Belgica. 2002;65(4):237-40. PubMed PMID: 12619433.

A224. Kajiho H, Saito K, Tsujita K, Kontani K, Araki Y, Kurosu H, et al. RIN3: a novel Rab5 GEF interacting with amphiphysin II involved in the early endocytic pathway. Journal of cell science. 2003;116(Pt 20):4159-68. doi: 10.1242/jcs.00718. PubMed PMID: 12972505.

A225. Kalman D, Janosi S, Egyed L. Role of bovine herpesvirus 4 in bacterial bovine mastitis. Microbial pathogenesis. 2004;37(3):125-9. doi: 10.1016/j.micpath.2004.06.011. PubMed PMID: 15351035.

A226. Kang CM, Choi SH, Kim SC, Lee WJ, Choi DW, Kim SW, et al. Predicting Recurrence of Pancreatic Solid Pseudopapillary Tumors After Surgical Resection: A Multicenter Analysis in Korea. Annals of surgery. 2014. doi: 10.1097/SLA.0000000000000583. PubMed PMID: 24743622.

A227. Kang HS, Roh HG, Han MH, Koh YC. Successful endovascular occlusion of a ruptured distal anterior inferior cerebellar artery aneurysm of the caudal trunk: case report. Interventional neuroradiology : journal of peritherapeutic neuroradiology, surgical procedures and related neurosciences. 2007;13(3):271-6. PubMed PMID: 20566118; PubMed Central PMCID: PMC3345489.

A228. Kato K, Hara A, Kuno T, Mori H, Yamashita T, Toida M, et al. Aberrant promoter hypermethylation of p16 and MGMT genes in oral squamous cell carcinomas and the surrounding normal mucosa. Journal of cancer research and clinical oncology. 2006;132(11):735-43. doi: 10.1007/s00432-006-0122-8. PubMed PMID: 16791592.

A229. Kato Y, Ogiwara K, Fujimori C, Kimura A, Takahashi T. Expression and localization of collagen type IV alpha1 chain in medaka ovary. Cell and tissue research. 2010;340(3):595-605. doi: 10.1007/s00441-010-0969-5. PubMed PMID: 20424863.

A230. Kestin SC. Review of welfare concerns relating to commercial and special permit (scientific) whaling. The Veterinary record. 2001;148(10):304-7. PubMed PMID: 11315136.

A231. Kido VJ. The UAP dilemma. Nursing management. 2001;32(11):27-9. PubMed PMID: 15129540.

A232. Kiecolt-Glaser JK, McGuire L, Robles TF, Glaser R. Psychoneuroimmunology: psychological influences on immune function and health. Journal of consulting and clinical psychology. 2002;70(3):537-47. PubMed PMID: 12090368.

A233. Kieran NE, Rabb H. Immune responses in kidney preservation and reperfusion injury. Journal of investigative medicine : the official publication of the American Federation for Clinical Research. 2004;52(5):310-4. PubMed PMID: 15551653.

A234. Kikuchi H, Sakurai H, Nagura T, Aritsuka T, Tomita F, Yokota A. One-pot conversion of levan prepared from Serratia levanicum NN to difructose anhydride IV by Arthrobacter nicotinovorans levan fructotransferase. Journal of bioscience and bioengineering. 2010;109(3):240-3. doi: 10.1016/j.jbiosc.2009.09.041. PubMed PMID: 20159571.

A235. Killiany RJ. Isn't amyloid more than just a marker for Alzheimer disease? Neurology. 2009;73(15):1174-5. doi: 10.1212/WNL.0b013e3181be020a. PubMed PMID: 19741212.

A236. Kim DG, Park SC, Moon IS, Lee MD. Reconstruction of three right hepatic veins using cadaveric iliac vein in right lobe living donor liver transplantation: case report. Transplantation proceedings. 2008;40(8):2523-4. doi: 10.1016/j.transproceed.2008.07.022. PubMed PMID: 18929787.

A237. Kipshidze N, Rukhadze I, Archvadze A, Kipiani V, Kipshidze N, Lapiashvili E, et al. Endovascular treatment of patients with chronic cerebrospinal venous insufficiency and multiple sclerosis. Georgian medical news. 2011;(199):29-34. PubMed PMID: 22155803.

A238. Kiray A, Ergur I, Tayefi H, Bagriyanik HA, Bacakoglu AK. Anatomical evaluation of the superficial veins of the upper extremity as graft donor source in microvascular reconstructions: a cadaveric study. Acta orthopaedica et traumatologica turcica. 2013;47(6):405-10. PubMed PMID: 24509220.

A239. Kirikyali N, Connerton IF. Heterologous expression and kinetic characterisation of Neurospora crassa beta-xylosidase in Pichia pastoris. Enzyme and microbial technology. 2014;57:63-8. doi: 10.1016/j.enzmictec.2014.02.002. PubMed PMID: 24629269.

A240. Klein IF, Lavallee PC, Schouman-Claeys E, Amarenco P. High-resolution MRI identifies basilar artery plaques in paramedian pontine infarct.

Neurology. 2005;64(3):551-2. doi: 10.1212/01.WNL.0000150543.61244.06. PubMed PMID: 15699395.

A241. Koide M, Nishizawa S, Ohta S, Yokoyama T, Namba H. Chronological changes of the contractile mechanism in prolonged vasospasm after subarachnoid hemorrhage: from protein kinase C to protein tyrosine kinase. Neurosurgery. 2002;51(6):1468-74; discussion 74-6. PubMed PMID: 12445353.

A242. Koliofoti EG, Gkentzi D, Varvarigou A, Trigka M, Schulpis K. Rare case of homozygous epimerase deficiency and heterozygous of duarte 2 variant. Journal of pediatric endocrinology & metabolism : JPEM. 2014. doi: 10.1515/jpem-2013-0431. PubMed PMID: 24859500.

A243. Kolvenbach R. The role of periscopes and chimneys in complex aneurysm cases. Journal of endovascular therapy : an official journal of the International Society of Endovascular Specialists. 2011;18(5):661-5. doi: 10.1583/11-3504C.1. PubMed PMID: 21992636.

A244. Kondo T, Oshima T, Tomita T, Fukui H, Watari J, Okada H, et al. Prostaglandin E(2) mediates acid-induced heartburn in healthy volunteers. American journal of physiology Gastrointestinal and liver physiology. 2013;304(6):G568-73. doi: 10.1152/ajpgi.00276.2012. PubMed PMID: 23370674.

A245. Kong Y, Wang Y, Zhang JH, Wang X, Qin X. Disparities in medical expenditure and outcomes among patients with intracranial hemorrhage associated with different insurance statuses in southwestern China. Acta

neurochirurgica Supplement. 2011;111:337-41. doi: 10.1007/978-3-7091-0693-8_56. PubMed PMID: 21725778.

A246. Koo TK, Cohen JH, Zheng Y. Immediate effect of nimmo receptor tonus technique on muscle elasticity, pain perception, and disability in subjects with chronic low back pain. Journal of manipulative and physiological therapeutics. 2012;35(1):45-53. doi: 10.1016/j.jmpt.2011.09.013. PubMed PMID: 22036530.

A247. Kramer MA, Tracy TS. Studying cytochrome P450 kinetics in drug metabolism. Expert opinion on drug metabolism & toxicology. 2008;4(5):591-603. doi: 10.1517/17425255.4.5.591. PubMed PMID: 18484917.

A248. Kravchuk ES, Gladyshev MI, Ivanova EA. Dependence of the domination of Anabaenaflos-aquae (Lyngb.) Breb. in the phytoplankton community on the initial abundance of akinetes. Doklady biological sciences : proceedings of the Academy of Sciences of the USSR, Biological sciences sections / translated from Russian. 2007;416:377-8. PubMed PMID: 18047023.

A249. La Torre D. Immunobiology of beta-cell destruction. Advances in experimental medicine and biology. 2012;771:194-218. PubMed PMID: 23393680.

A250. Labuguen RH. Initial evaluation of vertigo. American family physician. 2006;73(2):244-51. PubMed PMID: 16445269.

A251. Lam DK, Schmidt BL. Orofacial pain onset predicts transition to head and neck cancer. Pain. 2011;152(5):1206-9. doi: 10.1016/j.pain.2011.02.009. PubMed PMID: 21388740; PubMed Central PMCID: PMC3099418.

A252. Lamas LP, Giovagnoli G, Heath MF, Jeffcott LB. Some factors affecting the accuracy and variability of measurements of the height of ponies. The Veterinary record. 2007;160(20):691-4. PubMed PMID: 17513835.

A253. Lan C, Chen SY, Lai JS. Changes of aerobic capacity, fat ratio and flexibility in older TCC practitioners: a five-year follow-up. The American journal of Chinese medicine. 2008;36(6):1041-50. doi: 10.1142/S0192415X08006442. PubMed PMID: 19051334.

A254. Largo-Wight E, Peterson PM, Chen WW. Perceived problem solving, stress, and health among college students. American journal of health behavior. 2005;29(4):360-70. PubMed PMID: 16006233.

A255. Larkin M. On the rise: internships under scrutiny as supply, demand continue to increase despite pay, hours. Journal of the American Veterinary Medical Association. 2013;243(1):8-11. doi: 10.2460/javma.243.1.8. PubMed PMID: 23786186.

A256. Lau Y, Chan KS. Influence of intimate partner violence during pregnancy and early postpartum depressive symptoms on breastfeeding among chinese women in Hong Kong. Journal of midwifery & women's health. 2007;52(2):e15-20. doi: 10.1016/j.jmwh.2006.09.001. PubMed PMID: 17336812.

A257. Lauriano CM, Ghosh C, Correa NE, Klose KE. The sodium-driven flagellar motor controls exopolysaccharide expression in Vibrio cholerae. Journal of bacteriology. 2004;186(15):4864-74. doi: 10.1128/JB.186.15.4864-4874.2004. PubMed PMID: 15262923; PubMed Central PMCID: PMC451641.

A258. Le DT, Tran TL, Duviau MP, Meyrand M, Guerardel Y, Castelain M, et al. Unraveling the role of surface mucus-binding protein and pili in muco-adhesion of Lactococcus lactis. PloS one. 2013;8(11):e79850. doi: 10.1371/journal.pone.0079850. PubMed PMID: 24260308; PubMed Central PMCID: PMC3832589.

A259. Lee A, Okayasu K, Wang HL. Screw- versus cement-retained implant restorations: current concepts. Implant dentistry. 2010;19(1):8-15. doi: 10.1097/ID.0b013e3181bb9033. PubMed PMID: 20147811.

A260. Lee HJ, Goo JM, Im JG. Rapid and irreversible cystic change of pulmonary tuberculosis in an immunocompetent adult. Journal of thoracic imaging. 2003;18(4):254-6. PubMed PMID: 14561913.

A261. Lee J, Lim do H, Kim S, Park SH, Park JO, Park YS, et al. Phase III trial comparing capecitabine plus cisplatin versus capecitabine plus cisplatin with concurrent capecitabine radiotherapy in completely resected gastric cancer with D2 lymph node dissection: the ARTIST trial. Journal of clinical oncology : official journal of the American Society of Clinical Oncology. 2012;30(3):268-73. doi: 10.1200/JCO.2011.39.1953. PubMed PMID: 22184384.

A262. Lee SY, Cho JS, Yuk DY, Moon DC, Jung JK, Yoo HS, et al. Obovatol enhances docetaxel-induced prostate and colon cancer cell death through inactivation of nuclear transcription factor-kappaB. Journal of pharmacological sciences. 2009;111(2):124-36. PubMed PMID: 19834284.

A263. Lee YF, Lin CC, Chen GS. Temporal course of streptozotocin-induced diabetic polyneuropathy in rats. Neurological sciences : official journal of the Italian Neurological Society and of the Italian Society of Clinical Neurophysiology. 2014. doi: 10.1007/s10072-014-1848-8. PubMed PMID: 24924783.

A264. Leone M, Albanese J, Tod M, Savelli V, Ragni E, Rossi D, et al. Ceftriaxone (1 g intravenously) penetration into abdominal tissues when administered as antibiotic prophylaxis during nephrectomy. Journal of chemotherapy. 2003;15(2):139-42. doi: 10.1179/joc.2003.15.2.139. PubMed PMID: 12797390.

A265. Letsas KP, Filippatos GS, Kounas SP, Efremidis M, Sideris A, Kardaras F. QT interval prolongation and Torsades de Pointes in a patient receiving zolpidem and amiodarone. Cardiology. 2006;105(3):146-7. doi: 10.1159/000091201. PubMed PMID: 16449812.

A266. Lewis G. Viscoelastic properties of injectable bone cements for orthopaedic applications: state-of-the-art review. Journal of biomedical materials research Part B, Applied biomaterials. 2011;98(1):171-91. doi: 10.1002/jbm.b.31835. PubMed PMID: 21504058.

A267. Liao M, Kielian M. Domain III from class II fusion proteins functions as a dominant-negative inhibitor of virus membrane fusion. The Journal of cell biology. 2005;171(1):111-20. doi: 10.1083/jcb.200507075. PubMed PMID: 16216925; PubMed Central PMCID: PMC2171229.

A268. Licker M, Diaper J, Villiger Y, Spiliopoulos A, Licker V, Robert J, et al. Impact of intraoperative lung-protective interventions in patients undergoing lung cancer surgery. Critical care. 2009;13(2):R41. doi: 10.1186/cc7762. PubMed PMID: 19317902; PubMed Central PMCID: PMC2689485.

A269. Lin WC, Liu CP, Kuo CH, Chang HY, Chang CJ, Hsieh TH, et al. The role of the auxiliary atomic ion beam in C60(+)-Ar+ co-sputtering. The Analyst. 2011;136(5):941-6. doi: 10.1039/c0an00642d. PubMed PMID: 21152650.

A270. Little DM, Hartley AA. Further evidence that negative priming in the Stroop color-word task is equivalent in older and younger adults. Psychology and aging. 2000;15(1):9-17. PubMed PMID: 10755285.

A271. Liu L, Xu X, Zhuang D, Chen X, Li S. Changes in the potential multiple cropping system in response to climate change in China from 1960-2010. PloS one. 2013;8(12):e80990. doi: 10.1371/journal.pone.0080990. PubMed PMID: 24312511; PubMed Central PMCID: PMC3849037.

A272. Liu S, Zhao C, Yang C, Li X, Huang H, Liu N, et al. Gambogic acid suppresses pressure overload cardiac hypertrophy in rats. American journal of cardiovascular disease. 2013;3(4):227-38. PubMed PMID: 24224134; PubMed Central PMCID: PMC3819582.

A273. Loewen CA, Mackler JM, Reist NE. Drosophila synaptotagmin I null mutants survive to early adulthood. Genesis. 2001;31(1):30-6. PubMed PMID: 11668675.

A274. Lombardo Y, Scopelliti A, Cammareri P, Todaro M, Iovino F, Ricci-Vitiani L, et al. Bone morphogenetic protein 4 induces differentiation of colorectal cancer stem cells and increases their response to chemotherapy in mice. Gastroenterology. 2011;140(1):297-309. doi: 10.1053/j.gastro.2010.10.005. PubMed PMID: 20951698.

A275. Lomholt JF, Nolting D, Hansen BF, Stoltze K, Kjaer I. The pre-natal development and osseous growth of the human cerebellar field. Orthodontics & craniofacial research. 2003;6(3):143-54. PubMed PMID: 12962197.

A276. Lorenzana R, Coustan-Smith E, Antillon F, Ribeiro RC, Campana D. Simple methods for the rapid exchange of flow cytometric data between remote centers. Leukemia. 2000;14(2):336-7. PubMed PMID: 10673754.

A277. Lowe AW, Moseley RH. Noninvasive assessment of longterm outcomes in patients with nonalcoholic fatty liver disease. Gastroenterology. 2013;145(4):699-700. PubMed PMID: 24187687.

A278. Lowenberg B, Morgan G, Ossenkoppele GJ, Burnett AK, Zachee P, Duhrsen U, et al. Phase I/II clinical study of Tosedostat, an inhibitor of aminopeptidases, in patients with acute myeloid leukemia and myelodysplasia. Journal of clinical oncology : official journal of the

American Society of Clinical Oncology. 2010;28(28):4333-8. doi: 10.1200/JCO.2009.27.6295. PubMed PMID: 20733120.

A279. Lu Q, Longo FM, Zhou H, Massa SM, Chen YH. Signaling through Rho GTPase pathway as viable drug target. Current medicinal chemistry. 2009;16(11):1355-65. PubMed PMID: 19355891; PubMed Central PMCID: PMC3829470.

A280. Lv SQ, Zhang KB, Zhang EE, Gao FY, Yin CL, Huang CJ, et al. Antitumor efficiency of the cytosine deaminase/5-fluorocytosine suicide gene therapy system on malignant gliomas: an in vivo study. Medical science monitor : international medical journal of experimental and clinical research. 2009;15(1):BR13-20. PubMed PMID: 19114960.

A281. Lyell DJ, Pullen K, Fuh K, Zamah AM, Caughey AB, Benitz W, et al. Daily compared with 8-hour gentamicin for the treatment of intrapartum chorioamnionitis: a randomized controlled trial. Obstetrics and gynecology. 2010;115(2 Pt 1):344-9. doi: 10.1097/AOG.0b013e3181cb5c0e. PubMed PMID: 20093909.

A282. Lyons WS. Is it time to change the rules for resuscitation? American journal of surgery. 2002;184(6):658; author reply -60. PubMed PMID: 12488208.

A283. Mackenbach JP. Streets of Paris, sunflower seeds, and Nobel prizes. Reflections on the quantitative paradigm of public health. Journal of epidemiology and community health. 2004;58(9):734-7. doi:

10.1136/jech.2003.016139. PubMed PMID: 15310796; PubMed Central PMCID: PMC1732889.

A284. Mahnke YD, Schirrmacher V. A novel tumour model system for the study of long-term protective immunity and immune T cell memory. Cellular immunology. 2003;221(2):89-99. PubMed PMID: 12747949.

A285. Maine GT, Stricker R, Schuler M, Spesard J, Brojanac S, Iriarte B, et al. Development and clinical evaluation of a recombinant-antigen-based cytomegalovirus immunoglobulin M automated immunoassay using the Abbott AxSYM analyzer. Journal of clinical microbiology. 2000;38(4):1476-81. PubMed PMID: 10747129; PubMed Central PMCID: PMC86469.

A286. Mandell BF. Why effective drugs don't work for everyone. Cleveland Clinic journal of medicine. 2007;74(1):3. PubMed PMID: 17373341.

A287. Mao HZ, Roussos ET, Peterfy M. Genetic analysis of the diabetes-prone C57BLKS/J mouse strain reveals genetic contribution from multiple strains. Biochimica et biophysica acta. 2006;1762(4):440-6. doi: 10.1016/j.bbadis.2006.01.002. PubMed PMID: 16481151.

A288. Marathe D, Mishra KP. Radiation-induced changes in permeability in unilamellar phospholipid liposomes. Radiation research. 2002;157(6):685-92. PubMed PMID: 12005548.

A289. Mark KS, Davis TP. Cerebral microvascular changes in permeability and tight junctions induced by hypoxia-reoxygenation. American journal of physiology Heart and circulatory physiology. 2002;282(4):H1485-94. doi:

10.1152/ajpheart.00645.2001. PubMed PMID: 11893586; PubMed Central PMCID: PMC3918411.

A290. Marques J, Moles E, Urban P, Prohens R, Busquets MA, Sevrin C, et al. Application of heparin as a dual agent with antimalarial and liposome targeting activities towards Plasmodium-infected red blood cells. Nanomedicine : nanotechnology, biology, and medicine. 2014. doi: 10.1016/j.nano.2014.06.002. PubMed PMID: 24941466.

A291. Martinez E, Domingo P, Galindo MJ, Milinkovic A, Arroyo JA, Baldovi F, et al. Risk of metabolic abnormalities in patients infected with HIV receiving antiretroviral therapy that contains lopinavir-ritonavir. Clinical infectious diseases : an official publication of the Infectious Diseases Society of America. 2004;38(7):1017-23. doi: 10.1086/382531. PubMed PMID: 15034836.

A292. Matsumoto T, Suzuki N, Watanabe H, Irie M, Iwata K, Anan A, et al. Nonalcoholic steatohepatitis associated with psoriasis vulgaris. Journal of gastroenterology. 2004;39(11):1102-5. doi: 10.1007/s00535-004-1451-5. PubMed PMID: 15580405.

A293. Mattoon D, Gupta K, Doyon J, Loll PJ, DiMaio D. Identification of the transmembrane dimer interface of the bovine papillomavirus E5 protein. Oncogene. 2001;20(29):3824-34. doi: 10.1038/sj.onc.1204523. PubMed PMID: 11439346.

A294. Mayer IA, Verma A, Grumbach IM, Uddin S, Lekmine F, Ravandi F, et al. The p38 MAPK pathway mediates the growth inhibitory effects of

interferon-alpha in BCR-ABL-expressing cells. The Journal of biological chemistry. 2001;276(30):28570-7. doi: 10.1074/jbc.M011685200. PubMed PMID: 11353767.

A295. McClintick J, Edenberg HJ. BlastReport: a perl script to facilitate the use of sequence databases for mapping and clustering. BioTechniques. 2000;29(6):1272-6. PubMed PMID: 11126131.

A296. Meduri B, Vergeau BM, Dumont JL, Tuszynski T, Dritsas S, Dhumane P, et al. Endoscopic ultrasound-guided fine needle aspiration and endoscopic biliary drainage following closure of a duodenal perforation with an over-the-scope clip. Endoscopy. 2014;46 Suppl 1 UCTN:E69-70. doi: 10.1055/s-0033-1359186. PubMed PMID: 24523190.

A297. Mehta PK, Baer J, Nell C, Sperling LS. Low-density lipoprotein apheresis as a treatment option for hyperlipidemia. Current treatment options in cardiovascular medicine. 2009;11(4):279-88. PubMed PMID: 19627661.

A298. Meirer R, Huemer GM, Shafighi M, Kamelger FS, Hussl H, Piza-Katzer H. Sciatic nerve enlargement in the Klippel-Trenaunay-Weber syndrome. British journal of plastic surgery. 2005;58(4):565-8. doi: 10.1016/j.bjps.2004.11.005. PubMed PMID: 15897045.

A299. Miampamba M, Germano PM, Arli S, Wong HH, Scott D, Tache Y, et al. Expression of pituitary adenylate cyclase-activating polypeptide and PACAP type 1 receptor in the rat gastric and colonic myenteric neurons. Regulatory peptides. 2002;105(3):145-54. PubMed PMID: 11959368.

A300. Mihm LM, Quick VA, Brumfield JA, Connors AF, Jr., Finnerty JJ. The accuracy of endometrial biopsy and saline sonohysterography in the determination of the cause of abnormal uterine bleeding. American journal of obstetrics and gynecology. 2002;186(5):858-60. PubMed PMID: 12015495.

A301. Mills E, Ernst E, Singh R, Ross C, Wilson K. Health food store recommendations: implications for breast cancer patients. Breast cancer research : BCR. 2003;5(6):R170-4. doi: 10.1186/bcr636. PubMed PMID: 14580252; PubMed Central PMCID: PMC314402.

A302. Milward DV. Lonely nights in long-term care. Age and ageing. 2001;30(3):271-2. PubMed PMID: 11443035.

A303. Moghadam MN, Banshi M, Javar MA, Amiresmaili M, Ganjavi S. Iranian Household Financial Protection against Catastrophic Health Care Expenditures. Iranian journal of public health. 2012;41(9):62-70. PubMed PMID: 23193508; PubMed Central PMCID: PMC3494217.

A304. Mohammad Alizadeh AH, Afzali ES, Mousavi M, Moaddab Y, Zali MR. Endoscopic retrograde cholangiopancreatography outcome from a single referral center in Iran. Hepatobiliary & pancreatic diseases international : HBPD INT. 2010;9(4):428-32. PubMed PMID: 20688609.

A305. Mok KY, Koutsis G, Schottlaender LV, Polke J, Panas M, Houlden H. High frequency of the expanded C9ORF72 hexanucleotide repeat in familial and sporadic Greek ALS patients. Neurobiology of aging. 2012;33(8):1851

e1-5. doi: 10.1016/j.neurobiolaging.2012.02.021. PubMed PMID: 22445326; PubMed Central PMCID: PMC3657168.

A306. Molardi A, Nicolini F, Beghi C, Agostinelli A, Gherli T. Aortic valve replacement with right thoracotomy in a patient with sternal metastasis from renal carcinoma. Acta bio-medica : Atenei Parmensis. 2009;80(2):150-2. PubMed PMID: 19848053.

A307. Moonka D, Castillo E, Kumer S, Abouljoud M, Divine G, Pelletier S. Impact of model for end-stage liver disease on patient survival and disease-free survival in patients receiving liver transplantation for hepatocellular carcinoma. Transplantation proceedings. 2009;41(1):216-8. doi: 10.1016/j.transproceed.2008.09.060. PubMed PMID: 19249517.

A308. Morvay Z, Szabo E, Tiszlavicz L, Furak J, Trojan I, Palko A. Thoracic core needle biopsy using ultrasound guidance. Ultrasound quarterly. 2001;17(2):113-21. PubMed PMID: 12973082.

A309. Moskvin V, Cheng CW, Fanelli L, Zhao L, Das IJ. A semi-empirical model for the therapeutic range shift estimation caused by inhomogeneities in proton beam therapy. Journal of applied clinical medical physics / American College of Medical Physics. 2012;13(2):3631. doi: 10.1120/jacmp.v13i2.3631. PubMed PMID: 22402381.

A310. Motoyama T, Yabunaka H, Miyoshi H. Essential structural factors of acetogenins, potent inhibitors of mitochondrial complex I. Bioorganic & medicinal chemistry letters. 2002;12(16):2089-92. PubMed PMID: 12127510.

A311. Mulroy J. Chronic obstructive pulmonary disease in women. Dimensions of critical care nursing : DCCN. 2005;24(1):1-18; quiz 9-20. PubMed PMID: 15714066.

A312. Nagasaka H, Yorifuji T, Egawa H, Kikuta H, Tanaka K, Kobayashi K. Successful living-donor liver transplantation from an asymptomatic carrier mother in ornithine transcarbamylase deficiency. The Journal of pediatrics. 2001;138(3):432-4. doi: 10.1067/mpd.2001.111318. PubMed PMID: 11241058.

A313. Naito M, Itoh M. Patterns of infiltration of lymphocytes into the testis under normal and pathological conditions in mice. American journal of reproductive immunology. 2008;59(1):55-61. doi: 10.1111/j.1600-0897.2007.00556.x. PubMed PMID: 18154596.

A314. Namgaladze D, Kollas A, Brune B. Oxidized LDL attenuates apoptosis in monocytic cells by activating ERK signaling. Journal of lipid research. 2008;49(1):58-65. doi: 10.1194/jlr.M700100-JLR200. PubMed PMID: 17890680.

A315. Nardone A, Grasso M, Tarantola J, Corna S, Schieppati M. Postural coordination in elderly subjects standing on a periodically moving platform. Archives of physical medicine and rehabilitation. 2000;81(9):1217-23. doi: 10.1053/apmr.2000.6286. PubMed PMID: 10987165.

A316. Nasreen N, Mohammed KA, Lai Y, Antony VB. Receptor EphA2 activation with ephrinA1 suppresses growth of malignant mesothelioma (MM).

Cancer letters. 2007;258(2):215-22. doi: 10.1016/j.canlet.2007.09.005. PubMed PMID: 17949899.

A317. Nazal L, Cardenas A. Prognostic markers in patients with ascites and hepatorenal syndrome. Disease markers. 2011;31(3):139-46. doi: 10.3233/DMA-2011-0836. PubMed PMID: 22045399; PubMed Central PMCID: PMC3826924.

A318. Niederberger C. Re: Effect of an isotonic lubricant on sperm collection and sperm quality. The Journal of urology. 2013;190(5):1843. doi: 10.1016/j.juro.2013.07.076. PubMed PMID: 24120809.

A319. Nielsen ME, Rasmussen IA, Kristensen SG, Christensen ST, Mollgard K, Wreford Andersen E, et al. In human granulosa cells from small antral follicles, androgen receptor mRNA and androgen levels in follicular fluid correlate with FSH receptor mRNA. Molecular human reproduction. 2011;17(1):63-70. doi: 10.1093/molehr/gaq073. PubMed PMID: 20843821.

A320. Nikiforovich GV, Frieden C. The search for local native-like nucleation centers in the unfolded state of beta -sheet proteins. Proceedings of the National Academy of Sciences of the United States of America. 2002;99(16):10388-93. doi: 10.1073/pnas.162362199. PubMed PMID: 12140369; PubMed Central PMCID: PMC124924.

A321. Ninomiya I, Osugi H, Fujimura T, Fushida S, Okamoto K, Maruzen S, et al. Thoracoscopic esophagectomy with extended lymph node dissection in the left lateral position: technical feasibility and oncologic outcomes.

Diseases of the esophagus : official journal of the International Society for Diseases of the Esophagus / ISDE. 2014;27(2):159-67. doi: 10.1111/dote.12071. PubMed PMID: 23551804.

A322. Nithsdale V, Davies J, Croucher P. Psychosis and the experience of employment. Journal of occupational rehabilitation. 2008;18(2):175-82. doi: 10.1007/s10926-008-9131-6. PubMed PMID: 18401690.

A323. Noel S, Dua J, Kaushal G. An 81 year old man with a blistering rash. Bmj. 2013;346:f522. doi: 10.1136/bmj.f522. PubMed PMID: 23369776.

A324. O'Shea B. Post-traumatic stress disorder: A review for the general psychiatrist. International journal of psychiatry in clinical practice. 2001;5(1):11-8. doi: 10.1080/136515001300225141. PubMed PMID: 24936990.

A325. Odum CU, Anorlu RI, Dim SI, Oyekan TO. Pregnancy outcome in HbSS-sickle cell disease in Lagos, Nigeria. West African journal of medicine. 2002;21(1):19-23. PubMed PMID: 12083064.

A326. Oehler DZ, Robert F, Mostefaoui S, Meibom A, Selo M, McKay DS. Chemical mapping of proterozoic organic matter at submicron spatial resolution. Astrobiology. 2006;6(6):838-50. doi: 10.1089/ast.2006.6.838. PubMed PMID: 17155884.

A327. Okumura M, Iwata K, Yasuda K, Inoue K, Shinoda M, Honda K, et al. Alternation of gene expression in trigeminal ganglion neurons following complete Freund's adjuvant or capsaicin injection into the rat face. Journal

of molecular neuroscience : MN. 2010;42(2):200-9. doi: 10.1007/s12031-010-9348-7. PubMed PMID: 20349343.

A328. Otis-Green S, Ferrell B, Borneman T, Puchalski C, Uman G, Garcia A. Integrating spiritual care within palliative care: an overview of nine demonstration projects. Journal of palliative medicine. 2012;15(2):154-62. doi: 10.1089/jpm.2011.0211. PubMed PMID: 22304654.

A329. Partridge AH, Elmore JG, Saslow D, McCaskill-Stevens W, Schnitt SJ. Challenges in ductal carcinoma in situ risk communication and decision-making: report from an American Cancer Society and National Cancer Institute workshop. CA: a cancer journal for clinicians. 2012;62(3):203-10. doi: 10.3322/caac.21140. PubMed PMID: 22488610.

A330. Patel MR, Dai D, Hernandez AF, Douglas PS, Messenger J, Garratt KN, et al. Prevalence and predictors of nonobstructive coronary artery disease identified with coronary angiography in contemporary clinical practice. American heart journal. 2014;167(6):846-52 e2. doi: 10.1016/j.ahj.2014.03.001. PubMed PMID: 24890534.

A331. Pavlikakis GE, Tsihrintzis VA. A quantitative method for accounting human opinion, preferences and perceptions in ecosystem management. Journal of environmental management. 2003;68(2):193-205. PubMed PMID: 12781759.

A332. Pawlik TM, Perry A, Strom EA, Babiera GV, Buchholz TA, Singletary E, et al. Potential applicability of balloon catheter-based accelerated partial

breast irradiation after conservative surgery for breast carcinoma. Cancer. 2004;100(3):490-8. doi: 10.1002/cncr.11939. PubMed PMID: 14745864.

A333. Pedraza-Chaverri J, Gil-Ortiz M, Albarran G, Barbachano-Esparza L, Menjivar M, Medina-Campos ON. Garlic's ability to prevent in vitro Cu2+-induced lipoprotein oxidation in human serum is preserved in heated garlic: effect unrelated to Cu2+-chelation. Nutrition journal. 2004;3:10. doi: 10.1186/1475-2891-3-10. PubMed PMID: 15341661; PubMed Central PMCID: PMC519022.

A334. Pellegrini N, Serafini M, Colombi B, Del Rio D, Salvatore S, Bianchi M, et al. Total antioxidant capacity of plant foods, beverages and oils consumed in Italy assessed by three different in vitro assays. The Journal of nutrition. 2003;133(9):2812-9. PubMed PMID: 12949370.

A335. Pencina MJ. Caution is needed in the interpretation of added value of biomarkers analyzed in matched case control studies. Clinical chemistry. 2012;58(8):1176-8. doi: 10.1373/clinchem.2012.188383. PubMed PMID: 22730448.

A336. Peng X, Li P, Shi Y. Synthesis of (+)-ambrisentan via chiral ketone-catalyzed asymmetric epoxidation. The Journal of organic chemistry. 2012;77(1):701-3. doi: 10.1021/jo201927m. PubMed PMID: 22098088.

A337. Penner GB, Steele MA, Aschenbach JR, McBride BW. Ruminant Nutrition Symposium: Molecular adaptation of ruminal epithelia to highly fermentable diets. Journal of animal science. 2011;89(4):1108-19. doi: 10.2527/jas.2010-3378. PubMed PMID: 20971890.

A338. Peppe A, Pierantozzi M, Altibrandi MG, Giacomini P, Stefani A, Bassi A, et al. Bilateral GPi DBS is useful to reduce abnormal involuntary movements in advanced Parkinson's disease patients, but its action is related to modality and site of stimulation. European journal of neurology : the official journal of the European Federation of Neurological Societies. 2001;8(6):579-86. PubMed PMID: 11784342.

A339. Peres WA, Lento DF, Baluz K, Ramalho A. Phase angle as a nutritional evaluation tool in all stages of chronic liver disease. Nutricion hospitalaria. 2012;27(6):2072-8. doi: 10.3305/nh.2012.27.6.6015. PubMed PMID: 23588459.

A340. Peters M, Manning JT, Reimers S. The effects of sex, sexual orientation, and digit ratio (2D:4D) on mental rotation performance. Archives of sexual behavior. 2007;36(2):251-60. doi: 10.1007/s10508-006-9166-8. PubMed PMID: 17394056.

A341. Pfeifer GP, Besaratinia A. UV wavelength-dependent DNA damage and human non-melanoma and melanoma skin cancer. Photochemical & photobiological sciences : Official journal of the European Photochemistry Association and the European Society for Photobiology. 2012;11(1):90-7. doi: 10.1039/c1pp05144j. PubMed PMID: 21804977; PubMed Central PMCID: PMC3289542.

A342. Philopoulos D. Application of criteria developed by the Task Force on Neonatal Encephalopathy and Cerebral Palsy to acutely asphyxiated

neonates. Obstetrics and gynecology. 2012;119(5):1056; author reply -7. doi: 10.1097/AOG.0b013e318254268a. PubMed PMID: 22525923.

A343. Pioli PA, Weaver LK, Schaefer TM, Wright JA, Wira CR, Guyre PM. Lipopolysaccharide-induced IL-1 beta production by human uterine macrophages up-regulates uterine epithelial cell expression of human beta-defensin 2. Journal of immunology. 2006;176(11):6647-55. PubMed PMID: 16709823.

A344. Poland D. Intermediates in the melting transitions of aluminum nanoclusters. The Journal of chemical physics. 2007;126(5):054507. doi: 10.1063/1.2432121. PubMed PMID: 17302485.

A345. Poles MA, Fuerst M, McGowan I, Elliott J, Rezaei A, Mark D, et al. HIV-related diarrhea is multifactorial and fat malabsorption is commonly present, independent of HAART. The American journal of gastroenterology. 2001;96(6):1831-7. doi: 10.1111/j.1572-0241.2001.03879.x. PubMed PMID: 11421246.

A346. Porel M, Chuang CH, Burda C, Ramamurthy V. Ultrafast photoinduced electron transfer between an incarcerated donor and a free acceptor in aqueous solution. Journal of the American Chemical Society. 2012;134(36):14718-21. doi: 10.1021/ja3067594. PubMed PMID: 22931120.

A347. Prugh J, Zeppieri G, Jr., George SZ. Impact of psychosocial factors, pain, and functional limitations on throwing athletes who return to sport following elbow injuries: a case series. Physiotherapy theory and practice.

2012;28(8):633-40. doi: 10.3109/09593985.2012.666632. PubMed PMID: 22432794.

A348. Puckett RL, Moore SA, Winder TL, Willer T, Romansky SG, Covault KK, et al. Further evidence of Fukutin mutations as a cause of childhood onset limb-girdle muscular dystrophy without mental retardation. Neuromuscular disorders : NMD. 2009;19(5):352-6. doi: 10.1016/j.nmd.2009.03.001. PubMed PMID: 19342235; PubMed Central PMCID: PMC2698593.

A349. Puente A, Diaz RJ. Is it possible to assess the ecological status of highly stressed natural estuarine environments using macroinvertebrates indices? Marine pollution bulletin. 2008;56(11):1880-9. doi: 10.1016/j.marpolbul.2008.07.016. PubMed PMID: 18790507.

A350. Qian X, Shen G, Wang Z, Guo C, Liu Y, Lei Z, et al. Co-composting of livestock manure with rice straw: characterization and establishment of maturity evaluation system. Waste management. 2014;34(2):530-5. doi: 10.1016/j.wasman.2013.10.007. PubMed PMID: 24188923.

A351. Qin T, Zajaczkowski W, Pisula W, Baumgarten M, Chen M, Gao M, et al. Tailored donor-acceptor polymers with an A-D1-A-D2 structure: controlling intermolecular interactions to enable enhanced polymer photovoltaic devices. Journal of the American Chemical Society. 2014;136(16):6049-55. doi: 10.1021/ja500935d. PubMed PMID: 24697300.

A352. Qiu S, Aldinger KA, Levitt P. Modeling of autism genetic variations in mice: focusing on synaptic and microcircuit dysfunctions. Developmental

neuroscience. 2012;34(2-3):88-100. doi: 10.1159/000336644. PubMed PMID: 22572629.

A353. Quirion E. Filgrastim and pegfilgrastim use in patients with neutropenia. Clinical journal of oncology nursing. 2009;13(3):324-8. doi: 10.1188/09.CJON.324-328. PubMed PMID: 19502191.

A354. Rahel FJ. Homogenization of fish faunas across the United States. Science. 2000;288(5467):854-6. PubMed PMID: 10797007.

A355. Rahman Z, Zidan AS, Khan MA. Non-destructive methods of characterization of risperidone solid lipid nanoparticles. European journal of pharmaceutics and biopharmaceutics : official journal of Arbeitsgemeinschaft fur Pharmazeutische Verfahrenstechnik eV. 2010;76(1):127-37. doi: 10.1016/j.ejpb.2010.05.003. PubMed PMID: 20470882.

A356. Rajanikanth BR, Rao KS, Sharma SM, Rajendra Prasad B. Assessment of deformities of the lip and nose in cleft lip alveolus and palate patients by a rating scale. Journal of maxillofacial and oral surgery. 2012;11(1):38-46. doi: 10.1007/s12663-011-0298-6. PubMed PMID: 23449402; PubMed Central PMCID: PMC3319831.

A357. Ram CV, Fenves A. Clinical pharmacology of antihypertensive drugs. Cardiology clinics. 2002;20(2):265-80. PubMed PMID: 12119800.

A358. Reeuwijk KG, de Wind A, Westerman MJ, Ybema JF, van der Beek AJ, Geuskens GA. 'All those things together made me retire': qualitative study on early retirement among Dutch employees. BMC public health.

2013;13(1):516. doi: 10.1186/1471-2458-13-516. PubMed PMID: 23714371; PubMed Central PMCID: PMC3674915.

A359. Ren Q, Zhang Z, Zhao J. Effect on information transfer of synaptic pruning driven by spike-timing-dependent plasticity. Physical review E, Statistical, nonlinear, and soft matter physics. 2012;85(2 Pt 1):022901. PubMed PMID: 22463266.

A360. Ricci A, Marella GL, Apostol MA. A new experimental approach to computer-aided face/skull identification in forensic anthropology. The American journal of forensic medicine and pathology. 2006;27(1):46-9. doi: 10.1097/01.paf.0000202809.96283.88. PubMed PMID: 16501348.

A361. Riordan T, Cartwright K, Cunningham R, Logan M, Wright P. A survey of time management and particular tasks undertaken by consultant microbiologists in the UK. Journal of clinical pathology. 2007;60(5):540-4. doi: 10.1136/jcp.2005.033985. PubMed PMID: 16714398; PubMed Central PMCID: PMC1994525.

A362. Ritz BW. Supplementation with active hexose correlated compound increases survival following infectious challenge in mice. Nutrition reviews. 2008;66(9):526-31. doi: 10.1111/j.1753-4887.2008.00085.x. PubMed PMID: 18752476.

A363. Roberts DC, Gabriele A, Zimmer BA. Conflation of cocaine seeking and cocaine taking responses in IV self-administration experiments in rats: methodological and interpretational considerations. Neuroscience and biobehavioral reviews. 2013;37(9 Pt A):2026-36. doi:

10.1016/j.neubiorev.2013.04.017. PubMed PMID: 23669047; PubMed Central PMCID: PMC3838507.

A364. Roberts H. Experiences in rural Kenya: Addressing lack of knowledge. Community eye health / International Centre for Eye Health. 2012;25(78):28. PubMed PMID: 23139447; PubMed Central PMCID: PMC3483606.

A365. Rojas DB, de Andrade RB, Gemelli T, Oliveira LS, Campos AG, Dutra-Filho CS, et al. Effect of histidine administration to female rats during pregnancy and lactation on enzymes activity of phosphoryltransfer network in cerebral cortex and hippocampus of the offspring. Metabolic brain disease. 2012;27(4):595-603. doi: 10.1007/s11011-012-9319-5. PubMed PMID: 22638695.

A366. Roner MR, Sprayberry J, Spinks M, Dhanji S. Antiviral activity obtained from aqueous extracts of the Chilean soapbark tree (Quillaja saponaria Molina). The Journal of general virology. 2007;88(Pt 1):275-85. doi: 10.1099/vir.0.82321-0. PubMed PMID: 17170461.

A367. Rosen HR, Ribeiro RR, Weinberger L, Wolf S, Chung M, Gretch DR, et al. Early hepatitis C viral kinetics correlate with long-term outcome in patients receiving high dose induction followed by combination interferon and ribavirin therapy. Journal of hepatology. 2002;37(1):124-30. PubMed PMID: 12076871.

A368. Rosen SD, Paulesu E, Wise RJ, Camici PG. Central neural contribution to the perception of chest pain in cardiac syndrome X. Heart.

2002;87(6):513-9. PubMed PMID: 12010930; PubMed Central PMCID: PMC1767119.

A369. Rowe LA, Geddie ML, Alexander OB, Matsumura I. A comparison of directed evolution approaches using the beta-glucuronidase model system. Journal of molecular biology. 2003;332(4):851-60. PubMed PMID: 12972256.

A370. Ruijs CD, Kerkhof AJ, van der Wal G, Onwuteaka-Philipsen BD. Symptoms, unbearability and the nature of suffering in terminal cancer patients dying at home: a prospective primary care study. BMC family practice. 2013;14:201. doi: 10.1186/1471-2296-14-201. PubMed PMID: 24373224; PubMed Central PMCID: PMC3877870.

A371. Russold M, Jarvis JC. Implantable stimulator featuring multiple programs, adjustable stimulation amplitude and bi-directional communication for implantation in mice. Medical & biological engineering & computing. 2007;45(7):695-9. doi: 10.1007/s11517-007-0190-1. PubMed PMID: 17541669.

A372. Sabadini E, Egidio Fdo C, Cosgrove T. More on polypseudorotaxanes formed between poly(ethylene glycol) and alpha-cyclodextrin. Langmuir : the ACS journal of surfaces and colloids. 2013;29(15):4664-9. doi: 10.1021/la304910v. PubMed PMID: 23510502.

A373. Sadhu S, Patra A. Donor-acceptor systems: energy transfer from CdS quantum dots/rods to Nile Red dye. Chemphyschem : a European journal

of chemical physics and physical chemistry. 2008;9(14):2052-8. doi: 10.1002/cphc.200800298. PubMed PMID: 18756556.

A374. Sahasrabuddhe VV, Gravitt PE, Dunn ST, Brown D, Allen RA, Eby YJ, et al. Comparison of human papillomavirus detections in urine, vulvar, and cervical samples from women attending a colposcopy clinic. Journal of clinical microbiology. 2014;52(1):187-92. doi: 10.1128/JCM.01623-13. PubMed PMID: 24197879; PubMed Central PMCID: PMC3911475.

A375. Sahtout AH, Hassan MD, Shariff M. DNA fragmentation, an indicator of apoptosis, in cultured black tiger shrimp Penaeus monodon infected with white spot syndrome virus (WSSV). Diseases of aquatic organisms. 2001;44(2):155-9. doi: 10.3354/dao044155. PubMed PMID: 11324818.

A376. Salamova A, Hermanson MH, Hites RA. Organophosphate and halogenated flame retardants in atmospheric particles from a European arctic site. Environmental science & technology. 2014;48(11):6133-40. doi: 10.1021/es500911d. PubMed PMID: 24848787.

A377. Salem L, Anaya DA, Flum DR. Temporal changes in the management of diverticulitis. The Journal of surgical research. 2005;124(2):318-23. doi: 10.1016/j.jss.2004.11.005. PubMed PMID: 15820264.

A378. Sanchez M, Drechsler M, Stark H, Lipps G. DNA translocation activity of the multifunctional replication protein ORF904 from the archaeal plasmid pRN1. Nucleic acids research. 2009;37(20):6831-48. doi: 10.1093/nar/gkp742. PubMed PMID: 19762479; PubMed Central PMCID: PMC2777425.

A379. Sanson M, Laigle-Donadey F, Benouaich-Amiel A. Molecular changes in brain tumors: prognostic and therapeutic impact. Current opinion in oncology. 2006;18(6):623-30. doi: 10.1097/01.cco.0000245322.11787.72. PubMed PMID: 16988585.

A380. Santana I, Duro D, Freitas S, Alves L, Simoes MR. The Clock Drawing Test: Portuguese norms, by age and education, for three different scoring systems. Archives of clinical neuropsychology : the official journal of the National Academy of Neuropsychologists. 2013;28(4):375-87. doi: 10.1093/arclin/act016. PubMed PMID: 23518874.

A381. Sari I, Uslu N, Gorgulu S, Nurkalem Z, Eren M. Inferior myocardial infarction and extensive atherosclerosis in a patient with double right coronary artery. International journal of cardiology. 2006;111(2):321-3. doi: 10.1016/j.ijcard.2005.11.028. PubMed PMID: 16413074.

A382. Sasaki T, Nagata Y, Sugiyama K. Nasolacrimal duct obstruction classified by dacryoendoscopy and treated with inferior meatal dacryorhinotomy. Part I: Positional diagnosis of primary nasolacrimal duct obstruction with dacryoendoscope. American journal of ophthalmology. 2005;140(6):1065-9. doi: 10.1016/j.ajo.2005.07.038. PubMed PMID: 16376652.

A383. Sayer EJ. Using experimental manipulation to assess the roles of leaf litter in the functioning of forest ecosystems. Biological reviews of the Cambridge Philosophical Society. 2006;81(1):1-31. doi: 10.1017/S1464793105006846. PubMed PMID: 16460580.

A384. Schaefer SA, Buitrago-Suarez UA. Odontode morphology and skin surface features of Andean astroblepid catfishes (Siluriformes, Astroblepidae). Journal of morphology. 2002;254(2):139-48. doi: 10.1002/jmor.10024. PubMed PMID: 12353298.

A385. Scharer MA, Eliot AC, Grutter MG, Capitani G. Structural basis for reduced activity of 1-aminocyclopropane-1-carboxylate synthase affected by a mutation linked to andromonoecy. FEBS letters. 2011;585(1):111-4. doi: 10.1016/j.febslet.2010.11.013. PubMed PMID: 21075107.

A386. Schatten H. Introduction to stem cell special section. Microscopy and microanalysis : the official journal of Microscopy Society of America, Microbeam Analysis Society, Microscopical Society of Canada. 2011;17(4):473. doi: 10.1017/S1431927611011998. PubMed PMID: 21740615.

A387. Schley M, Fritzsche S, Lonnecke P, Hey-Hawkins E. Soluble monometallic salen complexes derived from O-functionalised diamines as metalloligands for the synthesis of heterobimetallic complexes. Dalton transactions. 2010;39(17):4090-106. doi: 10.1039/b920706f. PubMed PMID: 20390172.

A388. Schmid-Grendelmeier P, Mahe A, Ponnighaus JM, Welsh O, Stingl P, Leppard B. Tropical dermatology. Part I. Journal of the American Academy of Dermatology. 2002;46(4):571-83. PubMed PMID: 11907511.

A389. Schopfer D, Patel V, Chiang J, Kao J. Hospitals with percutaneous coronary intervention capability have greater adherence to established

myocardial infarction guidelines. Hospital practice. 2010;38(2):9-13. PubMed PMID: 20469608.

A390. Schubert C, Rudolf M, Guldi DM, Takano Y, Mizorogi N, Herranz MA, et al. Rates and energetics of intramolecular electron transfer processes in conjugated metallofullerenes. Philosophical transactions Series A, Mathematical, physical, and engineering sciences. 2013;371(1998):20120490. doi: 10.1098/rsta.2012.0490.

A391. Schwab JO, Eichner G, Veit G, Schmitt H, Lewalter T, Luderitz B. Influence of basic heart rate and sex on heart rate turbulence in healthy subjects. Pacing and clinical electrophysiology : PACE. 2004;27(12):1625-31. doi: 10.1111/j.1540-8159.2004.00695.x. PubMed PMID: 15613125.

A392. Schwenke DW, Partridge H. Vibrational energy levels for CH4 from an ab initio potential. Spectrochimica acta Part A, Molecular and biomolecular spectroscopy. 2001;57(4):887-95. PubMed PMID: 11345261.

A393. Scoles DR, Pflieger LT, Thai KK, Hansen ST, Dansithong W, Pulst SM. ETS1 regulates the expression of ATXN2. Human molecular genetics. 2012;21(23):5048-65. doi: 10.1093/hmg/dds349. PubMed PMID: 22914732; PubMed Central PMCID: PMC3490512.

A394. Scott JW, Acevedo HP, Sherrill L, Phan M. Responses of the rat olfactory epithelium to retronasal air flow. Journal of neurophysiology. 2007;97(3):1941-50. doi: 10.1152/jn.01305.2006. PubMed PMID: 17215498; PubMed Central PMCID: PMC2225990.

A395. Sen P, Darabi S. Compressive rendering: a rendering application of compressed sensing. IEEE transactions on visualization and computer graphics. 2011;17(4):487-99. doi: 10.1109/TVCG.2010.46. PubMed PMID: 21311092.

A396. Sethi SK. Invited manuscript poster on renal-related education American Society of Nephrology, Nov. 16-21, 2010. E-pediatric nephrology in India. Renal failure. 2011;33(7):751-2. doi: 10.3109/0886022X.2011.589953. PubMed PMID: 21787166.

A397. Shafrir E, Malecki M, Schernthaner G, Kiljanski J. Treatment of type 2 diabetes mellitus. Introduction. Diabetes research and clinical practice. 2008;82 Suppl 2:S81-2. doi: 10.1016/j.diabres.2008.09.019. PubMed PMID: 19019474.

A398. Shah KH, Verma RJ. Butyl p-hydroxybenzoic acid induces oxidative stress in mice liver--an in vivo study. Acta poloniae pharmaceutica. 2011;68(6):875-9. PubMed PMID: 22125952.

A399. Shah NH, Tenenbaum JD. The coming age of data-driven medicine: translational bioinformatics' next frontier. Journal of the American Medical Informatics Association : JAMIA. 2012;19(e1):e2-4. doi: 10.1136/amiajnl-2012-000969. PubMed PMID: 22718035; PubMed Central PMCID: PMC3392866.

A400. Shahin AY, Ismail AM, Zahran KM, Makhlouf AM. Adding phytoestrogens to clomiphene induction in unexplained infertility patients--a randomized

trial. Reproductive biomedicine online. 2008;16(4):580-8. PubMed PMID: 18413068.

A401. Shalhoub J, Monaco C, Owen DR, Gauthier T, Thapar A, Leen EL, et al. Late-phase contrast-enhanced ultrasound reflects biological features of instability in human carotid atherosclerosis. Stroke; a journal of cerebral circulation. 2011;42(12):3634-6. doi: 10.1161/STROKEAHA.111.631200. PubMed PMID: 21960570.

A402. Shek TW. Chondroblastoma of temporal bone. The American journal of otology. 2000;21(4):597-8. PubMed PMID: 10912709.

A403. Shi DD, Trigo FF, Semmelhack MF, Wang SS. Synthesis and biological evaluation of bis-CNB-GABA, a photoactivatable neurotransmitter with low receptor interference and chemical two-photon uncaging properties. Journal of the American Chemical Society. 2014;136(5):1976-81. doi: 10.1021/ja411082f. PubMed PMID: 24422544; PubMed Central PMCID: PMC3985807.

A404. Shintani M, Takahashi Y, Tokumaru H, Kadota K, Hara H, Miyakoshi M, et al. Response of the Pseudomonas host chromosomal transcriptome to carriage of the IncP-7 plasmid pCAR1. Environmental microbiology. 2010;12(6):1413-26. doi: 10.1111/j.1462-2920.2009.02110.x. PubMed PMID: 19930443.

A405. Shufelt CL, Merz CN, Prentice RL, Pettinger MB, Rossouw JE, Aroda VR, et al. Hormone therapy dose, formulation, route of delivery, and risk of cardiovascular events in women: findings from the Women's Health

Initiative Observational Study. Menopause. 2014;21(3):260-6. doi: 10.1097/GME.0b013e31829a64f9. PubMed PMID: 24045672; PubMed Central PMCID: PMC3872264.

A406. Siam EM. Office microlaparoscopic intrafallopian transfer of day one zygote versus day three embryo transfer after previous failed ICSI trials. African journal of reproductive health. 2011;15(2):153-61. PubMed PMID: 22590901.

A407. Simpson K, Leyendecker P, Hopp M, Muller-Lissner S, Lowenstein O, De Andres J, et al. Fixed-ratio combination oxycodone/naloxone compared with oxycodone alone for the relief of opioid-induced constipation in moderate-to-severe noncancer pain. Current medical research and opinion. 2008;24(12):3503-12. doi: 10.1185/03007990802584454. PubMed PMID: 19032132.

A408. Sinkin RA, Fisher SG, Dozier A, Dye TD. Effect of managed care on perinatal transports for the publicly funded in upstate New York. Journal of perinatology : official journal of the California Perinatal Association. 2005;25(2):79-85. doi: 10.1038/sj.jp.7211213. PubMed PMID: 15496969.

A409. Soda M, Ishikura T, Nakamura H, Wakabayashi Y, Kimura T. Magnetic ordering in relation to the room-temperature magnetoelectric effect of Sr3Co2Fe24O41. Physical review letters. 2011;106(8):087201. PubMed PMID: 21405595.

A410. Solcher SC, Langhofer S, Layes D, Dryer LA, Yelton S, Long C. Fistulae are first at Kansas dialysis services. Nephrology nursing journal : journal

of the American Nephrology Nurses' Association. 2006;33(4):449-53, 61. PubMed PMID: 17002004.

A411. Song DK, Im YB, Jung JS, Cho J, Suh HW, Kim YH. Central beta-amyloid peptide-induced peripheral interleukin-6 responses in mice. Journal of neurochemistry. 2001;76(5):1326-35. PubMed PMID: 11238717.

A412. Sorensen B, Nielsen GL, Ebbesen LS, Ingerslev J. Feasibility of LMW heparin prophylaxis for management of thrombophilia investigation in patients on oral anticoagulation therapy. Thrombosis and haemostasis. 2001;85(3):563-4. PubMed PMID: 11307834.

A413. Soundy A, Taylor A, Faulkner G, Rowlands A. Psychometric properties of the 7-Day Physical Activity Recall questionnaire in individuals with severe mental illness. Archives of psychiatric nursing. 2007;21(6):309-16. doi: 10.1016/j.apnu.2007.03.001. PubMed PMID: 18037441.

A414. Spethmann S, Fischer C, Wagener C, Streichert T, Tschentscher P. Nucleic acids from intact epithelial cells as a target for stool-based molecular diagnosis of colorectal cancer. International journal of molecular medicine. 2004;13(3):451-4. PubMed PMID: 14767578.

A415. Sra J, Narayan G, Krum D, Akhtar M. Registration of 3D computed tomographic images with interventional systems: implications for catheter ablation of atrial fibrillation. Journal of interventional cardiac electrophysiology : an international journal of arrhythmias and pacing. 2006;16(3):141-8. doi: 10.1007/s10840-006-9030-8. PubMed PMID: 17139556.

A416. Stamatatos TC, Christou G. Azide groups in higher oxidation state manganese cluster chemistry: from structural aesthetics to single-molecule magnets. Inorganic chemistry. 2009;48(8):3308-22. doi: 10.1021/ic801217j. PubMed PMID: 19364123.

A417. Stephan CN, Winburn AP, Christensen AF, Tyrrell AJ. Skeletal identification by radiographic comparison: blind tests of a morphoscopic method using antemortem chest radiographs. Journal of forensic sciences. 2011;56(2):320-32. doi: 10.1111/j.1556-4029.2010.01673.x. PubMed PMID: 21306373.

A418. Steudel R, Steudel Y, Wong MW. Complexation of the vulcanization accelerator tetramethylthiuram disulfide and related molecules with zinc compounds including zinc oxide clusters (Zn4O4). Chemistry. 2008;14(3):919-32. doi: 10.1002/chem.200701089. PubMed PMID: 17994599.

A419. Stevenson JG. Overcoming inertia: achieving more-rapid transformation of pharmacy practice. American journal of health-system pharmacy : AJHP : official journal of the American Society of Health-System Pharmacists. 2011;68(7):604-12. doi: 10.2146/ajhp100640. PubMed PMID: 21411802.

A420. Stoddart B, Collyns T, Denton M. Fusidic acid cream for impetigo. Problem may be clinically important. Bmj. 2002;324(7350):1394. PubMed PMID: 12053929.

A421. Stone AF, Mendall MA, Kaski JC, Edger TM, Risley P, Poloniecki J, et al. Effect of treatment for Chlamydia pneumoniae and Helicobacter pylori on

markers of inflammation and cardiac events in patients with acute coronary syndromes: South Thames Trial of Antibiotics in Myocardial Infarction and Unstable Angina (STAMINA). Circulation. 2002;106(10):1219-23. PubMed PMID: 12208796.

A422. Streetman DD, Khanderia U. Diagnosis and treatment of Graves disease. The Annals of pharmacotherapy. 2003;37(7-8):1100-9. PubMed PMID: 12841824.

A423. Struyf S, Stoops G, Van Coillie E, Gouwy M, Schutyser E, Lenaerts JP, et al. Gene cloning of a new plasma CC chemokine, activating and attracting myeloid cells in synergy with other chemoattractants. Biochemistry. 2001;40(39):11715-22. PubMed PMID: 11570872.

A424. Sudrania OP. Kurt Semm: A laparoscopic crusader. Journal of minimal access surgery. 2007;3(3):115. doi: 10.4103/0972-9941.37197. PubMed PMID: 19789670; PubMed Central PMCID: PMC2749188.

A425. Sumner JA, Pietrzak RH, Aiello AE, Uddin M, Wildman DE, Galea S, et al. Further Support for an Association between the Memory-Related Gene WWC1 and Posttraumatic Stress Disorder: Results from the Detroit Neighborhood Health Study. Biological psychiatry. 2014. doi: 10.1016/j.biopsych.2014.03.033. PubMed PMID: 24947539.

A426. Suska A, Miguel-Aliaga I, Thor S. Segment-specific generation of Drosophila Capability neuropeptide neurons by multi-faceted Hox cues. Developmental biology. 2011;353(1):72-80. doi:

10.1016/j.ydbio.2011.02.015. PubMed PMID: 21354130; PubMed Central PMCID: PMC3094758.

A427. Suzuki R, Irisawa A, Bhutani MS, Takagi T, Ikeda T, Hikichi T, et al. Ultrathin endoscope-guided trangastric nasocystic irrigation tube placement to manage paracolic gutter extension of pancreatic necrosis. Gastrointestinal endoscopy. 2012;76(2):457-9. doi: 10.1016/j.gie.2011.07.065. PubMed PMID: 21963068.

A428. Szemenyei H, Hannon M, Long JA. TOPLESS mediates auxin-dependent transcriptional repression during Arabidopsis embryogenesis. Science. 2008;319(5868):1384-6. doi: 10.1126/science.1151461. PubMed PMID: 18258861.

A429. Takayama S, Watanabe M, Kusuyama H, Nagase S, Seki T, Nakazawa T, et al. Evaluation of the effects of acupuncture on blood flow in humans with ultrasound color Doppler imaging. Evidence-based complementary and alternative medicine : eCAM. 2012;2012:513638. doi: 10.1155/2012/513638. PubMed PMID: 22778772; PubMed Central PMCID: PMC3388479.

A430. Tang ZQ, Lu Y. Development of GPCR modulation of GABAergic transmission in chicken nucleus laminaris neurons. PloS one. 2012;7(4):e35831. doi: 10.1371/journal.pone.0035831. PubMed PMID: 22545142; PubMed Central PMCID: PMC3335798.

A431. Tarrant RD, Velez-Suberbie ML, Tait AS, Smales CM, Bracewell DG. Host cell protein adsorption characteristics during protein A chromatography.

Biotechnology progress. 2012;28(4):1037-44. doi: 10.1002/btpr.1581. PubMed PMID: 22736545.

A432. Ter Veld MG, Zawadzka E, Rietjens IM, Murk AJ. Estrogenicity of food-associated estrogenic compounds in the fetuses of female transgenic mice upon oral and IP maternal exposure. Reproductive toxicology. 2009;27(2):133-9. doi: 10.1016/j.reprotox.2009.01.010. PubMed PMID: 19429392.

A433. Terrosu G, Baccarani U, Bresadola V, Sistu MA, Uzzau A, Bresadola F. The impact of splenic weight on laparoscopic splenectomy for splenomegaly. Surgical endoscopy. 2002;16(1):103-7. doi: 10.1007/s00464-001-9045-2. PubMed PMID: 11961616.

A434. Thompson BT, Orme JF, Zheng H, Luckett PM, Truwit JD, Willson DF, et al. Multicenter validation of a computer-based clinical decision support tool for glucose control in adult and pediatric intensive care units. Journal of diabetes science and technology. 2008;2(3):357-68. PubMed PMID: 19885199; PubMed Central PMCID: PMC2769731.

A435. Timmer JR, van der Horst IC, de Luca G, Ottervanger JP, Hoorntje JC, de Boer MJ, et al. Comparison of myocardial perfusion after successful primary percutaneous coronary intervention in patients with ST-elevation myocardial infarction with versus without diabetes mellitus. The American journal of cardiology. 2005;95(11):1375-7. doi: 10.1016/j.amjcard.2005.01.088. PubMed PMID: 15904649.

A436. Tiwari L, Varghese RM, Puliyel JM. Computerised-hepatitis B-model is subject to processing axiom: garbage in, garbage out. Journal of hepatology. 2003;39(1):133; author reply 4-5. PubMed PMID: 12821058.

A437. Tondi P, Gerardino L, Santoliquido A, Pola R, Gabrielli M, Papaleo P, et al. Treatment of ischemic ulcers of the lower limbs with alprostadil (prostaglandin E1). Dermatologic surgery : official publication for American Society for Dermatologic Surgery [et al]. 2004;30(8):1113-7. doi: 10.1111/j.1524-4725.2004.30336.x. PubMed PMID: 15274701.

A438. Torralba AS, Yu K, Shen P, Oefner PJ, Ross J. Experimental test of a method for determining causal connectivities of species in reactions. Proceedings of the National Academy of Sciences of the United States of America. 2003;100(4):1494-8. doi: 10.1073/pnas.262790699. PubMed PMID: 12576555; PubMed Central PMCID: PMC149859.

A439. Trathnigg B, Ahmed H. Separation of all oligomers in polyethylene glycols and their monomethyl ethers by one-dimensional liquid chromatography. Analytical and bioanalytical chemistry. 2011;399(4):1535-45. doi: 10.1007/s00216-010-3951-x. PubMed PMID: 20614107.

A440. Traub RJ, Olsen PC, McDonald JC. The radiological properties of a novel lung tissue substitute. Radiation protection dosimetry. 2006;121(2):202-7. doi: 10.1093/rpd/nci371. PubMed PMID: 17142822.

A441. Trost SG, Fees BS, Haar SJ, Murray AD, Crowe LK. Identification and validity of accelerometer cut-points for toddlers. Obesity.

2012;20(11):2317-9. doi: 10.1038/oby.2011.364. PubMed PMID: 22173573.

A442. Trumble S. Teaching the teacher. The clinical teacher. 2013;10(4):207-8. doi: 10.1111/tct.12091. PubMed PMID: 23834563.

A443. Tsai CN, Chiang WC, Sun CL, Shao KT, Chen SY, Yeh SZ. Trophic size-structure of sailfish Istiophorus platypterus in eastern Taiwan estimated by stable isotope analysis. Journal of fish biology. 2014;84(2):354-71. doi: 10.1111/jfb.12290. PubMed PMID: 24447322.

A444. Tse HF, Wang Q, Yu CM, Ayers GM, Lau CP. Time course of recovery of left atrial mechanical dysfunction after cardioversion of spontaneous atrial fibrillation with the implantable atrial defibrillator. The American journal of cardiology. 2000;86(9):1023-5, A10. PubMed PMID: 11053721.

A445. Tseng SH, Hayakawa C, Tromberg BJ, Spanier J, Durkin AJ. Quantitative spectroscopy of superficial turbid media. Optics letters. 2005;30(23):3165-7. PubMed PMID: 16350274.

A446. Tsuda E, Minami N, Kobayashi J, Fukaya T, Nozaki H, Noritake K, et al. Acute myocardial infarction after Kawasaki disease in an infant: treatment with coronary artery bypass grafting. Pediatrics international : official journal of the Japan Pediatric Society. 2009;51(3):421-4. doi: 10.1111/j.1442-200X.2009.02830.x. PubMed PMID: 19500286.

A447. Tsukada H, Sato K, Fukumoto D, Kakiuchi T. Evaluation of D-isomers of O-18F-fluoromethyl, O-18F-fluoroethyl and O-18F-fluoropropyl tyrosine as tumour imaging agents in mice. European journal of nuclear medicine and

molecular imaging. 2006;33(9):1017-24. doi: 10.1007/s00259-006-0076-8. PubMed PMID: 16699766.

A448. Turlure F, Devroe E, Silver PA, Engelman A. Human cell proteins and human immunodeficiency virus DNA integration. Frontiers in bioscience : a journal and virtual library. 2004;9:3187-208. PubMed PMID: 15353349.

A449. Turner CJ, Van Lancker H. A prepatellar Morel-Lavallee lesion in a pedestrian vs automobile collision. The American journal of emergency medicine. 2014;32(1):109 e3-4. doi: 10.1016/j.ajem.2013.08.023. PubMed PMID: 24075804.

A450. Uehara A, Sugawara Y, Kurata S, Fujimoto Y, Fukase K, Kusumoto S, et al. Chemically synthesized pathogen-associated molecular patterns increase the expression of peptidoglycan recognition proteins via toll-like receptors, NOD1 and NOD2 in human oral epithelial cells. Cellular microbiology. 2005;7(5):675-86. doi: 10.1111/j.1462-5822.2004.00500.x. PubMed PMID: 15839897.

A451. Umaki TM, Umaki MR, Cobb CM. The psychology of patient compliance: a focused review of the literature. Journal of periodontology. 2012;83(4):395-400. doi: 10.1902/jop.2011.110344. PubMed PMID: 21819248.

A452. van der Heyde Y. Medical responsibility at inquests. South African medical journal = Suid-Afrikaanse tydskrif vir geneeskunde. 2008;98(11):820, 2. PubMed PMID: 19177880.

A453. van Keulen CJ, van den Akker E, van den Berg FG, Pals G, Rauwerda JA. The role of type III collagen in family members of patients with abdominal aortic aneurysms. European journal of vascular and endovascular surgery : the official journal of the European Society for Vascular Surgery. 2000;20(4):379-85. doi: 10.1053/ejvs.2000.1193. PubMed PMID: 11035971.

A454. Vanchieri C. Texas case raises questions about Hodgkin lymphoma treatment in children. Journal of the National Cancer Institute. 2006;98(1):6-8. doi: 10.1093/jnci/djj027. PubMed PMID: 16391363.

A455. Vaughn J, Nayar AC, Srichai MB. Right coronary artery aneurysm with aneurysmal dilation and thrombosis of the sinoatrial nodal branch mimicking a right atrial mass. Journal of cardiovascular computed tomography. 2014;8(1):85-7. doi: 10.1016/j.jcct.2013.12.015. PubMed PMID: 24582048.

A456. Vidyashankar S, Sambaiah K, Srinivasan K. Effect of dietary garlic and onion on biliary proteins and lipid peroxidation which influence cholesterol nucleation in bile. Steroids. 2010;75(3):272-81. doi: 10.1016/j.steroids.2010.01.003. PubMed PMID: 20079366.

A457. Viola G, Dall'Acqua F, Gabellini N, Moro S, Vedaldi D, Ihmels H. Indolo[2,3-b]-quinolizinium bromide: an efficient intercalator with DNA-photodamaging properties. Chembiochem : a European journal of chemical biology. 2002;3(6):550-8. doi: 10.1002/1439-

7633(20020603)3:6<550::AID-CBIC550>3.0.CO;2-Z. PubMed PMID: 12325011.

A458. Vision in Preschoolers Study G. Does assessing eye alignment along with refractive error or visual acuity increase sensitivity for detection of strabismus in preschool vision screening? Investigative ophthalmology & visual science. 2007;48(7):3115-25. doi: 10.1167/iovs.06-1009. PubMed PMID: 17591881; PubMed Central PMCID: PMC2140241.

A459. Vlieghe P, Bihel F, Clerc T, Pannecouque C, Witvrouw M, De Clercq E, et al. New 3'-azido-3'-deoxythymidin-5'-yl O-(omega-hydroxyalkyl) carbonate prodrugs: synthesis and anti-HIV evaluation. Journal of medicinal chemistry. 2001;44(5):777-86. PubMed PMID: 11262088.

A460. Vogel CL, Tan-Chiu E. Trastuzumab plus chemotherapy: convincing survival benefit or not? Journal of clinical oncology : official journal of the American Society of Clinical Oncology. 2005;23(19):4247-50. doi: 10.1200/JCO.2005.12.903. PubMed PMID: 15911863.

A461. Walker M, Tschanz C. Stories are like water: an academic writing workshop for nurses. Creative nursing. 2013;19(2):81-5. PubMed PMID: 23798245.

A462. Walker RH, Friedman J, Wiener J, Hobler R, Gwinn-Hardy K, Adam A, et al. A family with a tau P301L mutation presenting with parkinsonism. Parkinsonism & related disorders. 2002;9(2):121-3. PubMed PMID: 12473404.

A463. Wallace K, Marek CJ, Currie RA, Wright MC. Exocrine pancreas trans-differentiation to hepatocytes--a physiological response to elevated glucocorticoid in vivo. The Journal of steroid biochemistry and molecular biology. 2009;116(1-2):76-85. doi: 10.1016/j.jsbmb.2009.05.002. PubMed PMID: 19446026.

A464. Wanders RJ, Jansen GA, Skjeldal OH. Refsum disease, peroxisomes and phytanic acid oxidation: a review. Journal of neuropathology and experimental neurology. 2001;60(11):1021-31. PubMed PMID: 11706932.

A465. Wang C, Kim T, Gao D, Vaglenov A, Kaltenboeck B. Rapid high-yield mRNA extraction for reverse-transcription PCR. Journal of biochemical and biophysical methods. 2007;70(3):507-9. doi: 10.1016/j.jbbm.2006.10.003. PubMed PMID: 17125842; PubMed Central PMCID: PMC1864960.

A466. Wang RH, Li C, Deng CX. Liver steatosis and increased ChREBP expression in mice carrying a liver specific SIRT1 null mutation under a normal feeding condition. International journal of biological sciences. 2010;6(7):682-90. PubMed PMID: 21103071; PubMed Central PMCID: PMC2990071.

A467. Wang X, Zheng Y, Shen F, Rao C. Theoretical analysis of tuning coherent laser array for several applications. Journal of the Optical Society of America A, Optics, image science, and vision. 2012;29(5):702-10. doi: 10.1364/JOSAA.29.000702. PubMed PMID: 22561928.

A468. Wang Y, Arsenault G, Riddell N, McCrindle R, McAlees A, Martin JW. Perfluorooctane sulfonate (PFOS) precursors can be metabolized enantioselectively: principle for a new PFOS source tracking tool. Environmental science & technology. 2009;43(21):8283-9. doi: 10.1021/es902041s. PubMed PMID: 19924957.

A469. Webb JG, Maisano F, Vahanian A, Munt B, Naqvi TZ, Bonan R, et al. Percutaneous suture edge-to-edge repair of the mitral valve. EuroIntervention : journal of EuroPCR in collaboration with the Working Group on Interventional Cardiology of the European Society of Cardiology. 2009;5(1):86-9. PubMed PMID: 19577987.

A470. Westlake BC, Brennaman MK, Concepcion JJ, Paul JJ, Bettis SE, Hampton SD, et al. Concerted electron-proton transfer in the optical excitation of hydrogen-bonded dyes. Proceedings of the National Academy of Sciences of the United States of America. 2011;108(21):8554-8. doi: 10.1073/pnas.1104811108. PubMed PMID: 21555541; PubMed Central PMCID: PMC3102414.

A471. White JR, Palejwala A. Bilateral shoulder fracture, dislocation and replacement: a first presentation of epilepsy. BMJ case reports. 2011;2011. doi: 10.1136/bcr.09.2011.4830. PubMed PMID: 22675040; PubMed Central PMCID: PMC3207796.

A472. Whiteaker P, Jimenez M, McIntosh JM, Collins AC, Marks MJ. Identification of a novel nicotinic binding site in mouse brain using [(125)I]-epibatidine. British journal of pharmacology. 2000;131(4):729-39. doi:

10.1038/sj.bjp.0703616. PubMed PMID: 11030722; PubMed Central PMCID: PMC1572375.

A473. Wierman JC. Percolation threshold is not a decreasing function of the average coordination number. Physical review E, Statistical, nonlinear, and soft matter physics. 2002;66(4 Pt 2):046125. PubMed PMID: 12443277.

A474. Williams PA, Cosme J, Ward A, Angove HC, Matak Vinkovic D, Jhoti H. Crystal structure of human cytochrome P450 2C9 with bound warfarin. Nature. 2003;424(6947):464-8. doi: 10.1038/nature01862. PubMed PMID: 12861225.

A475. Wilschanski M. Patterns of gastrointestinal disease associated with mutations of CFTR. Current gastroenterology reports. 2008;10(3):316-23. PubMed PMID: 18625144.

A476. Winance M. Pain, disability and rehabilitation practices. A phenomenological perspective. Disability and rehabilitation. 2006;28(18):1109-18. doi: 10.1080/09638280500531800. PubMed PMID: 16966231.

A477. Wolffenbuttel BH, Klaff LJ, Bhushan R, Fahrbach JL, Jiang H, Martin S. Initiating insulin therapy in elderly patients with Type 2 diabetes: efficacy and safety of lispro mix 25 vs. basal insulin combined with oral glucose-lowering agents. Diabetic medicine : a journal of the British Diabetic Association. 2009;26(11):1147-55. doi: 10.1111/j.1464-5491.2009.02824.x. PubMed PMID: 19929994.

A478. Wu B, Wang X, Zhang JH. Cardiac damage after subarachnoid hemorrhage. Acta neurochirurgica Supplement. 2011;110(Pt 1):215-8. doi: 10.1007/978-3-7091-0353-1_37. PubMed PMID: 21116942.

A479. Wu GS, Korsgren O, Zhang JG, Song ZS, Van Rooijen N, Tibell A. Role of macrophages and natural killer cells in the rejection of pig islet xenografts in mice. Transplantation proceedings. 2000;32(5):1069. PubMed PMID: 10936361.

A480. Yanagisawa R, Takano H, Inoue K, Ichinose T, Yoshida S, Sadakane K, et al. Complementary DNA microarray analysis in acute lung injury induced by lipopolysaccharide and diesel exhaust particles. Experimental biology and medicine. 2004;229(10):1081-7. PubMed PMID: 15522845.

A481. Yap EP, Koh VW, Loh JJ, Ng YY, Ooi EE. South-Asian tsunami. Lancet. 2005;365(9463):933. doi: 10.1016/S0140-6736(05)71069-2. PubMed PMID: 15766984.

A482. Yaron A, Huang PH, Cheng HJ, Tessier-Lavigne M. Differential requirement for Plexin-A3 and -A4 in mediating responses of sensory and sympathetic neurons to distinct class 3 Semaphorins. Neuron. 2005;45(4):513-23. doi: 10.1016/j.neuron.2005.01.013. PubMed PMID: 15721238.

A483. Yokoyama Y, Ebata T, Igami T, Sugawara G, Nagino M. Is the enteral replacement of externally drained pancreatic juice valuable after pancreatoduodenectomy? Surgery today. 2014;44(2):252-9. doi: 10.1007/s00595-013-0522-8. PubMed PMID: 23494105.

A484. Yoon PW, Scheuner MT, Jorgensen C, Khoury MJ. Developing Family Healthware, a family history screening tool to prevent common chronic diseases. Preventing chronic disease. 2009;6(1):A33. PubMed PMID: 19080039; PubMed Central PMCID: PMC2644613.

A485. Yoshida T, Goto S, Kawakatsu M, Urata Y, Li TS. Mitochondrial dysfunction, a probable cause of persistent oxidative stress after exposure to ionizing radiation. Free radical research. 2012;46(2):147-53. doi: 10.3109/10715762.2011.645207. PubMed PMID: 22126415.

A486. Yu SH, Su HM, Lin TH, Lee KT, Voon WC, Lai WT, et al. Acute cardiogenic pulmonary edema induced by severe hypoglycemia--a rare case report. International journal of cardiology. 2013;168(3):e94-5. doi: 10.1016/j.ijcard.2013.07.122. PubMed PMID: 23920060.

A487. Yu Y, Ramsay JA, Ramsay BA. On-line estimation of dissolved methane concentration during methanotrophic fermentations. Biotechnology and bioengineering. 2006;95(5):788-93. doi: 10.1002/bit.21050. PubMed PMID: 16850500.

A488. Yuan S, Yu X, Asara JM, Heuser JE, Ludtke SJ, Akey CW. The holo-apoptosome: activation of procaspase-9 and interactions with caspase-3. Structure. 2011;19(8):1084-96. doi: 10.1016/j.str.2011.07.001. PubMed PMID: 21827945; PubMed Central PMCID: PMC3155825.

A489. Yuen OY, Choy PY, Chow WK, Wong WT, Kwong FY. Synthesis of 3-cyanoindole derivatives mediated by copper(I) iodide using benzyl

cyanide. The Journal of organic chemistry. 2013;78(7):3374-8. doi: 10.1021/jo3028278. PubMed PMID: 23448701.

A490. Zakzanis KK, Leach L. Evidence for a shrinking span of personal and present existence in dementia of the Alzheimer's type. Brain and cognition. 2002;49(2):249-53. PubMed PMID: 15259403.

A491. Zaldivar JM, Baraibar J. A biology-based dynamic approach for the reconciliation of acute and chronic toxicity tests: application to Daphnia magna. Chemosphere. 2011;82(11):1547-55. doi: 10.1016/j.chemosphere.2010.11.062. PubMed PMID: 21168184.

A492. Zenge MO, Vogt FM, Brauck K, Jokel M, Barkhausen J, Kannengiesser S, et al. High-resolution continuously acquired peripheral MR angiography featuring partial parallel imaging GRAPPA. Magnetic resonance in medicine : official journal of the Society of Magnetic Resonance in Medicine / Society of Magnetic Resonance in Medicine. 2006;56(4):859-65. doi: 10.1002/mrm.21033. PubMed PMID: 16964615.

A493. Zhang M, Liu N, Tang W. Stereoselective total synthesis of hainanolidol and harringtonolide via oxidopyrylium-based [5 + 2] cycloaddition. Journal of the American Chemical Society. 2013;135(33):12434-8. doi: 10.1021/ja406255j. PubMed PMID: 23930656; PubMed Central PMCID: PMC3798225.

A494. Zhao Y, Tan YS, Aupperlee MD, Langohr IM, Kirk EL, Troester MA, et al. Pubertal high fat diet: effects on mammary cancer development. Breast

cancer research : BCR. 2013;15(5):R100. doi: 10.1186/bcr3561. PubMed PMID: 24156623; PubMed Central PMCID: PMC3978633.

A495. Zhou J, Mauerer K, Farina L, Gribben JG. The role of the tumor microenvironment in hematological malignancies and implication for therapy. Frontiers in bioscience : a journal and virtual library. 2005;10:1581-96. PubMed PMID: 15769648.

496. Zhu F, Liu Q, Fu Y, Shen B. Segmentation of neuronal structures using SARSA (lambda)-based boundary amendment with reinforced gradient-descent curve shape fitting. PloS one. 2014;9(3):e90873. doi: 10.1371/journal.pone.0090873. PubMed PMID: 24625699; PubMed Central PMCID: PMC3953327.

A497. Zhu L, Tee KL, Roccatano D, Sonmez B, Ni Y, Sun ZH, et al. Directed evolution of an antitumor drug (arginine deiminase PpADI) for increased activity at physiological pH. Chembiochem : a European journal of chemical biology. 2010;11(5):691-7. doi: 10.1002/cbic.200900717. PubMed PMID: 20157910.

A498. Zhuang H, Cunnane ME, Ghesani NV, Mozley PD, Alavi A. Chest tube insertion as a potential source of false-positive FDG-positron emission tomographic results. Clinical nuclear medicine. 2002;27(4):285-6. PubMed PMID: 11914670.

A499. Ziogas DE, Katsios C, Roukos DH. From traditional molecular biology to network oncology. Future oncology. 2011;7(2):155-9. doi: 10.2217/fon.10.190. PubMed PMID: 21345133.

A500. Zuberi SM, Hanna MG. Ion channels and neurology. Archives of disease in childhood. 2001;84(3):277-80. PubMed PMID: 11207185; PubMed Central PMCID: PMC1718682.
