# Supplementary material for: Reproducible Research Practices and Transparency across the Biomedical Literature
Source: PLoS Biol. 2016 Jan 4;14(1):e1002333. doi: 10.1371/journal.pbio.1002333 (PMC4699702; doi:10.1371/journal.pbio.1002333)
Supplement: S1 Protocol — (PDF) [file pbio.1002333.s004.pdf]

The inability to replicate published research has been an ongoing concern in the scientific community [1]. There is disconcerting evidence from basic molecular and animal modeling research that a portion of published articles lack reproducibility [2], which could potentially be related to the increase in lack of efficacy of clinical trials [3, 4]. It has been suggested that the lack of transparency of the data is linked to the inability to replicate findings [5]. Although previous publications have reported on the lack of reproducibility and transparency in published data, a detailed identification of their predictive indicators has not been developed.

Aims: The overall goal is to evaluate the trend in reproducibility and transparency in a random sample of published biomedical journal articles. Additionally, the project aims to identify predictors for reproducibility and transparency through study characteristics. The plan is to derive empirical data on indicators of transparency and reproducibility that have been proposed in the Lancet series on increasing value and reducing waste in research by Ioannidis et al.[1]

Objective1: Measure a sample of 500 biomedical journal articles, chosen randomly based on Pubmed Identification (PMID) numbers spanning from PMID # 10,000,000 to PMID # 25,000,000. The random sample will include English language articles published between 2000 and 2014.

Methodology overview: PMID numbers, ranging from 10,000,000 to 25,000,000 were inputted into OpenEpi (version 3.02) random number generator to select a random sample of 750 PMID numbers (S1 Table). Beginning from the first number generated (number 1 in column 1, row 1, S1 Table), numbers were verified for eligibility in sequence until 500 eligible PMID numbers were chosen (S2 Table). Of the original 750 numbers, 742 were checked, with 242 being ineligible (54 unfound, 100 before year 2000, 35 not in English, and 53 not in English and before year 2000). The selected article distribution of PMID numbers (by year) was compared to the overall distribution of PMID numbers by year for English articles. The sample was found to be representative of the overall distribution, χ^2 (df=14), p>0.05. The sample was independently characterized and cross-compared by two investigators (SAI and JDW) into 7 study characteristic categories (S3 Table): 1. no research (items with no data such as editorials, commentaries, news, comments and non-systematic expert reviews, 2. models/modeling or software or script or methods without empirical data (other than simulations), 3. case report or series (humans only, with or without review of the literature) 4. randomized clinical trials (humans only) 5. systematic reviews and/or meta-analyses (humans only) 6. cost effectiveness or decision analysis (humans only), and 7. other (empirical data that includes uncontrolled study (human), controlled non-randomized study (human) or basic science studies). A third reviewer (JPAI) reassessed articles with arbitration discrepancies.

The sample was found to be primarily composed of articles with empirical data (70%), with the majority of those articles consisting of uncontrolled or controlled non-randomized human studies or basic science research.

InCites Essential Science Indicators was used to determine the field of study. Briefly, the journal for each index paper was first selected in InCites Essential Science Indicators. Then utilizing the documents tab, the Highly Cited Papers for each journal were examined. Data extracted were as follows, for articles with one field listed under the Research Fields for each of the Highly Cited Papers, the type of field was recorded. If an article had more than one research field, we would look at the first five cited journals cited by the index article. The journal names for these articles were then selected in InCities Essential Science Indicators. If the majority of the journals listed the same field of study, this field of study was used for the index paper. If there was no majority field of study, a field of study was selected based on the best judgment of the reviewers (JPAI, SAI & JDW). If the journal was not found on InCities Essential Science Indicators or the journal had no results when selecting the documents tab, the journal was then selected in InCities Journal Citation Reports. The first category listed on the Journal Profile page was selected in order to find the highest cited journal in that category. The highest cited journal was then selected in InCities Essential Science Indicators to determine the field listed under the Research Fields for each of the Highly Cited Papers.

If the journal could not be located on InCities Journal Citation Reports, a field of study was selected based on the best judgment of the reviewers (JPAI, SAI & JDW). Publications in research fields not directly related to biomedical research (Chemistry, Physics, Computer Science, Economics & Business, Engineering, Geosciences, Material Science, Mathematics, Physics, and Space Science) were further excluded from analysis. For this sample, a total of 59 articles were excluded due to field of study (S4 Table).

InCites Journal Citation Reports was used to determine 2013 journal impact factor. No information was recorded for journals without an impact factor for 2013.

Availability of free access in PubMed Central was based on assignment of a PCMID (yes/no). Study and individual researcher funding will also be assessed (0=no mention, 1=no funding, 2=public, 3=private industry, 4=other, 5=combination of 2&3; 6=combination of 2&4; 7=combination of 3&4, 8=combination of 2-4). All of the studies with public funding were then examined to determine whether they had NIH (or any of the 27 separate NIH institutes or centers) funding (1=yes, 0=no), NSF funding (1=yes, 0=no), or Other public funding (1=yes, 0=no) Individual investigator funding will be excluded from this assessment if listed under possible conflicts of interest. Field of study will also be determined for each article utilizing InCites Essential Science Indicators as described in Objective 1 methodology.

Based on our initial article characteristic classification, publications with data and analyses (classification categories 4-7, S3 Table), will be assessed for publically available full protocols and datasets, conflict of interests, and patterns of reproducibility. For the items that do not include data and analyses, categories 1-3, only statements of conflict will be investigated, since protocols, datasets, and reproducibility are not relevant.

1. To assess the proportion of publications that have publically available protocols, we will review the methods sections for direct protocol listing or reference to the source for available protocol. For the studies that have publically available protocols, we shall also report whether or not the available protocols cover all or part of the presented analyses. Data extracted: 0=no protocols, 1=partial coverage, 2=full coverage
2. To identify the proportion of publications that have publically available datasets, chosen manuscripts will be examined for access to the datasets that stand behind the analyses presented in the paper. If so, we shall also record whether the available datasets cover all or part of the presented analyses. Data extracted: 0=no datasets, 1=partial coverage, 2=full coverage
3. To identify reported conflict of interests, the proportion of publications that state that none of the authors have any conflicts of interest, as attested by declaration statements and checked by reviewers, will be identified. We will capture specifically whether each article includes a

statement on conflict of interest disclosures or not; and, if yes, whether any conflicts of interest are disclosed. Data extracted: 0=no statement, 1=statement exists, conflicts present, 2=statement exists, no conflicts

4. To determine reproducibility patterns, the proportion of publications whose findings have been replicated will be measured. Web of Knowledge (v 5.14) will be utilized to identify the number of citations to each of the index papers of interest as of mid-2014. Furthermore, the citing papers of each index paper will be examined to identify systematic reviews and/or meta-analyses and/or studies that claim to try to replicate findings from the index paper. The citing papers will be screened at the title level, and those that seem potentially relevant will also be screened at the abstract, introduction, and possibly full-text level. Eligible citing papers that are systematic reviews and/or meta-analyses and/or replications will be downloaded in full text starting with the one that is published earlier.

    1. To measure research originality, abstracts from papers that include data and analyses (classification categories 4-7, S3 Table) will be examined for clear statements for study novelty or replication.

        Data extracted D1: 0=based on the abstract and/or introduction, the index paper claims that it presents some novel findings, 1=based on its abstract, the index paper clearly claims that it is a replication effort

trying to validate previous knowledge or based on the abstract and introduction it is inferred that the index paper is a replication trying to validate previous knowledge, 2=based on the abstract and/or introduction, it claims to be both novel and replicate previous findings, 3=no statement or unclear statement in the abstract and/or introduction about whether the index paper presents a novel finding or replication OR no distinct abstract and introduction.

2. Randomized clinical trials and other empirical data publications (classification categories 4 and 7, S3 Table) will further be assessed for articles citing the sample publication in an English language systematic reviews and/or meta-analysis (variable D2) and for articles replicating the sample publication (variable D3).

Data extracted D2: 0=no systematic review and/or meta-analysis has ever cited the index paper, 1=at least one systematic review and/or meta-analysis has cited the index paper but none has included any of its data in quantitative syntheses for any outcome, 1.5 = at least one systematic review and/or meta-analysis has cited the index paper but has provided reasons for not including any of its data for quantitative

syntheses for any outcome, 2=at least one systematic review and/or meta-analysis has cited the index paper and has included some of its data in quantitative synthesis for at least one outcome.

Data extracted D3: 0=no citing article identified claiming to be a replication attempt of the index paper, 1=at least one citing article identified claiming to be a replication attempt of the index paper.

We will not focus on the detailed results of the systematic reviews, meta-analyses, and replication studies, since our sample is expected to be underpowered and inefficient to detect whether specific results are indeed replicated or not. We focus simply on whether replication and integration in systematic reviews/meta-analyses of multiple studies has been considered and performed or not. Moreover, we anticipate that the majority of index papers will not have truly new discoveries, but may be operating in a knowledge space where other past studies may also have operated. Studies will be considered novel if the abstract and/or introduction a) claim to investigate new hypotheses, b) claim to develop and test new methods, c) claim to be the

first to investigate something that has not been examined before, or d) include any statement about new insights. For index papers, we do not aim to decipher which of these index studies are indeed proposing entirely new discoveries, or making claims for some novel findings without these actually being novel.

Reference

1. Ioannidis JP. Why most published research findings are false. PLoS medicine. 2005;2(8):e124. doi: 10.1371/journal.pmed.0020124. PubMed PMID: 16060722; PubMed Central PMCID: PMC1182327.
2. Prinz F, Schlange T, Asadullah K. Believe it or not: how much can we rely on published data on potential drug targets? Nature reviews Drug discovery. 2011;10(9):712. doi: 10.1038/nrd3439-c1. PubMed PMID: 21892149.
3. Arrowsmith J. Trial watch: Phase II failures: 2008-2010. Nature reviews Drug discovery. 2011;10(5):328-9. doi: 10.1038/nrd3439. PubMed PMID: 21532551.
4. Arrowsmith J. Trial watch: phase III and submission failures: 2007-2010. Nature reviews Drug discovery. 2011;10(2):87. doi: 10.1038/nrd3375. PubMed PMID: 21283095.
5. Landis SC, Amara SG, Asadullah K, Austin CP, Blumenstein R, Bradley EW, et al. A call for transparent reporting to optimize the predictive value of preclinical research. Nature. 2012;490(7419):187-91. doi:

10.1038/nature11556. PubMed PMID: 23060188; PubMed Central PMCID: PMC3511845.
