# Supplementary material for: Reproducible Research Practices and Transparency across the Biomedical Literature
Source: PLoS Biol. 2016 Jan 4;14(1):e1002333. doi: 10.1371/journal.pbio.1002333 (PMC4699702; doi:10.1371/journal.pbio.1002333)
Supplement: S1 SAS Code — (PDF) [file pbio.1002333.s005.pdf]

```
****************************************
*                                      *
* This program was designed to assess   *
* the data collected from a random      *
* sample of 500 journals                *
* Date: 10.08.15                       *
****************************************;

/* 1: Create data trans in work library based on imported excel
document */

DATA trans;
      SET TRANSR.Trans;

*** create new variables for use;
      IF cit_art=0 THEN cit_art1=0;
      ELSE IF cit_art=1 THEN cit_art1=1;

*** define studyfield for CLINICAL MEDICINE as 1 and all non-
clinical medicine as 0;
      If studyfield='CLINICAL MEDICINE' then studyfield1=1;

      else IF studyfield='NODOC' then studyfield1=.; /* Just incase
any NODOC or NESI remain */
      else If studyfield='NOESI' then studyfield1=.;
      else studyfield1=0;

*** create new funding variables - with funding1=5 for some
combination of 2-4;

      *** 0=no mention, 1=no funding, 2=Public,
3=private, 4= other, 5= combination of funding 2&3,
6=combination 2&4, 7= combination 3&4,
8=combination 2-4, 88=not applicable;

      if funding=0 then funding1=0;
      else if funding =1 then funding1=1;
      else if funding=2 then funding1=2;
      else if funding=3 then funding1=3;
      else if funding=4 then funding1=4;
      else if funding=5 then funding1=5;
      else if funding=6 then funding1=5;
      else if funding=7 then funding1=5;
      else if funding=8 then funding1=5;
      else if funding=88 then funding1=88;


*** Citing article being replicated make sure those with no data are
not cited;
      if cit_art=0 then cit_art1=0;
      else if cit_art=1 then cit_art1=1;
      else if cit_art=99 then cit_art1=0; *if no data available make
no cite;
      else if cit_art=88 then cit_art1=88;
```

```
*** Citation by a systematic review;

***0=no systematic review and/or meta-analysis has ever cited the
index paper,
1=at least one systematic review and/or meta-analysis has cited the
index paper
but none has included any of its data in quantitative syntheses for
any outcome,
2=at least one systematic review and/or meta-analysis has cited
the index paper and has included some of its data in quantitative
synthesis for at least one outcome 1.5=excluded data from review,
4= combination of 1 and 1.5, 5=combination of 1 and 2,
6=combination of 1.5 and 2, 7= combination of 1, 1.5 and 2,
88=Not Applicable, 99=No Data Available ;

***For Cit_Sysrev =5 or 6 or 7, making it Cit_Sysrev 3: Any
combination of;

      if Cit_Sysrev=0 then Cit_Sysrev1=0;
      else if Cit_Sysrev=1 then Cit_Sysrev1=1;
      else if Cit_Sysrev=1 then Cit_Sysrev1=2;
      else if Cit_Sysrev=1.5 then Cit_Sysrev1=1.5;
      else if Cit_Sysrev=4 then Cit_Sysrev1=4;
      else if Cit_Sysrev=5 then Cit_Sysrev1=4;
      else if Cit_Sysrev=6 then Cit_Sysrev1=4;
      else if Cit_Sysrev=7 then Cit_Sysrev1=4;
      else if Cit_Sysrev=88 then Cit_Sysrev1=88;
      else if Cit_Sysrev=99 then Cit_Sysrev1=99;

*** Extra variable, incase needed in later analysis;

      if Cit_Sysrev=0 then Cit_Sysrev2=0;
      else if Cit_Sysrev=1 then Cit_Sysrev2=1;
      else if cit_sysrev=2 then Cit_Sysrev2=2;
      else if cit_sysrev=1.5 then Cit_Sysrev2=1.5;
      else if Cit_Sysrev=88 then Cit_Sysrev2=88;
      else if Cit_Sysrev=99 then Cit_Sysrev2=0;*if no data avialable
make no cite;


*** Replication of study
/* 0=based on the abstract and/or introduction,
      the index paper claims that it presents some novel findings,
      1=based on its abstract/intro, the index paper clearly claims
that
      it is a replication effort trying to validate previous
knowledge,
      4=unclear statement in the abstract, but based on its
introduction,
      it is inferred that the index paper is a replication trying to
      validate previous knowledge, 2=it claims to be both novel and
      replicate previous findings, 3=no statement in the abstract
and/or
```

```
      introduction about whether the index paper presents a novel
finding
      or replication,  5=no abstract/no introduction, 88=not
applicable
      if replication = 0 then replication1=0;

      else if replication = 1 then replication1 = 1;
      else if replication = 2 then replication1 = 2;
      else if replication = 3 then replication1 = 3;
      else if replication = 4 then replication1 = 1; *if unclear
abstract, but replication inferred by intro, make replication;
      else if replication = 5 then replication1 = 3; *if there is no
intro abstract then make unclear;
      else if replication = 88 then replication1 = 88;
      else if replication = 99 then replication1 = 99;

*** Create new categorical variable for impact factor;

      if 2 ge impact ge 0 then impact2a="0-2";
      else if 4 ge impact gt 2 then impact2a=">2-4";
      else if 6 ge impact gt 4 then impact2a=">4-6";
      else if impact gt 6 then impact2a=">6";
      else impact2a=" ";



*Create formats;

PROC FORMAT;

*Objective 1;


      VALUE fundingf         0="No Mention"
                                   1="No Funding"
                                   2="Government"
                                   3="Private"
                                   4="Other"
                                   5="Both Public and Private"
                                   6="Both Public and Other"
                                   7="Both Private and Other"
                                   8="Public, Private and Other"
                                   88="Not Applicable"
                                   ;
      VALUE funding1f  0="No Mention"
                                   1="No Funding"
                                   2="Public"
                                   3="Private"
                                   4="Other"
                                   5="Combination"
                                   88="Not Applicable"
                                   ;

      VALUE NIHFundf         1="True"
```

```
                        0="False"
                        88="Not Applicable"
                        ;
      VALUE NSFFundf         1="True"
                        0="False"
                        88="Not Applicable"
                        ;
      VALUE OtherFundf 1="True"
                        0="False"
                        88="Not Applicable"
                        ;
      VALUE Cit_artf         0="No Citing Article"
                        1="At Least 1 Citing Article"
                        88="Not Applicable"
                        99="No Data Available"
                        ;

      VALUE Cit_art1f        0="No Citing Article"
                        1="At Least 1 Citing Article"
                        88="Not Applicable"
                        ;

      VALUE Cit_Sysrevf0="No Citing Article"
                        1="At Least 1 Citing Article, No
Data Included"
                        2="At Least 1 Citing Article, Data
Included"
                        1.5="At Least 1 Citing Article,
Data Excluded"
                        88="Not Applicable"
                        99="No Data Avaliable"
                        ;

      VALUE Cit_Sysrev2f     0="No Citing Article"
                        1="At Least 1 Citing Article, No
Data Included"
                        2="At Least 1 Citing Article, Data
Included"
                        1.5="At Least 1 Citing Article,
Data Excluded"
                        88="Not Applicable"
                        ;

      VALUE Caserepf         1="True"
                        0="False"
                        ;
      VALUE Cedaf            1="True"
                        0="False"
                        ;
      VALUE Conflictsf 0="No Statement"
                        1="Statement Exists, Conflicts
Present"
                        2="Statement Exists, No Conflicts"
                        88="Not Applicable"
```

```
                                  ;
      VALUE Datasetf          0="No Dataset"
                                  1="Partial Coverage"
                                  2="Full Coverage"
                                  3="Cannot Be Determined"
                                  88="Not Appicable"
                                  ;
      VALUE Modelf            1="True"
                                  0="False"
                                  ;
      VALUE Noresf            1="True"
                                  0="False"
                                  ;
      VALUE Otherf            1="True"
                                  0="False"
                                  ;
      VALUE RCTf              1="True"
                                  0="False"
                                  ;
      VALUE Sysmetf           1="True"
                                  0="False"
                                  ;
      VALUE protocolf         0="No Protocol"
                                  1="Partial Coverage"
                                  2="Full Coverage"
                                  3="Cannot Be Determined"
                                  88="Not Appicable"
                                  ;

      VALUE Replicationf      0="Novel"
                                  1="Replication"
                                  2="Novel and Replication"
                                  3="No Statement on Novelty"
                                  5="No Abstract and Introduction"
                                  88="Not Applicable"
                                  99="No Data Available"
                                  ;

      VALUE Replication1f     0="Novel"
                                  1="Replication"
                                  2="Novel and Replication"
                                  3="No Statement on Novelty"
                                  88="Not Applicable"
                                  99="No Data Available"
                                  ;


*Objective 2;
      VALUE Animalf           1="True"
                                  0="False"
                                  77="No Abstract/Intro"
                                  88="Not Applicable"
                                  ;
      VALUE Clinic_Trif   1="True"
```

```
                                        0="False"
                                        88="Not Applicable"
                                        ;
        VALUE Genesf            1="True"
                                        0="False"
                                        77="No Abstract/Intro"
                                        88="Not Applicable"
                                        ;
        VALUE Humanf            1="True"
                                        0="False"
                                        77="No Abstract/Intro"
                                        88="Not Applicable"
                                        ;
        VALUE PCMIDf            1="True"
                                        0="False"
                                        88="Not Applicable"
                                        ;



RUN;

*Apply permanent Format;

DATA transf;
        SET trans;

                FORMAT Animal Animalf.;
                FORMAT Caserep Caserepf.;
                FORMAT Ceda Cedaf.;
                FORMAT Clinic_Tri Clinic_Trif.;
                FORMAT Conflicts Conflictsf.;
                FORMAT Dataset Datasetf.;
                FORMAT Funding Fundingf.;
                FORMAT Funding1 Funding1f.;
                FORMAT Genes Genesf.;
                FORMAT Human Humanf.;
                FORMAT NIHFund NIHFundf.;
                FORMAT NSFFund NSFFundf.;  /*And here*/
                FORMAT OtherFund OtherFundf.;
                FORMAT Model Modelf.;
                FORMAT Nores Noresf.;
                FORMAT Other Otherf.;
                FORMAT PCMID PCMIDf.;
                FORMAT Protocol Protocolf.;
                FORMAT Replication Replicationf.;
                FORMAT Replication1 Replication1f.;
                FORMAT RCT RCTf.;
                FORMAT Sysmet Sysmetf.;
                FORMAT Cit_art Cit_artf.;
                FORMAT Cit_art1 Cit_artf1.;
                FORMAT Cit_Sysrev Cit_Sysrevf.;
                FORMAT Cit_Sysrev2 Cit_Sysrevf2.;
```

```
RUN;




******************************
*  Descriptive Data Analaysis *
* Make sure to remove all     *
* non-medical and N/A articles*
*****************************;

*Number of Medical Journals v non-Medical;
PROC FREQ;
     TABLES Medfield;
RUN;

*Distribution of type of research;
PROC FREQ;
     TABLES Nores*Model*Caserep*RCT*sysmet*Ceda*Other/LIST;
          WHERE Medfield=1;
RUN;

*Publically Available Protocols--WANT TO KEEP SYSMET, AND CEDA
HERE!!;
PROC FREQ;
     TABLES protocol;
          WHERE Medfield=1 and protocol NE 88;
RUN;

*Publically Available Datasets--WANT TO KEEP SYSMET AND CEDA HERE!!;
PROC FREQ;
     TABLES dataset;
          WHERE Medfield=1 and dataset NE 88 and caserep ne 1;
RUN;

*Funding;
PROC FREQ;
     TABLES funding;
          WHERE Medfield =1 and funding NE 88;
RUN;

*NIH Funding;
PROC FREQ;
     TABLES NIHFund;
          WHERE Medfield=1 and NIHFund NE 88;
RUN;


*NSF Funding;
PROC FREQ;
     TABLES NSFFund;
          WHERE Medfield=1 and NSFFund NE 88;
RUN;
```

```
*Other Funding;
PROC FREQ;
      TABLES OtherFund;
            WHERE Medfield=1 and OtherFund NE 88;
RUN;

*breakdown of government funding into exclusive funding categories;
PROC FREQ;
      TABLES NIHFund*NSAfund*OtherFund/LIST;
            WHERE Medfield=1 and NIHFund NE 88 and NSAfund NE 88 and
otherfund NE 88;
RUN;


*Trends in patterns of funding by year;
*Trends for funding over time;
PROC FREQ;
      TABLES year*funding/LIST;
            WHERE medfield=1;
RUN;

*Trends for public funding over time;
PROC FREQ;
      TABLES year*NIHFund*NSAfund*OtherFund/LIST;
            WHERE Medfield=1 and NIHFund NE 88 and NSAfund NE 88 and
otherfund NE 88;
RUN;



*********************************************;
*                                           ;
*   Empi Data Only comparison                   ;
*                                         ;
*         REPLICATION                     ;
*                                         ;
*********************************************;

PROC FREQ;  *ensure new variable coded correctly;
      TABLES replication*replication1/LIST MISSING;
            WHERE Medfield =1 and replication NE 88 and sysmet ne 1
and ceda ne 1 and caserep ne 1;
RUN;

PROC FREQ;  *only want Empirical data without casrep, ceda and
sysmets (total of 259);
      TABLES replication1;
            WHERE Medfield =1 and replication NE 88 and sysmet ne 1
and ceda ne 1 and caserep ne 1;
RUN;
```

```
*******************************************;
*                                          ;
*   Empi Data Only comparison                    ;
*                                        ;
*         CITING ARTICLE                  ;
*                                        ;
*******************************************;


PROC FREQ;*only want Empirical data without casrep, ceda and sysmets
(total of 259);
      TABLES cit_art;
            WHERE Medfield =1 and replication ne 88 and sysmet ne 1
and ceda ne 1 and caserep ne 1;
RUN;


*check to ensure that cited articles are correct with dataset;

PROC PRINT;
      VAR PMID;
            WHERE cit_art=1;
RUN;



*******************************************;
*                                          ;
*   Empi Data Only comparison                    ;
*                                        ;
*         CITING SYSMET                  ;
*                                        ;
*******************************************;

*Citing systematic review or meta analysis;
PROC FREQ;*only want Empirical data without casrep, ceda and sysmets
(total of 259);
      TABLES cit_sysrev;
            WHERE Medfield =1 and replication NE 88  and sysmet ne 1
and ceda ne 1 and caserep ne 1;
RUN;

PROC print;*ensure have correct articles with data included (16=n;
      VAR pmid;
            WHERE Medfield =1 and (cit_sysrev=2 or cit_sysrev=5 or
cit_sysrev=6 or cit_sysrev=7);
RUN;

PROC PRINT;*ensure have correct articles with data excluded (3=n;
      VAR pmid;
            WHERE Medfield =1 and (cit_sysrev=1.5 or cit_sysrev=4 or
cit_sysrev=6 or cit_sysrev=7);
RUN;
```

```
PROC PRINT;*ensure have correct articles with no data included but 
cited (19=n;
      VAR pmid;
            WHERE Medfield =1 and (cit_sysrev=1 or cit_sysrev=4 or 
cit_sysrev=5 or cit_sysrev=7);
RUN;



*********************************************;
*                                            ;
*  ALL Data (n=441)                        ;
*                                          ;
*         CONFLICTS                        ;
*                                          ;
*********************************************;



*Conflicts of Interest all;

PROC FREQ;
      TABLES conflicts;
            WHERE Medfield =1 and conflicts NE 88;
RUN;

*Conflicts of Interest only RCTs (n=15);

PROC FREQ;
      TABLES conflicts;
            WHERE Medfield =1 and conflicts NE 88 and RCT=1;
RUN;

*Trends in Conflicts of Interest all (n=441);

*Conflicts by year;

PROC FREQ;
      TABLES year*conflicts/LIST;
            WHERE medfield=1;
RUN;


*********************************************;
*                                            ;
*  ALL Data (n=441)                        ;
*                                          ;
*         impact factor                          ;
*                                          ;
*********************************************;
*Journal Impact Factor for 2013;
PROC UNIVARIATE;
      VAR Impact2;
```

```
            WHERE Medfield=1;
RUN;

*Journal impact factor categorized;
PROC FREQ; *ensure variable created correctly;
      TABLES Impact2*impact2a/LIST MISSING;
            WHERE Medfield=1;
RUN;

PROC FREQ; *ensure variable created correctly;
      TABLES impact2a/LIST MISSING;
            WHERE Medfield=1;
RUN;

*********************************************;
*                                            ;
*  ALL Data (n=441)                        ;
*                                          ;
*         PMCID                            ;
*                                          ;
*********************************************;

PROC FREQ;
      TABLES PMCID/LIST MISSING;
            WHERE Medfield=1;
RUN;



*********************************************;
*                                            ;
*  ALL Data (n=441)                        ;
*                                          ;
*         studyfield                       ;
*                                          ;
*********************************************;



*Study Field distribution for all  data;
PROC FREQ;
      TABLES studyfield/LIST MISSING;
            WHERE Medfield=1;
RUN;





*********************************************;
*                                            ;
*   clinical medicine v other              ;
*                                          ;
*                                          ;
```

```
*                                           ;
********************************************;

PROC FREQ;
     TABLES studyfield*studyfield1/LIST MISSING;
          WHERE medfield=1 ;
RUN;

********************************************;
*                                             ;
*   clinical medicin v other                     ;
*                   n=441                   ;
*         FUNDING                           ;
*                                           ;
********************************************;

PROC FREQ;
     TABLES funding*funding1/LIST MISSING; *ensure new variable
that consolidates combo funding correct;
RUN;

PROC FREQ; *compare all types of research for funding, including
artiles without empirical data;
     TABLES studyfield1*funding1/LIST MISSING;
          WHERE medfield=1 and studyfield1 =1;
RUN;

PROC FREQ; *compare all types of research for funding, including
artiles without empirical data;
     TABLES studyfield1*funding1/LIST MISSING;
          WHERE medfield=1 and studyfield1 =0;
RUN;

PROC FREQ;   *********WARNING: Computing exact p-values for this
problem may require much time and memory. Press the
system interrupt key to terminate exact computations.;
     TABLES studyfield1*funding1/EXACT FISHER;
          WHERE medfield=1;
RUN;



***Run monte carlo approximation on fisher exact;
PROC FREQ;
     TABLES studyfield1*funding1 / CHISQ EXPECTED;
          EXACT FISHER / MC;
RUN;


********************************************;
*                                             ;
*   clinical medicine v other               ;
*                   n=441                   ;
*         FUNDING NIH                       ;
```

```
*                                          ;
********************************************;


PROC FREQ; *compare all types of research for funding, including
artiles without empirical data;
      TABLES studyfield1*NIHFUND/LIST MISSING;
            WHERE medfield=1 and studyfield1 =1;
RUN;

PROC FREQ; *compare all types of research for funding, including
artiles without empirical data;
      TABLES studyfield1*NIHFUND/LIST MISSING;
            WHERE medfield=1 and studyfield1 =0;
RUN;

PROC FREQ;
      TABLES studyfield1*NIHFUND/EXACT FISHER;
            WHERE medfield=1;
RUN;




********************************************;
*                                            ;
*   clinical medicine v other              ;
*                      n=259               ;
*         REPLICATION                      ;
*                                          ;
********************************************;
PROC FREQ;  *only want Empirical data without casrep, ceda and
sysmets (total of 259);
      TABLES studyfield1*replication1/LIST MISSING;
            WHERE medfield=1 and replication ne 88 and studyfield1=0
and sysmet ne 1 and ceda ne 1 and caserep ne 1;
RUN; *clinical medicine studyfield1=1;

PROC FREQ;  *only want Empirical data without casrep, ceda and
sysmets (total of 259);
      TABLES studyfield1*replication1/LIST MISSING;
            WHERE medfield=1 and replication ne 88 and studyfield1=1
and sysmet ne 1 and ceda ne 1 and caserep ne 1;
RUN; *clinical medicine studyfield1=1;

PROC FREQ;  *only want Empirical data without casrep, ceda and
sysmets (total of 259);
      TABLES studyfield1*replication1/EXACT FISHER;
            WHERE medfield=1 and replication ne 88  and sysmet ne 1
and ceda ne 1 and caserep ne 1;
RUN; *clinical medicine studyfield1=1;


********************************************;
```

```
*                                            ;
*   cli med v other comparison             ;
*                                   n=259    ;
*         CITING ARTICLE                     ;
*                                          ;
*********************************************;
PROC FREQ;
      TABLES cit_art*cit_art1/LIST MISSING; *make sure coded
correctly;
            WHERE medfield=1 and replication ne 88 and sysmet ne 1
and ceda ne 1;
RUN;

PROC FREQ;
      TABLES studyfield1*cit_art1/LIST MISSING;
            WHERE medfield=1 and studyfield1 =1 and replication ne 88
and sysmet ne 1 and ceda ne 1;
RUN;



PROC FREQ;
      TABLES studyfield1*cit_art1/LIST MISSING;
            WHERE medfield=1 and studyfield1 =0 and replication ne 88
and sysmet ne 1 and ceda ne 1;
RUN;


PROC FREQ;
      TABLES studyfield1*cit_art1/EXACT FISHER;
            WHERE medfield=1 and replication ne 88 and sysmet ne 1
and ceda ne 1;
RUN;




*********************************************;
*                                            ;
*   cli med v other comparison             ;
*                                   n=259    ;
*         CITING SYSMET                      ;
*                                          ;
*********************************************;
PROC FREQ;
            TABLES cit_sysrev*cit_sysrev2/LIST;
RUN;
PROC FREQ;
      TABLES studyfield1*Cit_Sysrev2/LIST MISSING;
            WHERE medfield=1 and studyfield1 =1 and replication ne 88
and sysmet ne 1 and ceda ne 1;
RUN;
```

```
PROC FREQ;
      TABLES studyfield1*Cit_Sysrev2/LIST MISSING;
            WHERE medfield=1 and studyfield1 =0 and replication ne 88
and sysmet ne 1 and ceda ne 1;
RUN;


PROC FREQ;
      TABLES studyfield1*cit_sysrev2/EXACT FISHER;
            WHERE medfield=1 and replication ne 88 and sysmet ne 1
and ceda ne 1;
RUN;




*********************************************;
*                                           ;
*  cli med v other comparison                     ;
*                                         ;
*         CONFLICTS     n=441             ;
*                                         ;
*********************************************;


PROC FREQ;
      TABLES studyfield1*conflicts/LIST MISSING;
            WHERE medfield=1;
RUN; *clinical medicine studyfield1=1;

PROC FREQ;
      TABLES studyfield1*conflicts/LIST MISSING;
            WHERE medfield=1 and studyfield1=1;
RUN; *clinical medicine studyfield1=1;
PROC FREQ;
      TABLES studyfield1*conflicts/LIST MISSING;
            WHERE medfield=1 and studyfield1=0;
RUN; *clinical medicine studyfield1=1;

PROC FREQ;
      TABLES studyfield1*conflicts/EXACT FISHER;
            WHERE medfield=1;
RUN; *clinical medicine studyfield1=1;


*********************************************;
*                                           ;
*   clinical medicine v other             ;
*                     n=441               ;
*         PMCID                           ;
*                                         ;
*********************************************;

PROC FREQ;
```

```
      TABLES studyfield1*PMCID/LIST MISSING;
            WHERE medfield=1 and studyfield1=1;
RUN; *clinical medicine studyfield1=1;

PROC FREQ;
      TABLES studyfield1*PMCID/LIST MISSING;
            WHERE medfield=1 and studyfield1=0;
RUN; *clinical medicine studyfield1=1;

PROC FREQ;
      TABLES studyfield1*PMCID/EXACT FISHER;
            WHERE medfield=1;
RUN; *clinical medicine studyfield1=1;


*********************************************;
*                                           ;
*   additional code requested by reviewers   ;
*           to replicate table 1 with      ;
*  empirical studies only (n=304)          ;
*                                          ;
*********************************************;


*********************************************;
*                                           ;
*   additional code requested by reviewers   ;
*           to replicate table 1 with      ;
*  empirical studies only (n=304)          ;
*                                          ;
*********************************************;

*Distribution of type of research for studies with empirical data
only n=304;
PROC FREQ;
      TABLES Nores*Model*Caserep*RCT*sysmet*Ceda*Other/LIST;
            WHERE Medfield=1 and nores ne 1 and model ne 1;
RUN;


*Distribution of PCMID for studies with empirical data
only n=304;

PROC FREQ;
      TABLES PMCID/LIST MISSING;
            WHERE Medfield=1 and nores ne 1 and model ne 1;
RUN;

*Distribution of impact factor for studies with empirical data
only n=304;

PROC FREQ;
      TABLES impact2a/LIST MISSING;
            WHERE Medfield=1 and nores ne 1 and model ne 1;
```

```
RUN;

PROC UNIVARIATE;
      VAR Impact2;
            WHERE Medfield=1 and nores ne 1 and model ne 1;

RUN;


*Distribution of field of research for studies with empirical data
only n=304;
PROC FREQ;
      TABLES studyfield/LIST MISSING;
      WHERE Medfield=1 and nores ne 1 and model ne 1;

RUN;


PROC FREQ;
      TABLES impact2a/LIST MISSING;
            WHERE Medfield=1 and nores ne 1 and model ne 1;

RUN;


/* PMCID among empirical data NON CLINICAL MEDICINE FIELD  */
PROC FREQ;
      TABLES studyfield1*PMCID/LIST MISSING;
            WHERE medfield=1 and studyfield1=0 and nores ne 1 and
model ne 1;
RUN; *clinical medicine studyfield1=1;

/*PMCID among empirical data CLINICAL MEDICINE FIELD */
PROC FREQ;
      TABLES studyfield1*PMCID*medfield/LIST MISSING;
            WHERE medfield=1 and studyfield1=1 and nores ne 1 and
model ne 1;
RUN; *clinical medicine studyfield1=1;




/* Funding for Clinical Medicine Field with Empirical Data */
PROC FREQ;
      TABLES studyfield1*funding1*medfield/LIST MISSING;
            WHERE medfield=1 and studyfield1=1 and nores ne 1 and
model ne 1;
RUN; *clinical medicine studyfield1=1;

PROC FREQ;/*Fumding for NON Clinical Medicine Field */
      TABLES studyfield1*funding1*medfield/LIST MISSING;
            WHERE medfield=1 and studyfield1=0 and nores ne 1 and
model ne 1;
```

```
RUN; *clinical medicine studyfield1=1;

PROC FREQ;/*Funding for empirical data studies among CLINICAL MED */
      TABLES studyfield1*funding1*medfield/LIST MISSING;
            WHERE medfield=1 and studyfield1=1 and nores ne 1 and
model ne 1;
RUN; *clinical medicine studyfield1=1;


PROC FREQ;/*Conflicts for empirical data studies among CLINICAL MED
*/
      TABLES studyfield1*conflicts*medfield/LIST MISSING;
            WHERE medfield=1 and studyfield1=1 and nores ne 1 and
model ne 1;
RUN; *clinical medicine studyfield1=1;


PROC FREQ;/*Conflicts for empirical data among NON CLINICAL MED */
      TABLES studyfield1*conflicts*medfield/LIST MISSING;
            WHERE medfield=1 and studyfield1=0 and nores ne 1 and
model ne 1;
RUN; *clinical medicine studyfield1=1;







*This is the code for methods section to compare the proportion of
this study articles by year to articles by year in pubmed that are
English only published between 2000 and 2014;

DATA a;
      INPUT year count expect;
            CARDS;

2000 19 22.02537386
2001 30 22.65847202
2002 36 23.41544225
2003 30 24.6793644
2004 31 26.67482332
2005 37 29.34960617
2006 34 31.47882279
2007 23 33.26761167
2008 32 35.53346351
2009 31 37.44389672
2010 27 40.43980017
2011 49 43.95662537
2012 55 46.89548919
2013 34 49.62475904
2014 32 32.55644951
;
```

```
PROC FREQ;
      TABLES year / NOCUM CHISQ TESTP=(4.4050748 4.5316944 4.6830884
4.9358729 5.3349647 5.8699212 6.2957646 6.6535223 7.1066927
7.4887793
8.08796 8.7913251 9.3790978 9.9249518 6.5112899);
      WEIGHT count;

RUN;
```
