# Supplementary material for: Reproducible Research Practices and Transparency across the Biomedical Literature
Source: PLoS Biol. 2016 Jan 4;14(1):e1002333. doi: 10.1371/journal.pbio.1002333 (PMC4699702; doi:10.1371/journal.pbio.1002333)
Supplement: S1 Table — (PDF) [file pbio.1002333.s006.pdf]

1. 17221499	151. 13391880	301. 10495830	451. 22655006	601. 21116942
2. 17467223	152. 15794343	302. 10899442	452. 11887089	602. 19114960
3. 22349201	153. 13623619	303. 21852490	453. 24328800	603. 16463060
4. 20566118	154. 24509220	304. 19051334	454. 13869388	604. 24307012
5. 22402381	155. 21777870	305. 24629269	455. 21151244	605. 24625699
6. 10755285	156. 24697300	306. 18452984	456. 15908535	606. 21145441
7. 19762479	157. 19364123	307. 23669047	457. 22173573	607. 18375142
8. 12140369	158. 23132104	308. 18917072	458. 22432794	608. 11716869
9. 17155884	159. 24188923	309. 17314962	459. 21827945	609. 13179795
10. 12905220	160. 24848787	310. 24582048	460. 12353298	610. 11315136
11. 11570872	161. 15420017	311. 11443035	461. 17170461	611. 24156623
12. 15852141	162. 12962197	312. 11258324	462. 21011460	612. 14479408
13. 10332964	163. 16791592	313. 15500907	463. 20688609	613. 16504851
14. 23048646	164. 20386259	314. 18756556	464. 17142822	614. 11324818
15. 16014249	165. 20196510	315. 22730448	465. 21950181	615. 15580405
16. 10788232	166. 11439346	316. 18538496	466. 11238717	616. 18739472
17. 14345855	167. 12797390	317. 20237113	467. 18154596	617. 16501348
18. 11035971	168. 16156622	318. 23588459	468. 23944022	618. 17257304
19. 20569066	169. 20396033	319. 13645566	469. 21725778	619. 21004937
20. 11207185	170. 10370148	320. 16850500	470. 14948903	620. 11914670
21. 15420359	171. 14374101	321. 24523190	471. 19446026	621. 22060480
22. 15836815	172. 15353349	322. 24045672	472. 22675040	622. 22970304
23. 22996984	173. 11784342	323. 12550227	473. 21575746	623. 16500254
24. 23834563	174. 13623500	324. 15699395	474. 18736495	624. 24387237
25. 11668675	175. 10673754	325. 11421246	475. 21555541	625. 16481151
26. 20079366	176. 18151469	326. 23169152	476. 20159571	626. 19439710
27. 15003652	177. 15613125	327. 18900919	477. 24880519	627. 22561928
28. 15274701	178. 19741212	328. 23884430	478. 24743622	628. 18303555
29. 17906692	179. 11519166	329. 14239947	479. 14732097	629. 24373224
30. 10747129	180. 16350274	330. 10566157	480. 10605859	630. 11918964
31. 13708517	181. 19577987	331. 10665622	481. 12479497	631. 11922580
32. 20950261	182. 20843821	332. 16413074	482. 14098542	632. 20729984
33. 12035268	183. 13632085	333. 21411802	483. 14767578	633. 18037441
34. 13388210	184. 24080467	334. 12619433	484. 19813929	634. 21804977
35. 10806516	185. 13294531	335. 21139778	485. 12734514	635. 13861569
36. 17591881	186. 14561913	336. 23193508	486. 16251691	636. 15714066
37. 15316506	187. 12972505	337. 12532543	487. 11893586	637. 20258683
38. 18625144	188. 23569200	338. 14931715	488. 18047023	638. 22931120

39. 23359937	189. 13273257	339. 21963068	489. 11731360	639. 23494105
40. 22778772	190. 21684540	340. 21260701	490. 10752330	640. 10273412
41. 11353767	191. 24230843	341. 11720610	491. 22642145	641. 14969306
42. 20633936	192. 19355891	342. 24422544	492. 20147811	642. 15247411
43. 19189154	193. 12841824	343. 20469608	493. 11239814	643. 13198290
44. 15378498	194. 23930656	344. 17302485	494. 17005538	644. 13003837
45. 13984122	195. 17949899	345. 12502615	495. 16114396	645. 22307414
46. 22744898	196. 13287034	346. 20446052	496. 19627661	646. 18862864
47. 15341661	197. 15590859	347. 24601975	497. 15776026	647. 20719603
48. 17541669	198. 15823379	348. 24609705	498. 15034836	648. 19710198
49. 14436100	199. 20157910	349. 21285628	499. 12991433	649. 11923688
50. 13389745	200. 21819248	350. 22545948	500. 21508095	650. 19008705
51. 22856313	201. 17513835	351. 11615282	501. 12300585	651. 15255597
52. 18304443	202. 19746724	352. 15911863	502. 15522845	652. 12445353
53. 19930443	203. 15762649	353. 15099778	503. 14282890	653. 20733120
54. 23920060	204. 12872323	354. 21889689	504. 15839897	654. 14536278
55. 13518638	205. 18563731	355. 12644405	505. 12127510	655. 10525554
56. 20239860	206. 24213899	356. 17073634	506. 24624290	656. 24264977
57. 17774525	207. 14869533	357. 13288776	507. 10012931	657. 22304654
58. 22525923	208. 17373341	358. 13274043	508. 12534561	658. 19702778
59. 21168184	209. 20143627	359. 11007203	509. 22590901	659. 14025115
60. 15346464	210. 17393762	360. 19665358	510. 23413181	660. 16748035
61. 12974476	211. 16376652	361. 12443277	511. 20182785	661. 19032132
62. 19885199	212. 12949370	362. 22036530	512. 16988585	662. 22488610
63. 10936361	213. 13698455	363. 17215498	513. 22572629	663. 12208796
64. 20390172	214. 15097753	364. 23369776	514. 21405595	664. 19019474
65. 18134351	215. 14580252	365. 18929787	515. 11492834	665. 15839136
66. 12415524	216. 11756495	366. 18784538	516. 21504058	666. 19500286
67. 15351035	217. 12038089	367. 14286955	517. 12652399	667. 16051745
68. 20424863	218. 23786186	368. 11053721	518. 24187687	668. 22445326
69. 23529422	219. 15259403	369. 22204470	519. 22176668	669. 10219235
70. 21992636	220. 17139556	370. 11262088	520. 21720135	670. 11959368
71. 10797007	221. 12074782	371. 11099892	521. 10242406	671. 14025309
72. 11116889	222. 15262923	372. 16552123	522. 11022739	672. 16089518
73. 22914732	223. 15820264	373. 12919547	523. 16699766	673. 10213525
74. 20450536	224. 20470882	374. 16445269	524. 15793201	674. 11961616
75. 22624652	225. 11030722	375. 14545945	525. 10619634	675. 14000601
76. 19080039	226. 21306373	376. 19789670	526. 12341484	676. 20134924

77. 19223875	227. 21740615	377. 24834474	527. 12843471	677. 12973082
78. 17590930	228. 16662793	378. 15841663	528. 18894041	678. 19342235
79. 15534605	229. 24936990	379. 16210130	529. 17004526	679. 15721238
80. 20086276	230. 22638695	380. 22155803	530. 11901867	680. 21334298
81. 13007787	231. 14042300	381. 13124627	531. 23880939	681. 13565813
82. 10041757	232. 12488208	382. 24508130	532. 22098088	682. 12747949
83. 19218230	233. 10016385	383. 10557870	533. 21338070	683. 21345132
84. 19882067	234. 17499754	384. 19607044	534. 23714371	684. 19929994
85. 11126131	235. 15089009	385. 20814100	535. 20292056	685. 21345133
86. 18701402	236. 11363099	386. 23393528	536. 14339400	686. 20910396
87. 19880938	237. 14967987	387. 11062883	537. 10418134	687. 20631536
88. 24485116	238. 23245650	388. 22251977	538. 21787166	688. 15872224
89. 20349343	239. 20573849	389. 11201420	539. 12524609	689. 12090368
90. 20901025	240. 18839516	390. 19434720	540. 18258861	690. 15769648
91. 16964615	241. 22078487	391. 20388435	541. 12119800	691. 13798751
92. 15711980	242. 23951529	392. 18100492	542. 15639101	692. 12576555
93. 14010476	243. 22463266	393. 23551804	543. 12314829	693. 13214108
94. 23798245	244. 17890680	394. 13797247	544. 21311092	694. 13227543
95. 20286732	245. 17394056	395. 23977311	545. 23637984	695. 12083064
96. 23615987	246. 20897189	396. 18521688	546. 16697924	696. 19317902
97. 21942227	247. 13279492	397. 12556780	547. 22334939	697. 21388740
98. 24947539	248. 13152648	398. 24924783	548. 21839589	698. 15893508
99. 22166884	249. 13452510	399. 20596287	549. 20258168	699. 12861225
100. 11947480	250. 18752476	400. 18484917	550. 12475314	700. 15897045
101. 10024412	251. 16391363	401. 21511401	551. 22560018	701. 12076871
102. 13800493	252. 12473404	402. 24306712	552. 14120361	702. 11611086
103. 24859500	253. 20688003	403. 11095370	553. 24243477	703. 15904649
104. 17518445	254. 19690666	404. 16709823	554. 17125842	704. 22127457
105. 12821058	255. 12633474	405. 16966231	555. 14744095	705. 14350792
106. 13716614	256. 21453382	406. 14608100	556. 13466919	706. 14039788
107. 13827809	257. 24223392	407. 24890534	557. 24778596	707. 21098937
108. 16006233	258. 22041029	408. 18425350	558. 11241058	708. 19177880
109. 24941466	259. 10137351	409. 13962290	559. 13499113	709. 21395605
110. 17107901	260. 17337370	410. 18413068	560. 24120809	710. 13459374
111. 15240255	261. 14278780	411. 15171622	561. 23518874	711. 12866940
112. 12325011	262. 16714398	412. 20317107	562. 15430181	712. 12015495
113. 15744710	263. 24447322	413. 22183776	563. 11654612	713. 10028717
114. 17994599	264. 16564837	414. 11706932	564. 17214376	714. 20951698

115. 21381345	265. 13597083	415. 24343247	565. 23510502	715. 21354130
116. 10298218	266. 12455123	416. 18115639	566. 12066058	716. 12010930
117. 12641861	267. 10387752	417. 24000752	567. 24875287	717. 19502191
118. 11801543	268. 18541463	418. 12660203	568. 15129540	718. 17908083
119. 16537947	269. 11732882	419. 11907511	569. 22601902	719. 22126415
120. 11591566	270. 23139447	420. 21013892	570. 19115167	720. 16025326
121. 23370674	271. 10062653	421. 14709978	571. 15133172	721. 24075804
122. 14943529	272. 21152650	422. 19613608	572. 10283992	722. 15551653
123. 17475505	273. 15656611	423. 14745864	573. 21629745	723. 17758986
124. 18050965	274. 11679892	424. 16838146	574. 10673897	724. 17585649
125. 24664042	275. 22224476	425. 22996987	575. 11730444	725. 20344636
126. 12984467	276. 23918719	426. 14069977	576. 22141682	726. 19174516
127. 20629267	277. 16216925	427. 21549194	577. 18401690	727. 20614107
128. 14291052	278. 15425272	428. 22736545	578. 19611987	728. 10273369
129. 11927861	279. 12319418	429. 20093909	579. 15496969	729. 20953679
130. 13541851	280. 19429392	430. 20094080	580. 13315932	730. 24554181
131. 12781759	281. 21103071	431. 21577214	581. 11307834	731. 20922103
132. 19848053	282. 11963753	432. 18285063	582. 16685302	732. 22130044
133. 10912709	283. 15755103	433. 12053929	583. 22045399	733. 12005548
134. 15240386	284. 13032668	434. 10603127	584. 20971890	734. 15623168
135. 11158473	285. 18440207	435. 16449812	585. 18114016	735. 11968902
136. 14662594	286. 11856509	436. 22850023	586. 23393680	736. 24312511
137. 19834284	287. 17336812	437. 13141094	587. 13822251	737. 24197879
138. 15816788	288. 22545142	438. 16864888	588. 10241404	738. 12550665
139. 22184384	289. 10987165	439. 15766984	589. 11309466	739. 24224134
140. 18187080	290. 21960570	440. 22718035	590. 19249517	740. 13932365
141. 14198753	291. 22838749	441. 21075107	591. 16517111	741. 10058109
142. 12972256	292. 21307395	442. 17236083	592. 23449402	742. 20419484
143. 13630294	293. 23763414	443. 15191577	593. 10491560	743. 22562973
144. 22756720	294. 17318980	444. 15310796	594. 11639812	744. 24286993
145. 19924957	295. 18790507	445. 12570214	595. 10125878	745. 16966440
146. 23267827	296. 21440001	446. 16460580	596. 18098489	746. 10755151
147. 11784578	297. 13235647	447. 22430022	597. 24260308	747. 17845991
148. 15042988	298. 23448701	448. 10291915	598. 14005621	748. 15968961
149. 22937664	299. 10072238	449. 24030913	599. 12266381	749. 23885219
150. 11345261	300. 19858589	450. 17002004	600. 16504852	750. 17697393
