# Supplementary material for: Reproducible Research Practices and Transparency across the Biomedical Literature
Source: PLoS Biol. 2016 Jan 4;14(1):e1002333. doi: 10.1371/journal.pbio.1002333 (PMC4699702; doi:10.1371/journal.pbio.1002333)
Supplement: S2 Table — (PDF) [file pbio.1002333.s007.pdf]

10665622	12949370	16850500	20086276	22545142
10673754	12962197	16864888	20093909	22545948
10747129	12972256	16964615	20094080	22560018
10755285	12972505	16966231	20147811	22561928
10797007	12973082	16988585	20157910	22572629
10899442	12974476	17002004	20159571	22590901
10912709	14561913	17004526	20182785	22624652
10936361	14580252	17005538	20237113	22638695
10987165	14608100	17073634	20349343	22642145
11007203	14662594	17107901	20386259	22655006
11030722	14709978	17125842	20390172	22675040
11035971	14744095	17139556	20424863	22718035
11053721	14745864	17142822	20469608	22730448
11062883	14767578	17155884	20470882	22736545
11095370	14967987	17170461	20566118	22744898
11099892	15003652	17214376	20569066	22778772
11126131	15034836	17215498	20573849	22856313
11158473	15089009	17302485	20596287	22914732
11201420	15099778	17314962	20614107	22931120
11207185	15129540	17318980	20631536	22970304
11238717	15133172	17336812	20633936	22996984
11239814	15171622	17337370	20688609	22996987
11241058	15191577	17373341	20729984	23132104
11262088	15240255	17393762	20733120	23139447
11307834	15240386	17394056	20814100	23169152
11309466	15247411	17467223	20843821	23193508
11315136	15255597	17475505	20922103	23245650
11324818	15259403	17499754	20950261	23267827
11345261	15262923	17513835	20951698	23359937
11353767	15274701	17518445	20953679	23369776
11421246	15310796	17541669	20971890	23370674
11439346	15316506	17591881	21075107	23393528
11443035	15341661	17890680	21098937	23393680
11570872	15351035	17906692	21103071	23448701
11591566	15353349	17949899	21116942	23449402
11668675	15378498	17994599	21139778	23494105
11706932	15496969	18037441	21145441	23510502
11716869	15500907	18047023	21152650	23518874
11730444	15522845	18154596	21168184	23529422

11731360	15534605	18187080	21306373	23551804
11732882	15551653	18258861	21311092	23588459
11756495	15580405	18303555	21334298	23615987
11784342	15590859	18375142	21338070	23637984
11784578	15613125	18401690	21345132	23669047
11801543	15623168	18413068	21345133	23714371
11887089	15639101	18425350	21354130	23763414
11893586	15656611	18440207	21388740	23786186
11907511	15699395	18452984	21395605	23798245
11914670	15711980	18484917	21405595	23834563
11918964	15714066	18521688	21411802	23880939
11922580	15721238	18538496	21440001	23884430
11923688	15744710	18541463	21453382	23918719
11927861	15755103	18563731	21504058	23920060
11959368	15762649	18625144	21508095	23930656
11961616	15766984	18701402	21511401	23951529
11968902	15769648	18752476	21549194	23977311
12005548	15793201	18756556	21555541	24000752
12010930	15794343	18784538	21575746	24045672
12015495	15816788	18790507	21577214	24075804
12035268	15820264	18929787	21629745	24080467
12038089	15823379	19008705	21684540	24120809
12053929	15836815	19019474	21725778	24156623
12066058	15839136	19032132	21740615	24187687
12074782	15839897	19051334	21777870	24188923
12076871	15841663	19080039	21787166	24197879
12083064	15852141	19114960	21804977	24223392
12090368	15872224	19115167	21819248	24260308
12119800	15893508	19174516	21827945	24264977
12127510	15897045	19177880	21839589	24306712
12140369	15904649	19218230	21852490	24312511
12208796	15908535	19249517	21889689	24328800
12325011	15911863	19317902	21960570	24343247
12353298	16006233	19342235	21963068	24373224
12415524	16025326	19355891	21992636	24387237
12443277	16051745	19364123	22036530	24422544
12445353	16089518	19429392	22041029	24447322
12473404	16156622	19434720	22045399	24485116
12475314	16210130	19439710	22060480	24508130
12479497	16216925	19446026	22078487	24509220
12488208	16350274	19500286	22098088	24523190

12502615	16376652	19502191	22125952	24582048
12524609	16391363	19577987	22126415	24601975
12534561	16413074	19627661	22127457	24609705
12556780	16445269	19665358	22130044	24624290
12570214	16449812	19690666	22155803	24625699
12576555	16460580	19710198	22173573	24629269
12619433	16463060	19741212	22176668	24664042
12633474	16481151	19746724	22183776	24697300
12641861	16500254	19762479	22184384	24743622
12644405	16501348	19789670	22224476	24778596
12660203	16504851	19834284	22304654	24834474
12734514	16504852	19848053	22334939	24848787
12747949	16517111	19858589	22349201	24859500
12781759	16537947	19880938	22402381	24875287
12797390	16685302	19882067	22430022	24880519
12821058	16697924	19885199	22432794	24890534
12841824	16699766	19924957	22445326	24924783
12861225	16709823	19929994	22463266	24936990
12866940	16714398	19930443	22488610	24941466
12872323	16791592	20079366	22525923	24947539
