# Supplementary material for: Reproducible Research Practices and Transparency across the Biomedical Literature
Source: PLoS Biol. 2016 Jan 4;14(1):e1002333. doi: 10.1371/journal.pbio.1002333 (PMC4699702; doi:10.1371/journal.pbio.1002333)
Supplement: S3 Table — (PDF) [file pbio.1002333.s008.pdf]

Number	Description
1	No research (items with no data such as editorials, commentaries, news, comments and non-systematic expert reviews)
2	Models/modeling or software or script or methods without empirical data (other than simulations)
3	Case report or series (humans only, with or without review of the literature)
4	Randomized clinical trials (human only)
5	Systematic reviews and/or meta-analysis (humans only)
6	Cost effectiveness or decision analysis (humans only)
7	Other (empirical data that includes uncontrolled study (human), controlled non-randomized study (human) or basic science studies)
