# Supplementary material for: Reproducible Research Practices and Transparency across the Biomedical Literature
Source: PLoS Biol. 2016 Jan 4;14(1):e1002333. doi: 10.1371/journal.pbio.1002333 (PMC4699702; doi:10.1371/journal.pbio.1002333)
Supplement: S4 Table — (PDF) [file pbio.1002333.s009.pdf]

11062883	17005538	20729984	22931120
11262088	17155884	20814100	23267827
11345261	17302485	21098937	23448701
12127510	17314962	21152650	23510502
12443277	17499754	21311092	23918719
12475314	17541669	21338070	23930656
12576555	17994599	21405595	24080467
12633474	18541463	21504058	24188923
15171622	18701402	21555541	24422544
15656611	18756556	21740615	24508130
15755103	19364123	22060480	24664042
15852141	20094080	22098088	24697300
16089518	20390172	22463266	24834474
16156622	20596287	22561928	24875287
16350274	20614107	22655006	---
